# Sulfated Polyhydroxysteroid Glycosides from the Sea of Okhotsk Starfish *Henricia leviuscula spiculifera* and Potential Mechanisms for Their Observed Anti-Cancer Activity against Several Types of Human Cancer Cells

**DOI:** 10.3390/md22070294

**Published:** 2024-06-26

**Authors:** Alla A. Kicha, Dmitriy K. Tolkanov, Timofey V. Malyarenko, Olesya S. Malyarenko, Alexandra S. Kuzmich, Anatoly I. Kalinovsky, Roman S. Popov, Valentin A. Stonik, Natalia V. Ivanchina, Pavel S. Dmitrenok

**Affiliations:** 1 G.B. Elyakov Pacific Institute of Bioorganic Chemistry, Far Eastern Branch, Russian Academy of Sciences, Pr. 100-let Vladivostoku 159, 690022 Vladivostok, Russia; tolkanov.dk@gmail.com (D.K.T.); malyarenko-tv@mail.ru (T.V.M.); malyarenko.os@gmail.com (O.S.M.); assavina@mail.ru (A.S.K.); kaaniw@piboc.dvo.ru (A.I.K.); prs_90@mail.ru (R.S.P.); stonik@piboc.dvo.ru (V.A.S.); ivanchina@piboc.dvo.ru (N.V.I.); 2 Department of Bioorganic Chemistry and Biotechnology, School of Natural Sciences, Far Eastern Federal University, Russky Island, Ajax Bay, 10, 690922 Vladivostok, Russia

**Keywords:** steroid, glycoside, sulfate, NMR spectra, sea star, *Henricia leviuscula spiculifera*, anti-proliferative activity, colony formation, cell cycle arrest, MAPK

## Abstract

Three new monosulfated polyhydroxysteroid glycosides, spiculiferosides A (**1**), B (**2**), and C (**3**), along with new related unsulfated monoglycoside, spiculiferoside D (**4**), were isolated from an ethanolic extract of the starfish *Henricia leviuscula spiculifera* collected in the Sea of Okhotsk. Compounds **1**–**3** contain two carbohydrate moieties, one of which is attached to C-3 of the steroid tetracyclic core, whereas another is located at C-24 of the side chain of aglycon. Two glycosides (**2**, **3**) are biosides, and one glycoside (**1**), unlike them, includes three monosaccharide residues. Such type triosides are a rare group of polar steroids of sea stars. In addition, the 5-substituted 3-OSO_3_-α-L-Araf unit was found in steroid glycosides from starfish for the first time. Cell viability analysis showed that **1**–**3** (at concentrations up to 100 μM) had negligible cytotoxicity against human embryonic kidney HEK293, melanoma SK-MEL-28, breast cancer MDA-MB-231, and colorectal carcinoma HCT 116 cells. These compounds significantly inhibited proliferation and colony formation in HCT 116 cells at non-toxic concentrations, with compound **3** having the greatest effect. Compound **3** exerted anti-proliferative effects on HCT 116 cells through the induction of dose-dependent cell cycle arrest at the G2/M phase, regulation of expression of cell cycle proteins CDK2, CDK4, cyclin D1, p21, and inhibition of phosphorylation of protein kinases c-Raf, MEK1/2, ERK1/2 of the MAPK/ERK1/2 pathway.

## 1. Introduction

Sea stars, animals belonging to the phylum Echinodermata, produce varied low molecular weight metabolites, the chemical structures that differ significantly from the metabolites of various representatives of terrestrial flora and fauna. The most abundant natural products in starfish were established to be sterols, polyhydroxysteroids, steroid glycosides, ceramides, cerebrosides, and gangliosides [1,2,3,4,5,6,7]. These secondary metabolites have been reported to exhibit diverse biological activities such as cytotoxic [1], antitumor [8,9,10], anti-inflammatory [11], neuritogenic [12], etc. [6,7,13,14,15].

Starfish of the genus *Henricia* Gray, 1840 (order Spinulosida, family Echinasteridae) inhabit mainly temperate and arctic waters. About 50 species of starfish belong to this genus. *Henricia* spp. are widely distributed in the North Pacific Ocean, especially in the Bering and Okhotsk Seas. Species of the genus *Henricia* are highly variable. Many of them are very close to each other, making their identification difficult in some cases [16,17]. It is known that sea stars contain a variety of polar steroid metabolites. So far, efforts have been made to study the steroid composition of seven species of the genus, namely, *H. leviuscula* (earlier erroneously named as *H. laeviuscola*) [5,18], *H. downeyae* [19,20], *H. sanguinolenta*, *H. leviuscula leviuscula*, *H. aspera*, *H tumida*, and *H. derjugini* [5]. Most of the isolated polar steroids are either sulfated or non-sulfated polyhydroxysteroids or structurally related to them monoglycosides with a monosaccharide residue at C-3 of the aglycon or so-called “two-chains” glycosides with two monosaccharide units attached at different positions of the aglycon, namely, in the steroid nucleus at C-3 and in the steroid side chain at C-24. Summarily, these studies demonstrate a high diversity of polar steroids in sea stars of the genus *Henricia*, which is consistent with the wide biological variability of species in this genus. However, “classical” asterosaponins, which are monosulfated steroid oligoglycosides with five to six monosaccharide residues, were not found in the species studied, with the exception of henricioside A from *H. leviuscula* [18].

Steroid metabolites isolated from sea stars of the genus *Henricia* exhibited diverse biological effects. Thus, some compounds have shown antifungal activity [18], the ability to inhibit cell division of fertilized sea urchin eggs [20], cytotoxic activity against non-small-cell lung human carcinoma [19], and hemolytic effect against mouse erythrocytes [5]. In addition, leviusculoside G from *H. leviuscula* was shown to induce apoptosis in cancer cells and decrease the pro-carcinogenic transformation of normal cells. A possible molecular mechanism was proposed through the induction of p53-dependent apoptosis and inhibition of AP-1, NF-κB, and ERKs activities. Thereby, steroid metabolites isolated from *Henricia* spp. are of interest for further study of their structures and biological activity, especially as anti-cancer and cancer-preventive compounds [21].

In continuation of the study on the chemical constituents of the sea stars [5], herein we report the results of our investigation of polar steroid metabolites from an ethanolic extract of the Far Eastern starfish *Henricia leviuscula spiculifera* H.L. Clark, 1901 (order Spinulosida, family Echinasteridae), collected near Urup Island (Kuril Islands) in the Sea of Okhotsk. We have isolated and structurally elucidated four new polyhydroxysteroid glycosides **1**–**4**. The anti-cancer activity of **1**–**3** against several types of human cancer cells has been investigated. A bioassay of compound **4** was not carried out since it was isolated in insufficient amounts. In addition, the influence of **3**, the most active of the tested compounds, on the cell cycle, regulation of expression of cell cycle proteins, and inhibition of phosphorylation of protein kinases has been studied.

## 2. Results and Discussion

### 2.1. Structure Determination of Compounds ***1***–***4***

**Figure 1:**
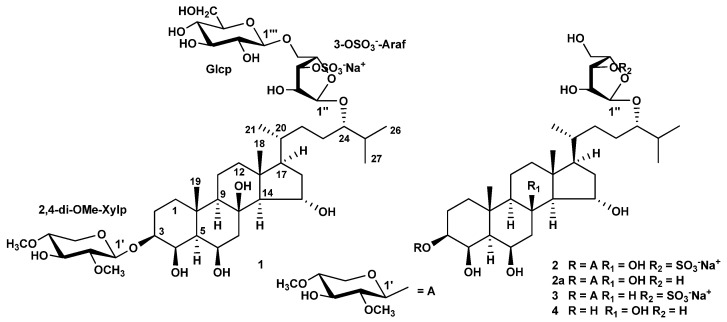
The chemical structures of spiculiferosides A (**1**), B (**2**), C (**3**), and D (**4**).

Three new monosulfated polyhydroxysteroid glycosides and one new unsulfated related monoside were isolated from an ethanolic extract of the sea star *Henricia spiculifera* by means of chromatographic techniques (column chromatography on Polychrom 1, Si gel, and Florisil followed by reverse-phase high-pressure liquid chromatography on Diasfer-110-C18 and YMC-Pack Pro C18 columns). These substances were designated as spiculiferosides A (**1**), B (**2**), C (**3**), and D (**4**) (Figure 1).

Spiculiferoside A (**1**) has the molecular formula C_45_H_77_O_22_SNa determined from the peak of [M − Na]^−^ ion at *m*/*z* 1001.4622 in the (–)HRESIMS and from the peak of the cationized molecule [M + Na]^+^ at *m*/*z* 1047.4419 in the (+)HRESIMS. The fragment ion peak at *m*/*z* 97 [HSO_4_]^−^ in the (−)ESIMS/MS spectrum of the ion with *m*/*z* 1001 [M − Na]^−^ and the fragment ion peaks at *m*/*z* 927 [(M + Na) − NaHSO_4_]^+^ and 143 [Na_2_HSO_4_]^+^ in the (+)ESIMS/MS spectrum of the ion with *m*/*z* 1047 [M + Na]^+^ showed the presence of a sulfate group in **1** (Figure S1). The IR spectrum of **1** revealed absorption bands due to hydroxy (3441 and 1641 cm^−1^) and sulfate (1265 cm^−1^) groups. The ^13^C-NMR and DEPT spectra of **1** exhibited the presence of 45 carbon atoms in the molecule, including 5 methyls, 11 methylenes, 24 methines, two quaternary carbon atoms, one oxygenated tertiary carbon, and two methoxyl groups (Figures S2 and S3). The ^1^H- and ^13^C-NMR spectra of **1** (Table 1 and Table 2, Figures S2 and S4) contained signals of protons and carbon atoms of two angular methyl groups (δ_H_ 0.95, 1.43, both s; δ_C_ 15.3, 18.7 ppm, H_3_C-18, H_3_C-19, respectively), five oxygenated methine groups (δ_H_ 3.64, m; δ_C_ 80.6, HC-3), (δ_H_ 4.25, m; δ_C_ 74.7, HC-4), (δ_H_ 4.25, m; δ_C_ 76.2, HC-6), (δ_H_ 4.27, td, *J* = 9.5, 3.1 Hz; δ_C_ 70.1, HC-15), (δ_H_ 3.31, m; δ_C_ 84.0, HC-24), and one oxygenated tertiary carbon atom (δ_C_ 76.8, C-8), characteristic of 3β,4β,6β,8,15α,24-hexahydroxysteroid aglycon, glycosylated at the positions C-3 and C-24, which was previously found in forbeside J from the starfish *Asterias forbesi* [22].

**Figure 2:**
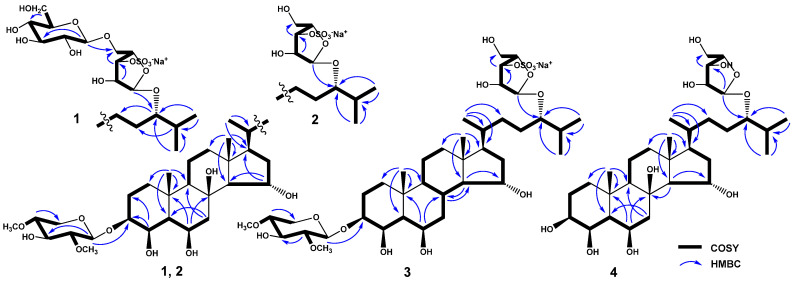
^1^H-^1^H COSY and main HMBC correlations of steroid glycosides **1**–**4**.

**Figure 3:**
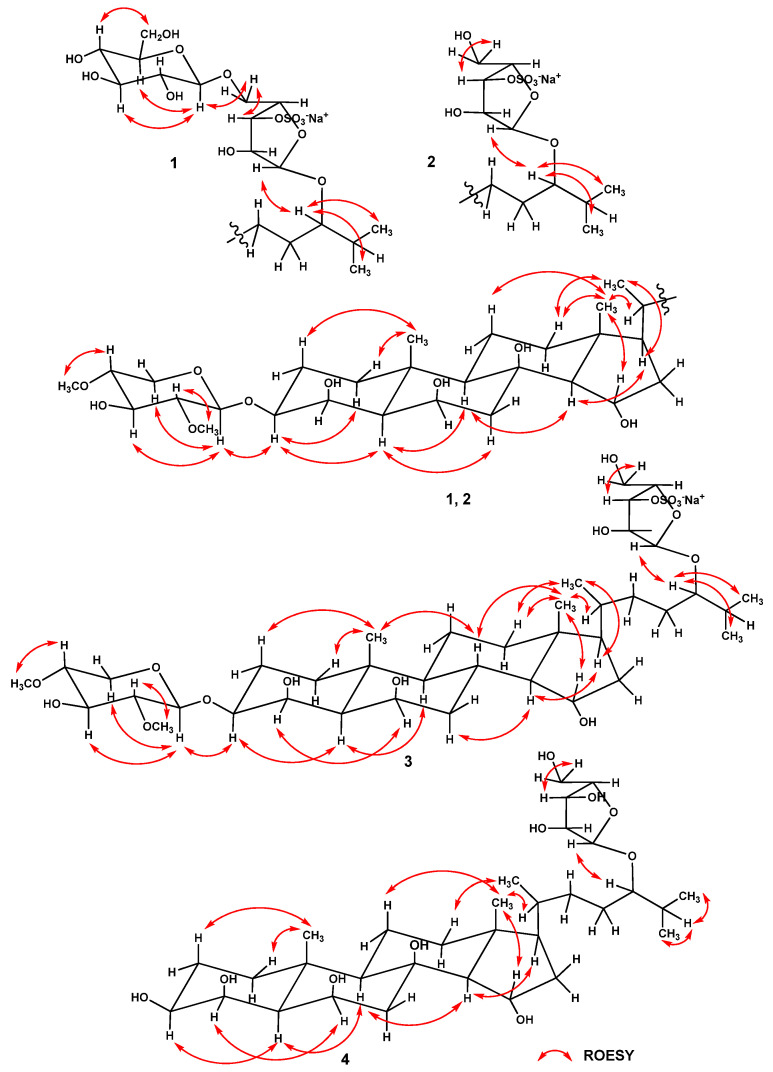
Main ROESY correlations for steroid glycosides **1**–**4**.

Analysis of the ^1^H-^1^H COSY and HSQC correlations made it possible to establish the spin systems of protons and the corresponding sequences of carbon atoms from C-1 to C-7, from C-9 to C-12 through C-11, from C-14 to C-17, from C-20 to C-21, and from C-22 to C-27 (Figure 2, Figures S5 and S6). The relative configurations 3β, 4β, 6β, and 15α of hydroxyl substituents in the steroid core and the 5α-cholestane skeleton in **1** were determined based on proton correlations in the ROESY spectrum from H-3 to Hα-1 and H-5; from H-5 to Hα-7 and H-9; from H-14 to H-9 and H-17; from H_3_-18 to Hβ-11, Hβ-12, and H-15; and from H_3_-19 to Hβ-1, and Hβ-2 (Figure 3 and Figure S7). The main cross peaks of protons and carbon atoms in the HMBC spectrum confirmed the general structure of the steroid aglycon in **1** (Figure 2 and Figure S8). The resonance value of the methyl group H_3_-21 at δ_H_ 0.90, as well as the presence in the ROESY spectrum of correlations of protons from Hβ-12 to H_3_-21, from H-17 to H_3_-21, and from H_3_-18 to H-20, indicated a 20*R* configuration of the asymmetric center [23,24].

In the ^1^H-NMR spectrum of **1**, three chemical shifts of anomeric protons were observed at δ_H_ 4.44, 5.00, and 4.34, associated with signals of carbon atoms at δ_C_ 102.6, 109.2, and 104.9 in the HSQC spectrum, respectively. These data indicated the existence of three monosaccharide residues in glycoside **1**. The coupling constants 7.5 and 7.7 Hz of two anomeric protons exhibited the β-glycosidic bonds of the corresponding monosaccharide residues, and a wide singlet of the third anomeric proton showed the presence of an α-glycosidic bond in this monosaccharide residue. The ESIMS/MS spectrum of the [M − Na]^−^ ion with *m*/*z* 1001 revealed fragment ion peaks corresponding to the loss in a hexose at *m*/*z* 839 [(M − Na) – C_6_H_10_O_5_]^−^ and the simultaneous loss in a hexose and a di-*O*-methyl-pentose at *m*/*z* 679 [(M − Na) − C_6_H_10_O_5_ − C_7_H_12_O_4_]^−^. Respectively, the ESIMS/MS spectrum of the [M + Na]^+^ ion with *m*/*z* 1047 exhibited fragment ion peaks arising due to the loss in a hexose at *m*/*z* 885 [(M + Na) − C_6_H_10_O_5_]^+^, the simultaneous loss in a hexose and a di-*O*-methyl-pentose at *m*/*z* 725 [(M + Na) − C_6_H_10_O_5_ − C_7_H_12_O_4_]^+^, the simultaneous loss in a hexose and a sulfoxypentose at *m*/*z* 651 [(M + Na) − C_6_H_10_O_5_ − C_5_H_7_O_7_SNa]^+^, and the simultaneous loss in a hexose, a sulfoxypentose, and a di-*O*-methyl-pentose at *m*/*z* 491 [651 − C_7_H_12_O_4_]^+^ (Figure S1). Therefore, according to the ESIMS/MS and NMR spectra, molecule **1** contains hexose, di-*O*-methyl-pentose, and sulfoxypentose units. Acid hydrolysis of glycoside **1** with 2 M CF_3_COOH yielded three monosaccharides, which, after obtaining 2-octylglycoside derivatives by treatment with (*R*)-(–)-2-octanol and subsequent acetylation according to the procedure of Leontein et al. [25], were identified by GC as 2,4-di-*O*-methyl-D-xylose, L-arabinose, and D-glucose.

The sequences of protons and the carbon atoms associated with corresponding protons, as well as the relative proton configurations of monosaccharide residues, were assigned using ^1^H-^1^H COSY, HSQC, HMBC, and ROESY experiments (Table 2, Figure 2 and Figure 3). Irradiation of anomeric protons in 1D TOCSY experiments allowed us to refine the chemical shifts and coupling constants of the carbohydrate moiety protons. The spectral data of the two monosaccharide units were in good agreement with those for the terminal residues of 2,4-di-*O*-methyl-β-d-xylopyranose [22] and β-d-glucopyranose [26]. The position of the terminal 2,4-di-*O*-methyl-β-d-xylopyranose unit at C-3 of aglycon was confirmed by the cross-peaks between H-1′ and H-3, C-3, the linkage of the terminal residue of β-d-glucopyranose to C-5″ of the internal residue of α-L-arabinofuranose was indicated by the cross-peaks between H-1‴ and H_2_-5″, C-5″, and the linkage of the α-L-arabinofuranose residue to C-24 of aglycon was fixed by the cross-peaks between H-1″ and H-24, C-24 in the ROESY and HMBC spectra, respectively. A comparison of the proton and carbon signals of the internal monosaccharide residue of glycoside **1** with those of the five-substituted α-l-arabinofuranose residue of kurilensoside B from the starfish *Hippasteria kurilensis* [27] showed that the chemical shifts of H-3″ and C-3″ were deshielded from δ_H_ 3.94 to 4.67 and from δ_C_ 79.2 to 84.7, respectively, and the signal of C-2″ was shielded from δ_C_ 83.8 to 82.0. These facts clearly revealed the location of the sulfate group at C-3″of 5″-substituted residue of α-l-arabinofuranose in **1**. In addition, the signal of C-1″ at δ_C_ 109.2 unambiguously indicated the α-configuration of the anomeric center of the arabinofuranose residue [28]. The 24*S* configuration was proposed based on the similarity of the ^13^C-NMR spectroscopic data for the side chain of glycoside **1** with those for other related (24*S*)-24-*O*-α-l-arabinofuranosides previously isolated from starfish [29,30,31]. Consequently, the structure of spiculiferoside A (**1**) was elucidated as the (24*S*)-3-*O*-(2,4-di-*O*-methyl-β-d-xylopyranosyl)-24-*O*-[β-D-glucopyranosyl-(1→5)-3-*O*-sulfate-α-l-arabinofuranosyl]-5α-cholestane-3β,4β,6β,8,15α,24-hexaol, sodium salt. Glycoside **1** is a triglycoside and contains two carbohydrate moieties, one of which is attached to C-3 of the steroid core, and the other is located at C-24 of the aglycon side chain. Only five such “two-chain” triglycosides from see stars were previously known [27,32,33]. In addition, the five-substituted 3-OSO_3_-α-l-Araf residue was found for the first time in steroid glycosides from starfish.

Spiculiferoside B (**2**) has the molecular formula C_39_H_67_O_17_SNa, determined from the peak of [M − Na]^−^ ion at *m*/*z* 839.4108 in the (–)HRESIMS and from the peak of cationized molecule [M + Na]^+^ at *m*/*z* 885.3896 in the (+)HRESIMS. The fragment ion peak at *m*/*z* 97 [HSO_4_]^−^ in the (−)ESIMS/MS spectrum of the ion with *m*/*z* 839 [M − Na]^−^ and the fragment ion peaks at *m*/*z* 765 [(M + Na) − NaHSO_4_]^+^ and 143 [Na_2_HSO_4_]^+^ in the (+)ESIMS/MS spectrum of the ion with *m*/*z* 885 [M + Na]^+^ showed the presence of a sulfate group in **2** (Figure S9). The IR spectrum of **2** revealed absorption bands due to hydroxy (3440 and 1632 cm^−1^) and sulfate (1263 cm^−1^) groups.

The ^1^H-NMR spectrum of **2** included two resonances in the deshielded region due to anomeric protons at δ_H_ 4.44 and 4.98, which correlated in the HSQC spectrum with corresponding carbon resonances at δ_C_ 102.7 and 109.5, respectively (Table 2). The ESIMS/MS spectrum of the [M − Na]^−^ ion with *m*/*z* 839 indicated fragment ion peaks corresponding to the loss in a di-*O*-methyl-pentose at *m*/*z* 679 [(M − Na) − C_7_H_12_O_4_]^−^ and a sulfoxypentose at *m*/*z* 211 [C_5_H_7_O_7_S]^−^. Accordingly, the ESIMS/MS spectrum of the [M + Na]^+^ ion with *m*/*z* 885 revealed fragment ion peaks arising due to the loss in a di-*O*-methyl-pentose at *m*/*z* 725 [(M + Na) − C_7_H_12_O_4_]^+^, the loss in a sulfoxypentose at *m*/*z* 651 [(M + Na) − C_5_H_7_O_7_SNa]^+^, and the simultaneous loss in a di-*O*-methyl-pentose and a sulfoxypentose at *m*/*z* 491 [651 − C_7_H_12_O_4_]^+^ (Figure S9). A detailed comparison of the ^1^H-, ^13^C-NMR, DEPT, COSY, HSQC, HMBC, and ROESY spectroscopic data of glycoside **2** (Table 1 and Table 2, Figure 2, Figure 3, and Figures S10–S16) with the corresponding data of glycoside **1** showed that **2** had the same 3β,4β,6β,8,15α,24-hexahydroxy-5α-cholestane aglycon, glycosylated at C-3 with a 2,4-di-*O*-methyl-β-d-xylopyranose residue, and at C-24 with a sulfated α-l-arabinofuranose residue, and differed from **1** only in the absence of a terminal β-d-glucopyranose residue. A comparison of the signals of the protons and carbon atoms of the monosaccharide unit at C-24 in glycoside **2** with the corresponding signals of the terminal α-L-arabinofuranose residue of forbeside J [22] showed that the resonance of C-3″ was deshielded from δ_C_ 78.7 to 84.5; the resonances of C-2″ and C-4″ were shielded from δ_C_ 84.0 to 82.1 and from δ_C_ 85.0 to 84.4, respectively, and the signal of H-3″ was deshielded from δ_H_ 3.86 to 4.46 in accordance with α- and β-effects of sulfation. In this way, the position of a sulfate group in the α-L-arabinofuranose in **2** was defined as C-3″. Compound **2** was subjected to mild solvolysis with a mixture of dioxane and pyridine to give desulfated derivative **2a**, which was identified by comparison of the HRESIMS and ^1^H-, ^13^C-NMR, and HSQC data (Experimental section, Figures S17–S20) with those of forbeside J [22]. As a result, the absolute configuration at C-24 in **2** was proposed as *S* by analogy with forbeside J (**2a**). On the basis of the above-mentioned data, the structure of spiculiferoside B (**2**) was defined as the (24*S*)-3-*O*-(2,4-di-*O*-methyl-β-d-xylopyranosyl)-24-*O*-(3-*O*-sulfate-α-l-arabinofuranosyl)-5α-cholestane-3β,4β,6β,8,15α,24-hexaol sodium salt.

Spiculiferoside C (**3**) has the molecular formula C_39_H_67_O_16_SNa determined from the peak of [M − Na]^−^ ion at *m*/*z* 823.4162 in the (–)HRESIMS and from the peak of cationized molecule [M + Na]^+^ at *m*/*z* 869.3936 in the (+)HRESIMS. The fragment ion peak at *m*/*z* 97 [HSO_4_]^−^ in the (−)ESIMS/MS spectrum of the ion with *m*/*z* 823 [M − Na]^−^ and the fragment ion peaks at *m*/*z* 749 [(M + Na) − NaHSO_4_]^+^ and 143 [Na_2_HSO_4_]^+^ in the (+)ESIMS/MS spectrum of the ion with *m*/*z* 869 [M + Na]^+^ showed the presence of a sulfate group in **3** (Figure S21). The IR spectrum of **3** revealed absorption bands due to hydroxy (3441 and 1641 cm^−1^) and sulfate (1270 cm^−1^) groups.

The ESIMS/MS spectrum of the [M − Na]^−^ ion with *m*/*z* 823 indicated fragment ion peaks corresponding to the loss in a di-*O*-methyl-pentose at *m*/*z* 663 [(M − Na) − C_7_H_12_O_4_]^−^ and a sulfoxypentose at *m*/*z* 211 [C_5_H_7_O_7_S]^−^. Accordingly, the ESIMS/MS spectrum of the [M + Na]^+^ ion with *m*/*z* 869 revealed fragment ion peaks arising due to the loss in a di-*O*-methyl-pentose at *m*/*z* 709 [(M + Na) − C_7_H_12_O_4_]^+^ and the loss in a sulfoxypentose at *m*/*z* 635 [(M + Na) − C_5_H_7_O_7_SNa]^+^(Figure S21). Examination of the HRESIMS, ESIMS/MS, 1D, and 2D NMR spectra of glycoside **3** and the corresponding data of glycoside **2** clearly showed the presence of identical monosaccharide residues and steroid side chain in both compounds: 2,4-di-*O*-methyl-β-d-xylopyranose unit attached to C-3 of steroid nucleus and 3-sulfoxy-α-l-arabinofuranose unit attached to C-24 of the steroid side chain (Table 1 and Table 2).

Most of the resonances in the ^1^H- and ^13^C-NMR spectra of **3**, related to the steroid moiety, were close to the corresponding values for **2** (Table 1, Figures S22 and S23). However, in the ^13^C-NMR spectrum of **3,** no signal was observed for carbon atom C-8 at δ_C_ 76.8 because of the absence of a hydroxyl group at this position. ^1^H-^1^H COSY, HSQC, and HMBC cross-peaks supported the presence of spin proton sequences in the steroid core of **3** at C-1 to C-9, at C-9 to C-12 through C-11, at C-8 to C-14, and at C-14 to C-17 (Figure 2 and Figures S25–S27). The ROESY correlations from H-4 to H-6, from H-5 to H-3 and H-9, from Hα-7 to H-14, from H-8 to H_3_-18 and H_3_-19, from H_3_-18 to Hβ-12 and H-15, and from H_3_-19 to Hβ-1 and Hβ-2 confirmed that the 5α/8β/9α/10β/13β/14α steroid nucleus in **3** had a 3β,4β,6β,15α-tetrahydroxy substitution (Figure 3 and Figure S28). Thereby, the structure of spiculiferoside C (**3**) was established as the (24*S*)-3-*O*-(2,4-di-*O*-methyl-β-d-xylopyranosyl)-24-*O*-(3-*O*-sulfate-α-l-arabinofuranosyl)-5α-cholestane-3β,4β,6β,15α,24-pentaol sodium salt. The 3-OSO_3_-α-l-Araf residue was included in spiculiferosides B (**2**) and C (**3**) and was previously found in only one steroid glycoside from starfish *Oreaster reticulatus* [34].

The molecular formula C_32_H_56_O_10_ of spiculiferoside D (**4**) was elucidated from the peaks of [M − H]^−^ ion at *m*/*z* 599.3794, [M + Cl]^−^ ion at *m*/*z* 635.3561, and [M + CHO_2_]^−^ ion at *m*/*z* 645.3847 in the (–)HRESIMS and from the peak of the cationized molecule [M + Na]^+^ at *m*/*z* 623.3770 in the (+)HRESIMS. The fragment ion peaks at *m*/*z* 467 [(M − H) − C_5_H_8_O_4_]^−^, 449 [(M − H) − C_5_H_10_O_5_]^−^, and 131 [C_5_H_7_O_4_]^−^ in the (−)ESIMS/MS spectrum of the ion with *m*/*z* 599 [M − H]^−^ and the fragment ion peaks at *m*/*z* 491 [(M + Na) − C_5_H_8_O_4_]^+^ and 473 [(M + Na) − C_5_H_10_O_5_]^+^ in the (+)ESIMS/MS spectrum of the ion with *m*/*z* 623 [M + Na]^+^ indicated the presence of a pentose unit in **4** (Figure S29). A thorough comparison of NMR spectra of compound **4** and desulfated derivative **2a** (Experimental section) exhibited that both compounds contained the same 3β,4β,6β,8,15α,24-hexahydroxy-5α-cholestane aglycon and α-L-arabinofuranose residue at C-24 of the side chain and differed from each other only in the absence of a 2,4-di-*O*-methyl-β-d-xylopyranose residue at C-3 of the steroid moiety of **4**. In accordance with this, the chemical shifts of H-3 and C-3 of **4** compared to **2a** were shielded from δ_H_ 3.64 to 3.50 and from δ_C_ 80.5 to 73.1, respectively, while the signals of C-2 and C-4 were deshielded from δ_C_ 25.2 to 26.6 and from δ_C_ 74.6 to 77.5, respectively, according to α- and β-effects of deglycosylation (Figures S30 and S31). The structure of glycoside **4** was confirmed by DEPT, ^1^H-^1^H COSY, HSQC, HMBC, and ROESY experiments (Figure 2, Figure 3, and Figures S32–S36). Thus, it was established that spiculiferoside D (**4**) was the (24*S*)-24-*O*-(α-L-arabinofuranosyl)-5α-cholestane-3β,4β,6β,8,5α,24-hexaol.

### 2.2. The Effect of Compounds ***1***–***3*** on Cell Viability and Proliferation of Human Normal and Cancer Cells

**Figure 4:**
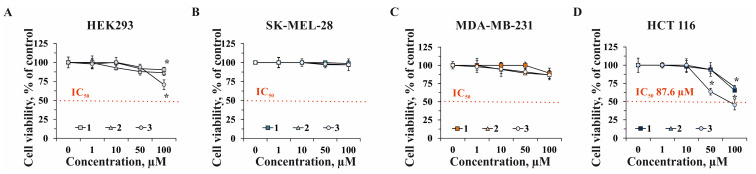
The cytotoxic activity of compounds **1**, **2**, and **3** against human normal and cancer cells. (**A**) Human embryonic kidney HEK293, (**B**) melanoma SK-MEL-28, (**C**) breast cancer MDA-MB-231, and (**D**) colorectal carcinoma HCT 116 cells were treated with **1**, **2**, and **3** (1, 10, 50, and 100 µM) for 24 h. MTS assay was used to evaluate cytotoxicity of compounds. IC_50_—the concentration at which the compounds exert half of their maximal inhibitory effect on cell viability. The data results are presented as mean ± SD for triplicate experiments. A one-way ANOVA and Tukey’s HSD test for multiple comparisons indicated the statistical significance (* *p* < 0.05).

In the present work, the effect of compounds **1**, **2**, and **3** on cell viability of human embryonic kidney HEK293, melanoma SK-MEL-28, breast cancer cells MDA-MB-231, and colorectal carcinoma HCT 116 cells was determined by MTS assay in a 24 h cell treatment (Figure 4).

The tested compounds were less cytotoxic against normal cells, HEK293, and two types of cancer cells, SK-MEL-28 and MDA-MB-231 (Figure 4A–C). On the other hand, these compounds were found to suppress cell viability of colorectal carcinoma cells HCT 116 more effectively, with great impact of compound **3** (Figure 4D). Compound **1** at concentrations of 1, 10, 50, and 100 µM inhibited cell viability of HCT 116 cells by 0%, 2%, 6%, and 35%, respectively; compound **2** inhibited cell viability at 1, 10, 50, and 100 µM—0%, 0%, 6%, and 30%, respectively, while **3** at the same experimental conditions suppressed the cell viability by 0%, 0%, 37%, and 54%, respectively (Figure 4D). IC_50_ was reached only for compound **3**, which was 87.6 µM with a selective index (SI) of 1.5 after 24 h of HCT 116 cells’ treatment (Figure 4D).

**Figure 5:**
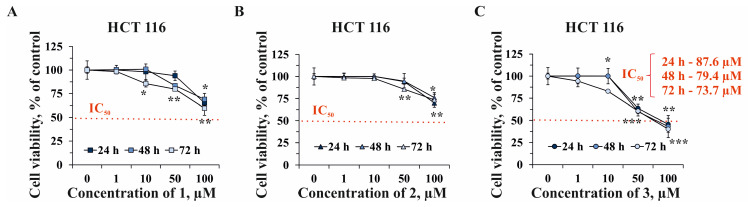
The effect of compounds **1**, **2**, and **3** on the proliferation of human colorectal carcinoma cells HCT 116. HCT 116 cells were treated with compounds **1** (**A**), **2** (**B**), and **3** (**C**) at concentrations of 1, 10, 50, and 100 µM for 24, 48, and 72 h. MTS assay was used to evaluate anti-proliferative activities of compounds. A one-way ANOVA and Tukey’s HSD test for multiple comparisons indicated the statistical significance (* *p* < 0.05; ** *p* < 0.01; *** *p* < 0.001).

Since compounds **1**, **2**, and **3** possessed more significant cytotoxic activity against HCT 116 cells, we determined their effect on the proliferation of HCT 116 cells. All the tested compounds slightly inhibited cell proliferation at concentrations ranging from 1 to 50 µM within 72 h of treatment (Figure 5). Compound **1** at 100 µM decreased cell growth by 35%, 31%, and 40% after 24, 48, and 72 h of cells’ treatment, respectively (Figure 5A). Compound **2** (100 µM) was shown to inhibit cell proliferation by 30%, 25%, and 27% after 24, 48, and 72 h of cell incubation, respectively (Figure 5B). Compound **3** (100 µM) possessed the highest anti-proliferative activity and suppressed proliferation of HCT 116 cells by 55%, 57%, and 60% after 24, 48, and 72 h of treatment, respectively (Figure 5C).

### 2.3. The Effect of Compounds ***1***–***3*** on the Colony Formation of Human Colorectal Carcinoma Cells

**Figure 6:**
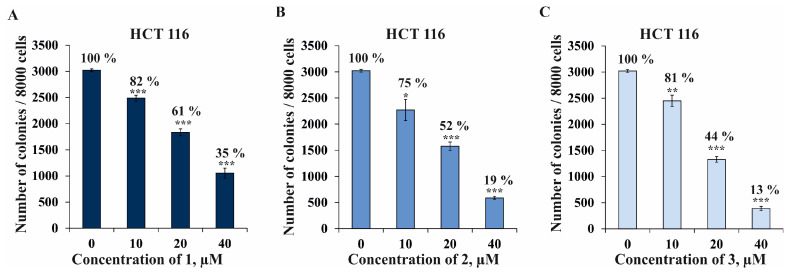
The effect of compounds **1**, **2**, and **3** on the colony formation in human colorectal carcinoma cells HCT 116. HCT 116 cells were treated by **1** (**A**), **2** (**B**), and **3** (**C**) at concentrations of 10, 20, and 40 µM in soft agar. The number of colonies was counted under a microscope (at a total magnification of 40×) using the ImageJ software version 1.50i bundled with 64-bit Java 1.6.0_24 (“NIH”, Bethesda, MD, USA). Results are presented as mean ± standard deviation (SD). A one-way ANOVA and Tukey’s HSD test for multiple comparisons indicated the statistical significance (* *p* < 0.05, ** *p* < 0.01, *** *p* < 0.001).

More promising data were obtained in the results of the studies on the effects of **1**–**3** on microcolony formation by tumor cells. In the present study, the colony-inhibiting activity was investigated in HCT 116 cells using the soft agar assay. Non-toxic concentrations of 10, 20, and 40 µM of the investigated compounds were chosen for further experiments. All the tested compounds were found to significantly decrease colonies’ numbers of colorectal carcinoma cells dose-dependently (Figure 6). Compound **1** at concentrations of 10, 20, and 40 µM inhibited colony formation in HCT 116 cells by 18%, 39%, and 65%, respectively (Figure 6A); **2**—by 25%, 48%, and 81%, respectively (Figure 6B), and **3**—by 19%, 56%, 87%, respectively (Figure 6C). Compound **3** was found to have the most significant colony-inhibiting activity against HCT 116 cells among all the compounds studied and was, therefore, selected for further investigation of the molecular mechanism of its anti-cancer action.

### 2.4. The Effect of Compound ***3*** on Cell Cycle Progression and Molecular Mechanism of Anti-cancer Action in Human Colorectal Carcinoma Cells

The fundamental abnormality that leads to the development of cancer is the continuous, unregulated proliferation of cancer cells. Instead of responding appropriately to signals that control normal cell behavior, cancer cells grow and divide uncontrollably, invading normal tissues and organs and eventually spreading throughout the body [35]. Since compound **3** inhibited proliferation and colony formation of colorectal cancer cells HCT 116, we checked whether compound **3** could regulate cell cycle distribution by flow cytometric analysis. Cell cycle progression was examined after treatment of HCT 116 cells with 10, 20, and 40 μM of **3** for 72 h.

**Figure 7:**
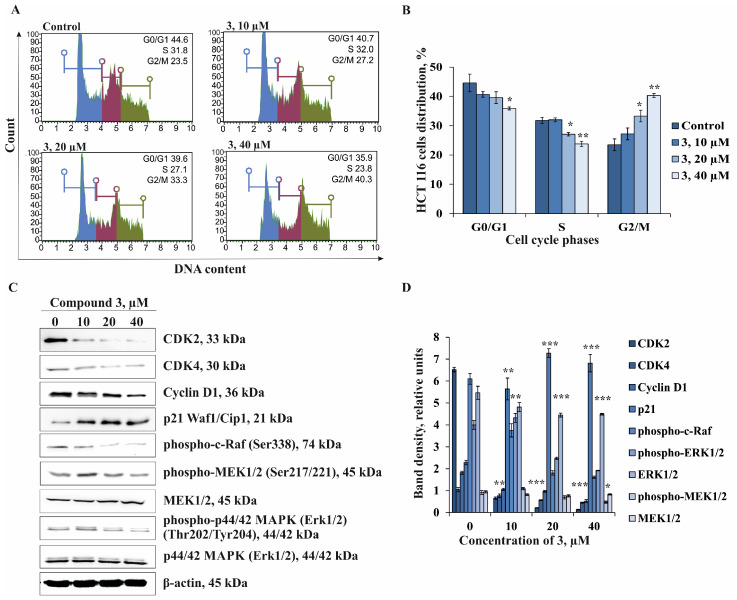
The effect of compound **3** on cell cycle regulation and the expression of cell cycle markers and MAPK kinases in human colorectal carcinoma cells HCT 116. (**A**,**B**) HCT 116 cells were treated with **3** at 10, 20, and 40 µM for 72 h. The percentage of cells in G0/G1, S, and G2/M phases was determined using a Muse cell analyzer. Histograms from a representative experiment show the effect of **3** on cell cycle profile. (**C**) The regulation of expression of cell cycle markers, MAPK, and β-actin by **3** (10, 20, and 40 µM) after 72 h of treatment of HCT 116 cells. (**D**) Relative band density was measured using the Quantity One 1D analysis software version 4.6.7. Band density was normalized to β-actin total level. Results are presented as mean ± standard deviation (SD). A one-way ANOVA and Tukey’s HSD test for multiple comparisons indicated the statistical significance (* *p* < 0.05; ** *p* < 0.01; *** *p* < 0.001).

It was found that the treatment of HCT 116 cells with **3** resulted in a dose-dependent increase in cells in the G2/M phase compared to the control group. Compound **3** at 10, 20, and 40 µM was shown to increase the amount of HCT 116 cells in G2/M phase by 16%, 42%, and 71%, respectively, with a corresponding reduction in the percentage of cells in the G0/G1 phase by 0%, 15%, and 25%, respectively, and S phase by 9%, 11%, and 20%, respectively, compared to the control group (Figure 7A,B). These data suggest that the inhibition of cell proliferation of HCT 116 cells is mainly associated with the induction of G2/M cell cycle arrest.

Next, we turned our attention to the molecular mechanism of anti-cancer action of compound **3** associated with the inhibition of cell proliferation of HCT 116 cells via the regulation of a series of important cell cycle proteins and the activation of mitogen-activated protein kinases (MAPK) by Western Blot assay. Extracellular-signal-related kinase p44/42 MAPK (Erk1/2) is known to be an important participant in the MAPK signaling pathway [36]. ERK1/2 plays a well-established role in regulating cell cycle progression by activation of multiple transcription factors such as Elk1, c-Jun, c-Myc, and c-Fos, which control the expression of proteins important for cell-cycle progression, including Cyclin D1 and p21WAF1/CIP1 [37]. Cyclin-dependent kinases (CDK) are major players in cell proliferation that regulate cell cycle checkpoints and transcription events in response to extracellular and intracellular signals. CDK dysregulation is certain to be a hallmark of cancer and an attractive target in cancer therapy. CDK activity is primarily regulated by the binding of CDK catalytic subunits to Cyclin partners and CDK inhibitors. The complex formed by CDK4 and Cyclin D1 has been strongly implicated in the control of cell proliferation and prognoses in human malignancies [38]. In this regard, we examined the influence of **3** on the expression of CDK2, CDK4, Cyclin D1, and p21. The investigated compound was found to dose-dependently down-regulate the expression of CDK2 and Cyclin D1 but not CDK4. The expression of the inhibitor of CDK/Cyclin complex—p21 was significantly increased by **3** compared to non-treated HCT 116 cells (Figure 7C,D). The treatment of HCT 116 cells by **3** was demonstrated to cause the inhibition of phosphorylation of c-Raf, MEK1/2, and ERK1/2 kinases (Figure 7C,D).

Our results provided evidence that the coordinated alteration of the expression of cell cycle proteins and inhibition of the phosphorylation of the ERK1/2 MAPK signaling cascade were likely the basis of the anti-cancer effect of compound **3** on the proliferation of colorectal carcinoma cells HCT 116.

## 3. Materials and Methods

### 3.1. General Procedures

Optical rotations, Perkin-Elmer 343 polarimeter (PerkinElmer, Waltham, MA, USA). NMR spectra, Bruker Avance III 500 HD (Bruker, Göttingen, Germany) at 500.13 MHz (^1^H)/125.76 MHz (^13^C), Bruker Avance III 700 spectrometer (Bruker, Bremen, Germany) at 700.13 (^1^H)/176.04 MHz (^13^C), internal standard CD_3_OD at *δ*_H_ 3.30/*δ*_C_ 49.0. HRESIMS spectra, Bruker Impact II Q-TOF mass spectrometer (Bruker, Bremen, Germany); sample concentration in MeOH 0.001 mg/mL. HPLC, Agilent 1100 Series chromatograph (Agilent Technologies, Santa Clara, CA, USA) with a differential refractometer; columns Discovery C18 (5 µm, 10.0 × 250 mm, Supelco, Bellefonte, PA, USA), YMC-Pack Pro C18 (5 µm, 10.0 × 250 mm, YMC Co., Ltd., Kyoto, Japan), and Diasfer-110-C18 (5 µm, 4.0 × 250 mm, BioChemMack, Moscow, Russia). GC, Agilent 6580 Series chromatograph (Agilent Technologies, Santa Clara, CA, USA), HP-1 MS capillary column (0.32 mm × 30 m) over the temperature range 100−270 °C at 5 °C/min, carrier gas He (1.7 mL/min), injector temperature 250 °C, detector temperature 270 °C. LPLC, column sorbents Polychrom 1 (powdered Teflon, 0.25–0.50 mm, Biolar, Olaine, Latvia), Si gel (63–200 µm, Sigma-Aldrich, Switzerland), and Florisil (60–100 µm, Sigma-Aldrich, Co., St. Louis, MI, USA).

### 3.2. Animal Material

Specimens of *Henricia leviuscula spiculifera* Clark, 1901 (order Spinulosida, family Echinasteridae) were collected near Urup Island (Kuril Islands, Sea of Okhotsk) at a depth of 85–89 m using a small trawl (research vessel *Akademik Oparin*, 51th scientific cruise, May 2017). Taxonomical identification of species was determined by Mr. Boris B. Grebnev (G.B. Elyakov PIBOC FEB RAS, Vladivostok, Russia). A voucher specimen (no. 051-039a) has been deposited in the collection of G.B. Elyakov PIBOC FEB RAS, Vladivostok, Russia.

### 3.3. Extraction and Isolation

Freshly collected specimens of starfish *H. leviuscula spiculifera* were immediately frozen after fishing. The sliced specimens (1.1 kg) were extracted twice with EtOH (2.0 L/kg) at room temperature. The extract was evaporated under reduced pressure, and the residue (83.2 g) was dissolved in H_2_O (0.5 L). The H_2_O-soluble fraction was passed through a Polychrom 1 column (7.5 × 75 cm) and eluted with H_2_O and then with EtOH. The combined EtOH eluate was concentrated under reduced pressure, and the resulting total fraction (9.7 g) was chromatographed over a Si gel column (6.5 × 15 cm) using CHCl_3_/EtOH (stepwise gradient, 5:1−1:3, *v*/*v*). The obtained fractions were further purified on Florisil columns (7 × 15 cm) using CHCl_3_/EtOH (stepwise gradient, 3:1 to 1:3, *v*/*v*) to yield four main fractions (1–4). Fr. 1 (242 mg) was subjected to HPLC on a Discovery C18 column (65% aq. EtOH, flow rate: 2.6 mL/min) and further separated on a YMC-Pack Pro C18 column (80% aq. MeOH, flow rate: 1.1 mL/min) to afford pure **4** (2.3 mg, *t*_R_ 16.9 min). Fr. 2 (641 mg) was separated by HPLC on a Discovery C18 column (MeOH/H_2_O/1M NH_4_OAc, 55:44:1, *v*/*v*/*v*, flow rate: 1.7 mL/min) to give pure **2** (44 mg, *t*_R_ 24.3 min) and **3** (17 mg, *t*_R_ 36.7 min). Fr. 3 (235 mg) was subjected to HPLC on a YMC-Pack Pro C18 column (54% aq. EtOH, flow rate: 2.0 mL/min) and purified repeatedly on the same column (50% aq. EtOH, flow rate: 2.0 mL/min) to afford pure **1** (14 mg, *t*_R_ 13.9 min).

### 3.4. Compound Characterization Data

Spiculiferoside A (**1**): Colorless powder; [α]_D_^25^: −27.6 (*c* 0.43, MeOH); IR (KBr) *ν*_max_ 3441, 2931, 1641, 1448, 1422, 1265, 1081, 1046 cm^−1^; (−)HRESIMS *m*/*z* 1001.4622 [M − Na]^−^ (calcd for C_45_H_77_O_22_S, 1001.4633); (+)HRESIMS *m*/*z* 1047.4419 [M + Na]^+^ (calcd for C_45_H_77_O_22_SNa_2_, 1047.4417); (−)ESIMS/MS of the [M − Na]^−^ ion with *m*/*z* 1001: 839 [(M − Na) − C_6_H_10_O_5_]^−^, 679 [(M − Na) − C_6_H_10_O_5_ − C_7_H_12_O_4_]^−^, 661 [(M − Na) − C_6_H_10_O_5_ − C_7_H_12_O_4_ −H_2_O]^−^, 97 [HSO_4_]^−^; (+)ESIMS/MS of the [M + Na]^+^ ion with *m*/*z* 1047: 927 [(M + Na) − NaHSO_4_]^+^, 885 [(M + Na) − C_6_H_10_O_5_]^+^, 725 [(M + Na) − C_6_H_10_O_5_ − C_7_H_12_O_4_]^+^, 651 [(M + Na) − C_6_H_10_O_5_ − C_5_H_7_O_7_SNa]^+^, 633 [651 − H_2_O]^+^, 491 [651 − C_7_H_12_O_4_]^+^, 143 [Na_2_HSO_4_]^+^; ^1^H- and ^13^C-NMR data of aglycon moiety, see Table 1; ^1^H- and ^13^C-NMR data of carbohydrate moiety, see Table 2.

Spiculiferoside B (**2**): Colorless powder; [α]_D_^25^: –27.8 (*c* 0.71, MeOH); IR (KBr) *ν*_max_ 3440, 2930, 1632, 1446, 1423, 1263, 1088, 1046, 1024, 979 cm^−1^; (−)HRESIMS *m*/*z* 839.4108 [M − Na]^−^ (calcd for C_39_H_67_O_17_S, 839.4104); (+)HRESIMS *m*/*z* 885.3896 [M + Na]^+^ (calcd for C_39_H_67_O_17_SNa_2_, 885.3889); (−)ESIMS/MS of the [M − Na]^−^ ion with *m*/*z* 839: 679 [(M − Na) − C_7_H_12_O_4_]^−^, 661 [(M − Na) − C_7_H_12_O_4_ − H_2_O]^−^, 211 [C_5_H_7_O_7_S]^−^, 152 [C_3_H_5_O_5_S]^−^, 97 [HSO_4_]^−^; (+)ESIMS/MS of the [M + Na]^+^ ion with *m*/*z* 885: 765 [(M + Na) − NaHSO_4_]^+^, 725 [(M + Na) − C_7_H_12_O_4_]^+^, 651 [(M + Na) − C_5_H_7_O_7_SNa]^+^, 633 [651 − H_2_O]^+^, 491 [651 − C_7_H_12_O_4_]^+^, 143 [N_2_HSO_4_]^+^; ^1^H- and ^13^C-NMR data of aglycon moiety, see Table 1; ^1^H- and ^13^C-NMR data of carbohydrate moiety, see Table 2.

Spiculiferoside C (**3**): Colorless powder; [α]_D_^25^: –17.6 (*c* 0.6, MeOH); IR (KBr) *ν*_max_ 3441, 2938, 1641, 1458, 1423, 1270, 1087, 1045, 984 cm^−1^; (−)HRESIMS *m*/*z* 823.4162 [M − Na]^−^ (calcd for C_39_H_67_O_16_S, 823.4155); (+)HRESIMS *m*/*z* 869 [M + Na]^+^ (calcd for C_39_H_67_O_16_SNa_2_, 869.3940); (−)ESIMS/MS of the [M − Na]^−^ ion with *m*/*z* 823: 663 [(M − Na) − C_7_H_12_O_4_]^−^, 645 [(M − Na) − C_7_H_12_O_4_ − H_2_O]^−^, 211 [C_5_H_7_O_7_S]^−^, 152 [C_3_H_5_O_5_S]^−^, 97 [HSO_4_]^−^; (+)ESIMS/MS of the [M + Na]^+^ ion with *m*/*z* 869: 749 [(M + Na) − NaHSO_4_]^+^, 709 [(M + Na) − C_7_H_12_O_4_]^+^, 635 [(M + Na) − C_5_H_7_O_7_SNa]^+^, 617 [635 − H_2_O]^+^, 143 [Na_2_HSO_4_]^+^; ^1^H- and ^13^C-NMR data of aglycon moiety, see Table 1; ^1^H- and ^13^C-NMR data of carbohydrate moiety, see Table 2.

Spiculiferoside D (**4**): Colorless powder; [α]_D_^25^: –11.0 (*c* 0.21, MeOH); (−)HRESIMS *m*/*z* 599.3794 [M − H]^−^ (calcd for C_32_H_55_O_10_, 599.3801), 635.3561 [M + Cl]^−^ (calcd for C_32_H_56_O_10_Cl, 635.3567), 645.3847 [M + CHO_2_]^−^ (calcd for C_33_H_57_O_12_, 645.3856); (+)HRESIMS *m*/*z* 623.3770 [M + Na]^+^ (calcd for C_32_H_56_O_10_Na, 623.3766); (−)ESIMS/MS of the [M − H]^−^ ion with *m*/*z* 599: 467 [(M − H) − C_5_H_8_O_4_]^−^, 449 [(M − H) − C_5_H_10_O_5_]^−^, 131 [C_5_H_7_O_4_]^−^; (+)ESIMS/MS of the [M + Na]^+^ ion with *m*/*z* 623: 605 [(M + Na) –H_2_O]^+^, 491 [(M + Na) − C_5_H_8_O_4_]^+^, 473 [(M + Na) − C_5_H_10_O_5_]^+^; ^1^H-NMR (CD_3_OD, 700.13 MHz): *δ*_H_ 0.90 (d, *J* = 6.9 Hz, H_3_-26), 0.90 (d, *J* = 6.9 Hz, H_3_-27), 0.91 (d, *J* = 6.0 Hz, H_3_-21), 0.95 (s, H_3_-18), 0.96 (dd, *J* = 12.4, 3.2 Hz, H-9), 0.99 (m, H′-22), 1.00 (m, H′-1), 1.17 (d, *J* = 9.5 Hz, H-14), 1.22 (m, H′-12), 1.23 (m, H-5), 1.31 (m, H′-23), 1.33 (m, H-17), 1.33 (m, H-20), 1.42 (s, H_3_-19), 1.47 (m, H′-11), 1.58 (m, H-22), 1.58 (m, H-23), 1.59 (dd, *J* = 15.0, 3.2 Hz, H′-7), 1.61 (m, H′-2), 1.72 (m, H-1), 1.72 (m, H′-16), 1.80 (m, H-11), 1.83 (m, H-25), 1.88 (m, H-2), 1.90 (m, H-16), 1.96 (m, H-12), 2.40 (dd, *J* = 15.0, 3.0 Hz, H-7), 3.30 (m, H-24), 3.50 (m, H-3), 4.05 (m, H-4), 4.24 (m, H-6), 4.27 (td, *J* = 9.5, 3.2 Hz, H-15), 4.91 (d, *J* = 1.7 Hz, H-1′), 3.96 (m, H-2′), 3.83 (dd, *J* = 6.6, 4.0 Hz, H-3′), 3.97 (m, H-4′), 3.74 (dd, *J* = 12.0, 3.0 Hz, H-5′), 3.63 (dd, *J* = 12.0, 5.2 Hz, H-5′); ^13^C-NMR (CD_3_OD, 176.04 MHz): 41.1 (C-1), 26.6 (C-2), 73.1 (C-3), 77.5 (C-4), 50.7 (C-5), 76.2 (C-6), 45.3 (C-7), 76.8 (C-8), 57.7 (C-9), 36.8 (C-10), 19.3 (C-11), 42.7 (C-12), 45.5 (C-13), 66.6 (C-14), 70.1 (C-15), 41.7 (C-16), 55.9 (C-17), 15.3 (C-18), 18.7 (C-19), 36.3 (C-20), 19.0 (C-21), 32.8 (C-22), 28.8 (C-23), 84.8 (C-24), 31.8 (C-25), 18.4 (C-26), 18.3 (C-27), 109.5 (C-1′), 83.9 (C-2′), 78.7 (C-3′), 85.1 (C-4′), 62.9 (C-5′).

### 3.5. Acid Hydrolysis of Compound ***1*** and Determination of Absolute Configurations of the Sugars by GC

Compound **1** (1.2 mg) in a solution of 2 M TFA (1.0 mL) was heated in a H_2_O bath at 100 °C for 2 h. The reaction mixture was diluted with H_2_O (0.5 mL), washed with CHCl_3_ (3 × 0.5 mL), and then evaporated under reduced pressure. (*R*)-(−)-2-octanol (Aldrich) (0.4 mL) and one drop of conc. TFA was added to the dried residue, and the reaction mixture was heated in a glycerol bath at 130 °C for 6 h. The solution was concentrated under reduced pressure and treated with a mixture of Py/Ac_2_O (1:1, 0.5 mL) for 24 h at room temperature. The reaction mixture was evaporated under reduced pressure, and the resulting acetylated 2-octylglycosides of monosaccharides were analyzed by GC using the corresponding standard samples prepared in the same manner. The retention times of four tautomeric forms (two pyranoses and two furanoses) for each monosaccharide derivative from **1** were as follows: 2,4-di-*O*-methyl-d-xylose (19.96, 20.04 min); L-arabinose (22.44, 22.79, 23.04, and 23.33 min); and D-glucose (26.31, 26.95, 27.18, and 27.46 min). The retention times of the standard samples were as follows: 2,4-di-*O*-methyl-d-xylose (19.95, 20.03 min); L-arabinose (22.48, 22.83, 23.08, and 23.37 min); and D-glucose (26.32, 26.97, 27.19, and 27.48 min).

### 3.6. Solvolysis of Compound ***2***

A solution of **2** (2.8 mg) in a mixture of dioxane/Py (1:1, 0.8 mL) was heated at 100 °C for 4 h. The reaction mixture was evaporated under reduced pressure and purified by HPLC on an analytical Diasfer-110-C18 column with 80% aq. MeOH (0.5 mL/min) as an eluent system to give pure desulfated derivative **2a** (1.2 mg): (−)HRESIMS *m*/*z* 759.4547 [M − H]^−^ (calcd for C_39_H_67_O_14_, 759.4536), 819.4754 [M + C_2_H_4_O_2_]^−^ (calcd for C_41_H_72_O_16_, 819.4748); (+)HRESIMS *m*/*z* 783.4535 [M + Na]^+^ (calcd for C_39_H_68_O_14_Na, 783.4501); (−)ESIMS/MS of the [M − H]^−^ ion with *m*/*z* 759: 627 [(M − H) − C_5_H_8_O_4_]^−^, 609 [(M − H) − C_5_H_10_O_5_], 467 [(M − H) − C_5_H_8_O_4_ − C_6_H_10_O_4_]^−^, 131 [C_5_H_7_O_4_]^−^; (+)ESIMS/MS of the [M + Na]^+^ ion with *m*/*z* 783: 765 [(M + Na) − H_2_O]^+^, 623 [(M + Na) − C_6_H_10_O_4_]^+^; ^1^H-NMR and ^13^C-NMR data (Figures S18 and S19) were identical with those of forbeside J [22]: ^1^H-NMR (CD_3_OD, 700.13 MHz): *δ*_H_ 0.90 (d, *J* = 6.8 Hz, H_3_-26), 0.90 (d, *J* = 6.8 Hz, H_3_-27), 0.91 (d, *J* = 6.8 Hz, H_3_-21), 0.95 (s, H_3_-18), 0.97 (dd, *J* = 12.5, 3.0 Hz, H-9), 0.99 (m, H′-22), 1.01 (m, H′-1), 1.17 (m, H-14), 1.22 (m, H′-12), 1.23 (m, H-5), 1.31 (m, H′-23), 1.33 (m, H-17), 1.34 (m, H-20), 1.43 (s, H_3_-19), 1.47 (m, H′-11), 1.59 (m, H-22), 1.59 (m, H-23), 1.59 (m, H′-7), 1.70 (m, H′-2), 1.72 (m, H′-16), 1.74 (m, H-1), 1.81 (m, H-11), 1.83 (m, H-25), 1.90 (m, H-16), 1.96 (m, H-2), 1.97 (m, H-12), 2.41 (dd, *J* = 15.0, 2.9 Hz, H-7), 3.31 (m, H-24), 3.64 (m, H-3), 4.25 (m, H-4), 4.26 (m, H-6), 4.27 (td, *J* = 10.2, 3.6 Hz, H-15), 4.92 (d, *J* = 1.8 Hz, H-1′), 3.96 (m, H-2′), 3.83 (dd, *J* = 6.6, 4.0 Hz, H-3′), 3.97 (m, H-4′), 3.74 (dd, *J* = 11.9, 3.3 Hz, H-5′), 3.63 (m, H-5′), 4.44 (d, *J* = 7.5 Hz, H-1″), 2.92 (dd, *J* = 9.1, 7.6 Hz, H-2″), 3.43 (t, *J* = 8.9 Hz, H-3″), 3.17 (m, H-4″), 3.14 (dd, *J* = 11.0, 10.0 Hz, H-5″), 4.00 (dd, *J* = 11.0, 4.8 Hz, H-5″), 3.46 (s, 2″-OMe), 3.61 (s, 4″-OMe); ^13^C-NMR (CD_3_OD, 176.04 MHz): 41.1 (C-1), 25.2 (C-2), 80.5 (C-3), 74.6 (C-4), 50.5 (C-5), 76.2 (C-6), 45.2 (C-7), 76.7 (C-8), 57.7 (C-9), 36.9 (C-10), 19.3 (C-11), 42.7 (C-12), 45.5 (C-13), 66.5 (C-14), 70.1 (C-15), 41.7 (C-16), 55.9 (C-17), 15.3 (C-18), 18.6 (C-19), 36.3 (C-20), 19.0 (C-21), 32.8 (C-22), 28.8 (C-23), 84.8 (C-24), 31.8 (C-25), 18.4 (C-26), 18.3 (C-27), 109.5 (C-1′), 83.9 (C-2′), 78.7 (C-3′), 85.1 (C-4′), 62.9 (C-5′), 102.5 (C″-1), 84.7 (C″-2), 76.8 (C″-3), 81.0 (C″-4), 64.2 (C″-5), 59.0 (2″-OMe), 61.0 (4″-OMe).

### 3.7. Reagents

The phosphate-buffered saline (PBS), L-glutamine, penicillin/streptomycin solution (10,000 U/mL, 10 µg/mL), Minimum Essential Medium Eagle (MEM), Dulbecco’s Modified Eagle Medium (DMEM), McCoy’s 5A modified medium (McCoy’s 5A), and Basal Medium Eagle (BME) were purchased from the Sigma-Aldrich company (St. Louis, MO, USA). The MTS reagent 3-[4,5-dimethylthiazol-2-yl]-2,5-diphenyltetrazolium bromide was purchased from Promega (Madison, WI, USA). The trypsin, fetal bovine serum (FBS), and the protein marker PageRulerTM Plus Prestained Protein Ladder were purchased from Thermo Fisher Scientific (Waltham, MA, USA).

The cell lysis buffer (10×), CDK2 (#2546s), CDK4 (#12790s), Cyclin D1 (#2922s), p21 Waf1/Cip1 (#2947s), phospho-c-Raf (Ser338) (#9427), phospho-p44/42 MAPK (phospho-Erk1/2) (Thr202/Tyr204) (#9101), p44/42 MAPK (Erk1/2) (#9102), phospho-MEK1/2 (Ser217/221) (#9121), and MEK1/2 (#9122) antibodies were obtained from Cell Signaling Technology (Danvers, MA, USA); β-actin and horseradish peroxidase (HRP) conjugated secondary antibody from rabbit and mouse were purchased from the Sigma-Aldrich company (St. Louis, MO, USA).

### 3.8. Cell Lines and Cell Culture Conditions

Human cell lines, including embryonic kidney HEK293 (ATCC^®^ CRL-1573™), melanoma SK-MEL-28 (ATCC^®^ HTB-72™), breast cancer MDA-MB-231 (ATCC^®^ HTB-26™), and colorectal carcinoma cells HCT 116 (ATCC^®^ CCL-247™) were obtained from American Type Culture Collection (ATCC, Manassas, VA, USA). All cells were maintained according to ATCC protocols and routinely checked for contamination with mycoplasma.

HEK293 cells were grown in MEM medium; SK-MEL-28 and MDA-MB-231 cells were cultured in DMEM medium, while HCT 116 cells were maintained in McCoys’ 5A medium according to the manufacturer’s instructions. Culture media were supplemented with 10% heat-inactivated fetal bovine serum (FBS) and 1% penicillin–streptomycin solution. The cells were cultured at 37 °C in a humidified atmosphere containing 5% CO_2_.

### 3.9. Cell Viability Assay

The CellTiter 96^®^ Aqueous One Solution Cell Proliferation Assay kit (MTS) was used for cell viability analysis and performed according to the standard protocol. Briefly, cells (1 × 10^4^/200 µL) were seeded in 96-well plates and incubated for 24 h in a humidified atmosphere containing 5% CO_2_. Then, they were treated with DMSO (control) and compounds T1, B1, and B2 at 1, 10, 50, and 100 µM for an additional 24 h. The MTS reagent (20 μL/well) was added to the cell culture medium and incubated at a 37 °C incubator for 2 h. Cell viability was examined at 490/630 nm using a Power Wave XS microplate reader (BioTek, Winooski, VT, USA).

The concentration at which the compounds exert half of their maximal inhibitory effect on cell viability (IC_50_) was calculated by the AAT-Bioquest^®^ online calculator [39]. The selectivity index (SI) was calculated as described previously [40] using the following formula: SI = IC_50_ of the compounds in normal cell (HEK293)/IC_50_ of the same compounds in human colorectal adenocarcinoma cell line (HCT 116).

### 3.10. Cell Proliferation Assay

HCT 116 cells (8 × 10^3^/200 µL) were seeded in 96-well plates and incubated for 24 h in a CO_2_ incubator. The cells’ monolayers were washed with phosphate-buffered saline (PBS) to remove unattached cells. The attached cells were incubated with fresh medium containing DMSO (control) and B2 (0–100 µM) for 24, 48, and 72 h. Subsequently, the cells were incubated with 15 µL MTS reagent for 2 h, and the absorbance of each well was measured at 490/630 nm using a microplate reader (Power Wave XS, USA).

### 3.11. Anchorage-Independent Cell Growth Assay

Soft agar assay was performed as described previously [41]. Briefly, the cells were counted and seeded into 6-well plates at a density of 8×10^3^/per well with 0.3% BME agar containing 10% FBS and DMSO (control) or various concentrations of compounds **1**, **2**, and **3** (10, 20, and 40 µM). The number of the colonies was determined using a Motic microscope AE 20 and ImageJ software bundled with 64-bit Java 1.8.0_112 (NIH, Bethesda, MD, USA) 14 days later.

### 3.12. Cell Cycle Assay

HCT 116 cells (3 × 10^5^) were seeded in 60 mm dishes and incubated for 24 h in a CO_2_ incubator. The attached cells were treated by DMSO (control) or **3** (10, 20, and 40 µM) for 72 h. Then, cells were harvested, washed with ice-cold 1× PBS, and fixed with 70% ethanol. HCT 116 cells were incubated overnight at –20 °C, and then fixed cells were collected by centrifugation at 4000 rpm for 10 min and rinsed with 1× PBS. The cell pellet was resuspended in Muse™ Cell Cycle Reagent (MCH100106, Luminex, Austin, TX, USA), and the cells were incubated for 30 min at RT in the dark. The DNA content was assessed by measuring the fluorescence intensity by flow cytometry (Muse™ Cell Analyzer). Results were expressed as a percentage of cells in the G0/1, S, and G2/M phases of the cell cycle, associated with the DNA content profile histograms

### 3.13. Western Blot Assay

HCT 116 cells (1.0 × 10^5^/mL) were seeded in 100 mm dishes and incubated for 24 h at 37 °C in a CO_2_ incubator. The cells were treated by DMSO (control) or **3** (10, 20, and 40 µM) for 72 h. Then, cells were harvested and lysed by 1× cell lysis buffer (“Cell Signaling Technology”, Danvers, MA, USA) according to the manufacturer’s protocol. Cells’ protein content was determined by the DC protein assay (Bio-Rad, Hercules, CA, USA). Lysates of protein (20–40 µg) were exposed to 10% or 12% SDS-PAGE and electrophoretically transferred to polyvinylidene difluoride membranes (PVDF) (Millipore, Burlington, MA, USA). The membranes were blocked with 5% non-fat milk (Bio-Rad) for 1 h and then incubated with the respective specific primary antibody at 4 °C overnight. Protein bands were visualized using an enhanced chemiluminescence reagent (ECL) (Bio-Rad, Hercules, CA, USA) after hybridization with an HRP-conjugated secondary antibody.

### 3.14. Statistical Analysis

All of the assays were performed in at least three independent experiments. Results are expressed as the mean ±standard deviation (SD). Statistical procedures were performed using one-way ANOVA and Tukey’s HSD tests with * *p* < 0.05, ** *p* < 0.01, and *** *p* < 0.001.

## 4. Conclusions

Polar steroid compounds from the ethanolic extract of the Sea of Okhotsk starfish *Henricia leviuscula spiculifera* were investigated. New monosulfated steroid glycosides, spiculiferosides A, B, and C, and a new unsulfated related monoglycoside, spiculiferoside D, were isolated, and their chemical structures were characterized. Three of them contain two carbohydrate chains, which are located at positions C-3 and C-24 of the polyhydroxylated cholestane aglycone. Spiculiferosides B and C are biosides, and spiculiferoside A, in contrast, has three monosaccharide residues. Previously, only five such “two-chains” triglycosides were known from sea stars, kurilensosides A, B, C, and I, found in the Far Eastern starfish *Hippasteria kurilensis* [27,32], and planciside D isolated from the tropical starfish *Acanthaster planci* [33], which also contain two carbohydrate patterns attached to the steroid core and aglycon side chain. The 5-substituted 3-OSO_3_-α-L-Araf residue of spiculiferoside A was discovered and described for the first time in steroid glycosides of starfish. Moreover, the 3-OSO_3_-α-L-Araf residue that comprised spiculiferosides B and C, was previously found only in one steroid glycoside from the sea star *Oreaster reticulatus* [34]. Interestingly, we did not find “classical” oligoglycosides (asterosaponins) in the starfish *H. leviuscula spiculifera* as in most previously studied species of the genus *Henricia*.

Spiculiferosides A, B, and C exhibited moderate cytotoxic activities against human embryonic kidney HEK293, melanoma SK-MEL-28, breast cancer MDA-MB-231, and colorectal carcinoma HCT 116 cell lines but significantly inhibited proliferation and colony formation in HCT 116 cells. Spiculiferoside C demonstrated the highest anti-cancer activity among the investigated compounds. The molecular mechanism of anti-cancer action of this compound was associated with the induction of cell cycle arrest at the G2/M phase through regulation of CDK2, CDK4, cyclin D1, and p21WAF1/CIP1 proteins’ expression and inhibition of mitogen-activated protein kinases of MAPK/ERK1/2 signaling cascade.

## Figures and Tables

**Table 1:** ^1^H- and ^13^C-NMR spectroscopic data of aglycon moieties of **1**–**3** in CD_3_OD *^a^*.

Position	1, 2 *^b^*		3	
	*δ*_H_ (*J* in Hz)	*δ*_C_	*δ*_H_ (*J* in Hz)	*δ*_C_
1	1.73 m 1.01 m	41.0, CH_2_	1.65 m 1.00 m	39.4, CH_2_
2	1.96 m 1.70 m	25.3, CH_2_	1.93 m 1.68 m	25.4, CH_2_
3	3.64 m	80.6, CH	3.58 m	81.0, CH
4	4.25 m	74.7, CH	4.23 m	74.7, CH
5	1.23 m	50.5, CH	1.12 m	50.3, CH
6	4.25 m	76.2, CH	4.15 m	74.7, CH
7	2.39 dd (15.0, 2.8) 1.60 dd (15.0, 3.1)	45.3, CH_2_	2.13 ddd (14.3, 6.8, 3.3) 1.29 m	40.5, CH_2_
8	–	76.8, C	1.99 m	31.8, CH
9	0.97 m	57.6, CH	0.72 td (11.0, 4.5)	56.1, CH
10	–	36.9, C	–	36.8, C
11	1.80 m 1.46 m	19.3, CH_2_	1.45 m 1.39 m	21.4, CH_2_
12	1.96 m 1.24 m	42.7, CH_2_	1.96 m 1.22 m	41.4, CH_2_
13	–	45.4, C	–	44.8, C
14	1.17 d (9.5)	66.4, CH	1.05 dd (10.9, 9.3)	63.6, CH
15	4.27 td (9.5, 3.1)	70.1, CH	3.88 td (9.3, 3.4)	74.3, CH
16	1.92 m 1.78 m	42.0, CH_2_	1.92 m 1.79 m	42.0, CH_2_
17	1.33 m	56.1, CH	1.39 m	55.1, CH
18	0.95 s	15.3, CH_3_	0.74 s	13.8, CH_3_
19	1.43 s	18.7, CH_3_	1.33 s	18.1, CH_3_
20	1.31 m	36.5, CH	1.34 m	37.0, CH
21	0.90 d (6.0)	19.0, CH_3_	0.93 d (6.3)	19.2, CH_3_
22	1.63 m 0.94 m	33.1, CH_2_	1.64 m 0.99 m	33.1, CH_2_
23	1.55 m 1.26 m	28.6, CH_2_	1.58 m 1.29 m	28.5, CH_2_
24	3.31 m	84.0, CH	3.34 m	84.0, CH
25	1.86 m	31.5, CH	1.86 m	31.5, CH
26	0.88 d (6.8)	18.5, CH_3_	0.89 d (6.9)	18.5, CH_3_
27	0.89 d (6.8)	18.1, CH_3_	0.89 d (6.9)	18.2, CH_3_

*^a^* Assignments from ^1^H 500.13 MHz, ^13^C 125.76 MHz, ^1^H-^1^H COSY, HSQC, HMBC (8 Hz), ROESY (250 ms), and DEPT 135 spectra. *^b^* Data were extracted from the ^1^H- and ^13^C-NMR spectra of compound **1**.

**Table 2:** ^1^H- and ^13^C-NMR spectroscopic data of carbohydrate moieties of **1**–**3** in CD_3_OD *^a^*.

Position	1		2, 3 *^b^*	
	*δ*_H_ (*J* in Hz)	*δ*_C_	*δ*_H_ (*J* in Hz)	*δ*_C_
**2,4-di-OMe-Xylp**				
1′	4.44 d (7.5)	102.6, CH	4.44 d (7.5)	102.7, CH
2′	2.92 dd (9.0, 7.5)	84.7, CH	2.91 dd (8.9, 7.5)	84.7, CH
3′	3.43 t (9.0)	76.7, CH	3.43 t (8.9)	76.6, CH
4′	3.17 m	81.0, CH	3.17 m	80.9, CH
5′	4.00 dd (10.0, 4.0) 3.13 t (10.0)	64.2, CH_2_	4.00 dd (10.1, 3.7) 3.14 t (10.1)	64.2, CH_2_
2′-OMe	3.61 s	61.0, CH_3_	3.61 s	61.0, CH_3_
4′-OMe	3.45 s	59.0, CH_3_	3.45 s	59.0, CH_3_
**3-OSO_3_** **^−^-Araf**				
1″	5.00 br s	109.2, CH	4.98 br s	109.5, CH
2″	4.24 m	82.0, CH	4.23 m	82.1, CH
3″	4.67 m	84.7, CH	4.46 dd (5.6, 2.3)	84.5, CH
4″	4.33 m	84.1, CH	4.22 m	84.4, CH
5″	4.14 dd (11.7, 3.7) 3.87 dd (11.7, 4.2)	69.6, CH_2_	3.83 dd (12.0, 3.0) 3.71 dd (12.0, 5.2)	63.1, CH_2_
**Glcp**				
1′″	4.34 d (7.7)	104.9, CH		
2′″	3.22 dd (9.0, 7.7)	75.1, CH		
3′″	3.35 t (9.0)	77.8, CH		
4′″	3.28 m	71.6, CH		
5′″	3.25 m	77.9, CH		
6′″	3.85 dd (12.1, 2.3) 3.66 dd (12.1, 5.3)	62.7, CH_2_		

*^a^* Assignments from ^1^H 500.13 MHz, ^13^C 125.76 MHz, ^1^H-^1^H COSY, HSQC, HMBC (8 Hz), ROESY (250 ms), and DEPT-135 spectra. *^b^* Data were extracted from the ^1^H- and ^13^C-NMR spectra of compound **2**.

## Data Availability

The data presented in this study are available on request from the corresponding authors.

## Supplementary Materials

The following supporting information can be downloaded at https://www.mdpi.com/article/10.3390/md22070294/s1. HRESIMS (Figures S1, S9, S17, S21 and S29), ^1^H-NMR (Figures S4, S10, S18, S22, and S30), ^13^C-NMR (Figures S2, S11, S19, S23 and S31), HSQC (Figures S6, S14, S20, S26, and S34) spectra of compounds **1**, **2**, **2a**, **3,** and **4**, resp. DEPT (Figures S3, S12, S24, and S32), ^1^H-^1^H COSY (Figures S5, S13, S25, and S33), HMBC (Figures S8, S15, S27, and S35), and ROESY (Figures S7, S16, S28, and S36) spectra of compounds **1**, **2**, **3,** and **4**, resp.
