# Supplementary material for: Sulfated Polyhydroxysteroid Glycosides from the Sea of Okhotsk Starfish Henricia leviuscula spiculifera and Potential Mechanisms for Their Observed Anti-Cancer Activity against Several Types of Human Cancer Cells
Source: Mar Drugs. 2024 Jun 26;22(7):294. doi: 10.3390/md22070294 (PMC11278266; doi:10.3390/md22070294)

Figure S1. HRESIMS and HRESIMS/MS spectra of compound **1**.

(–)HRESIMS: [M – Na]$^{-}$ ion

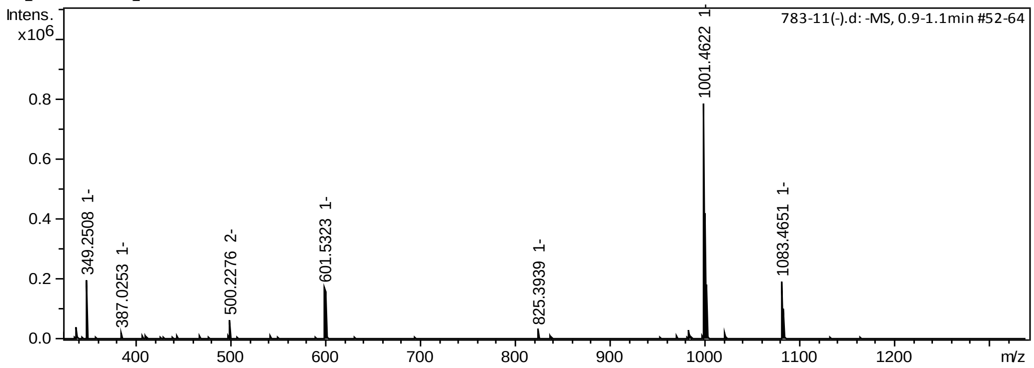

(+)HRESIMS: [M + Na]$^{+}$ ion

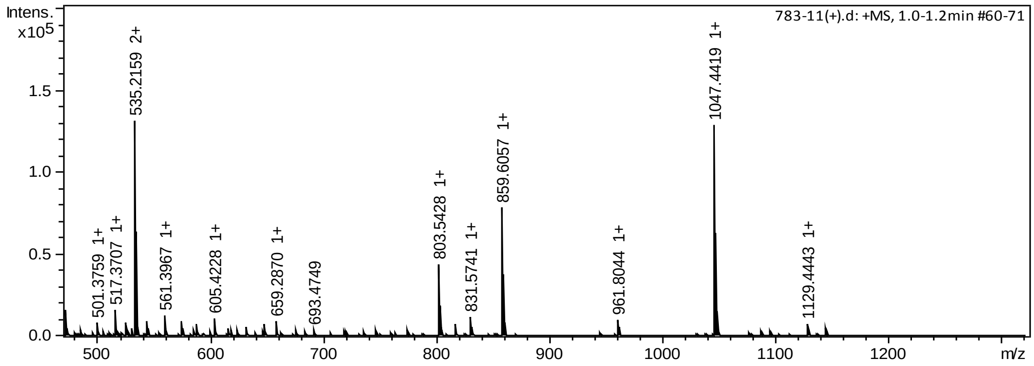

(–)HRESIMS/MS of the [M – Na]$^{-}$ ion at *m/z* 1001

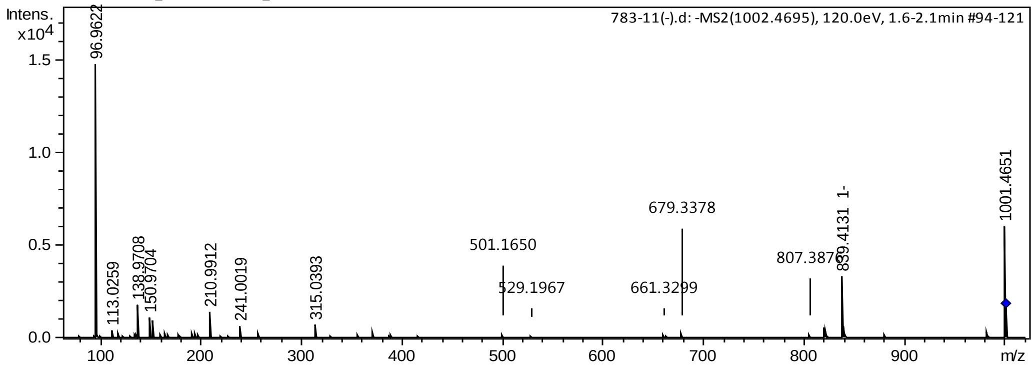

(+)HRESIMS/MS of the [M + Na]$^{+}$ ion at *m/z* 1047

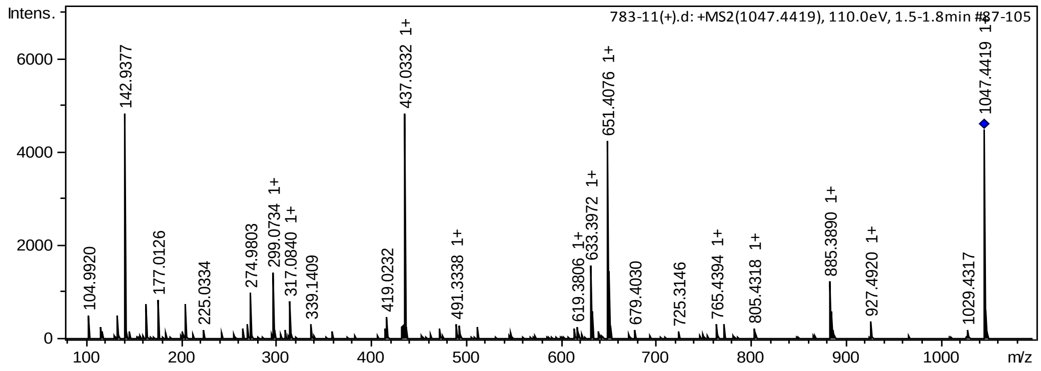

Figure S2. ^{13}C-NMR (125.76 MHz, CD_3OD) spectrum of compound **1**.

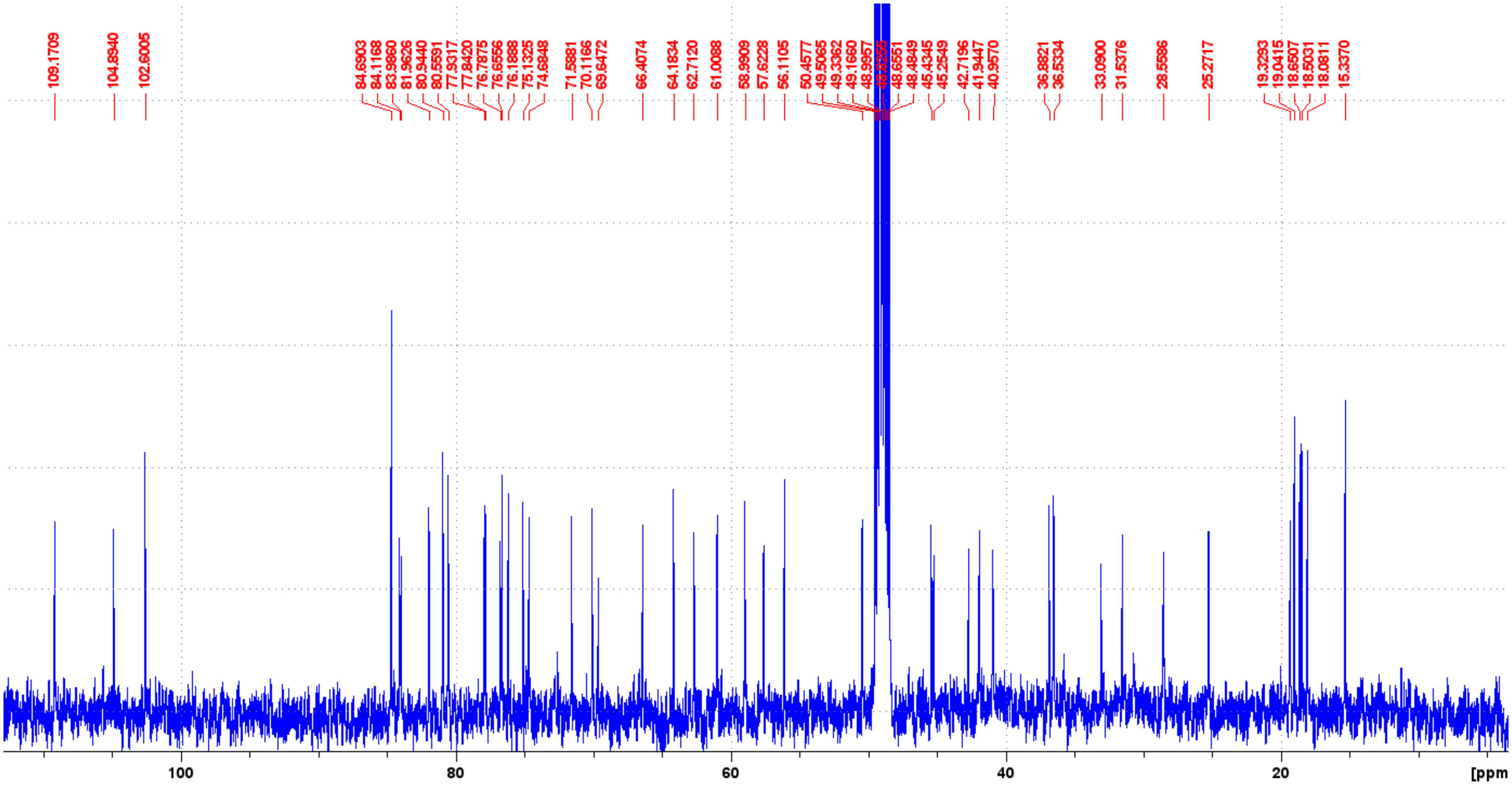

Figure S3. DEPT (125.76 MHz, CD_3OD) spectrum of compound **1**.

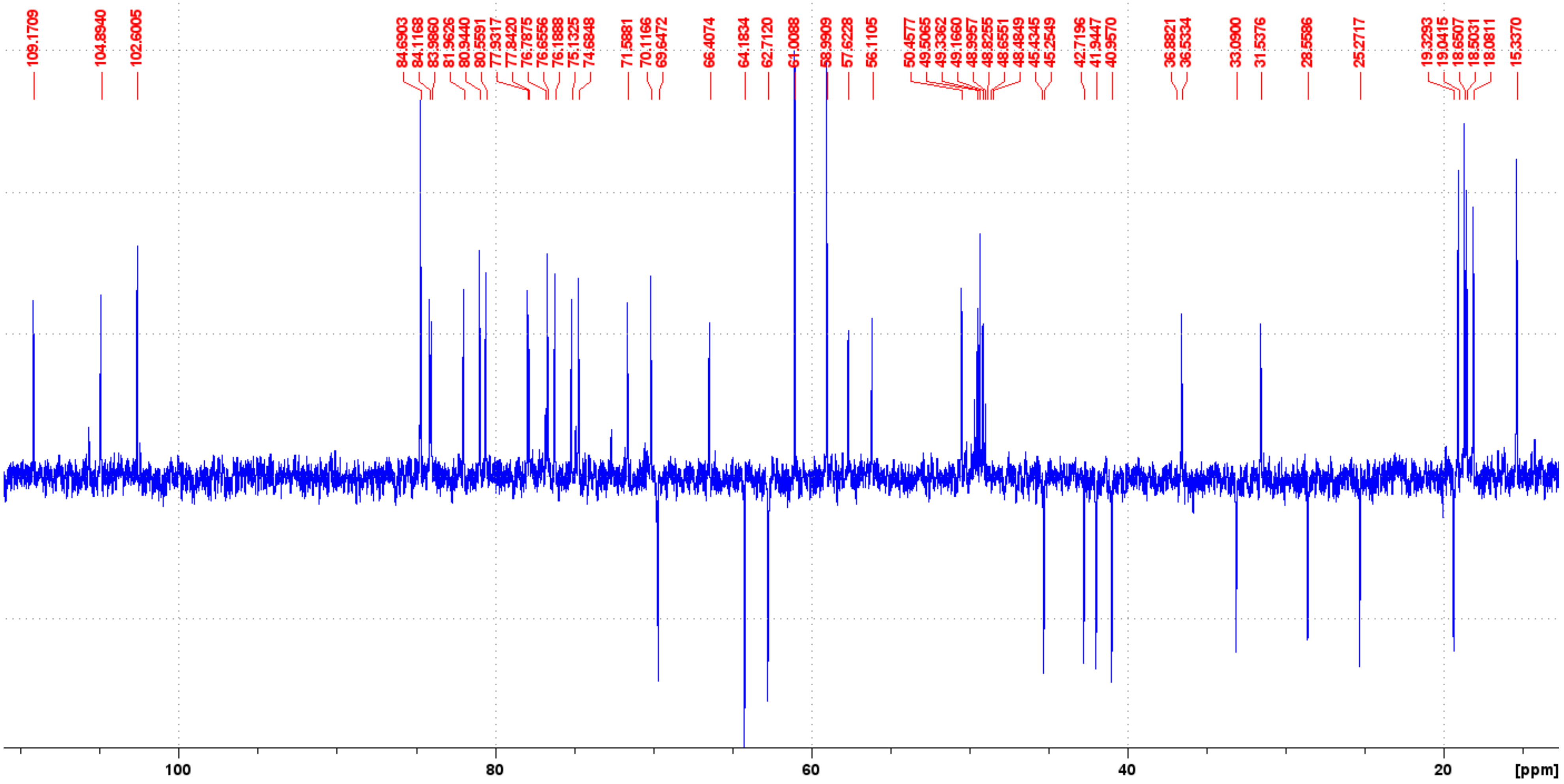

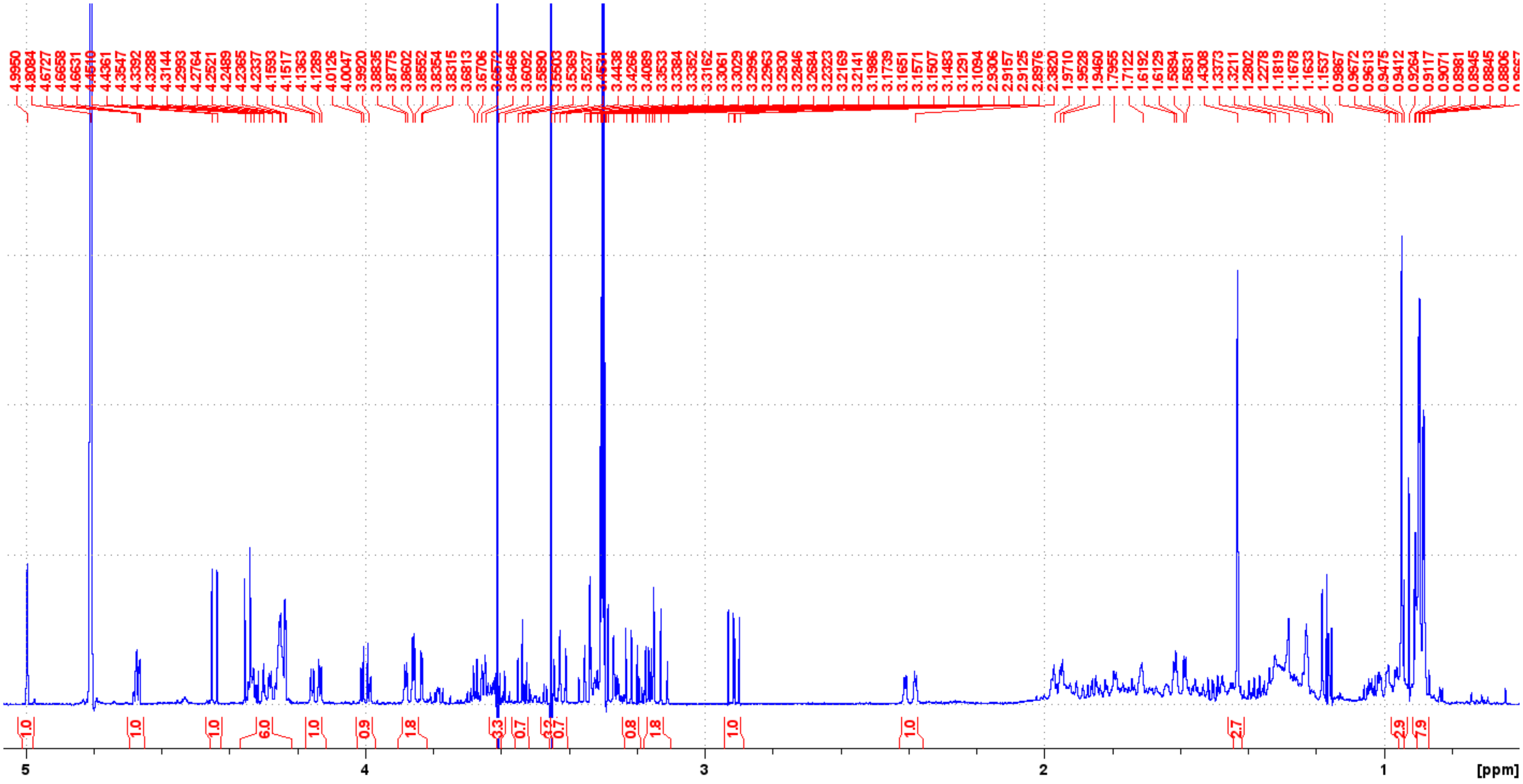

Figure S4. ^{1}H-NMR (500.13 MHz, CD_3OD) spectrum of compound **1**.

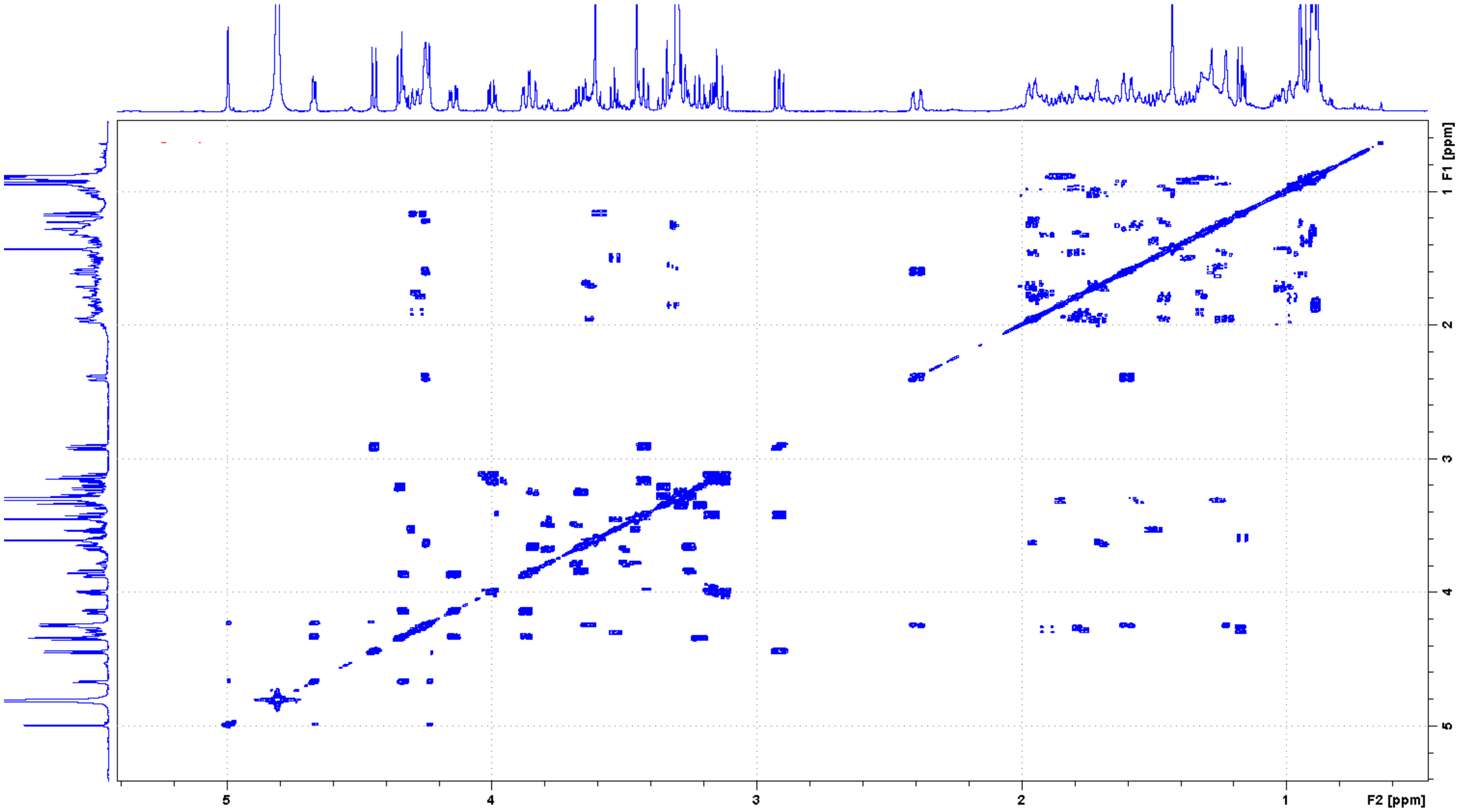

Figure S5. ^{1}H-^{1}H COSY (500.13 MHz, CD_3OD) spectrum of compound **1**.

Figure S6. HSQC (500.13 MHz, CD_3OD) spectrum of compound **1**.

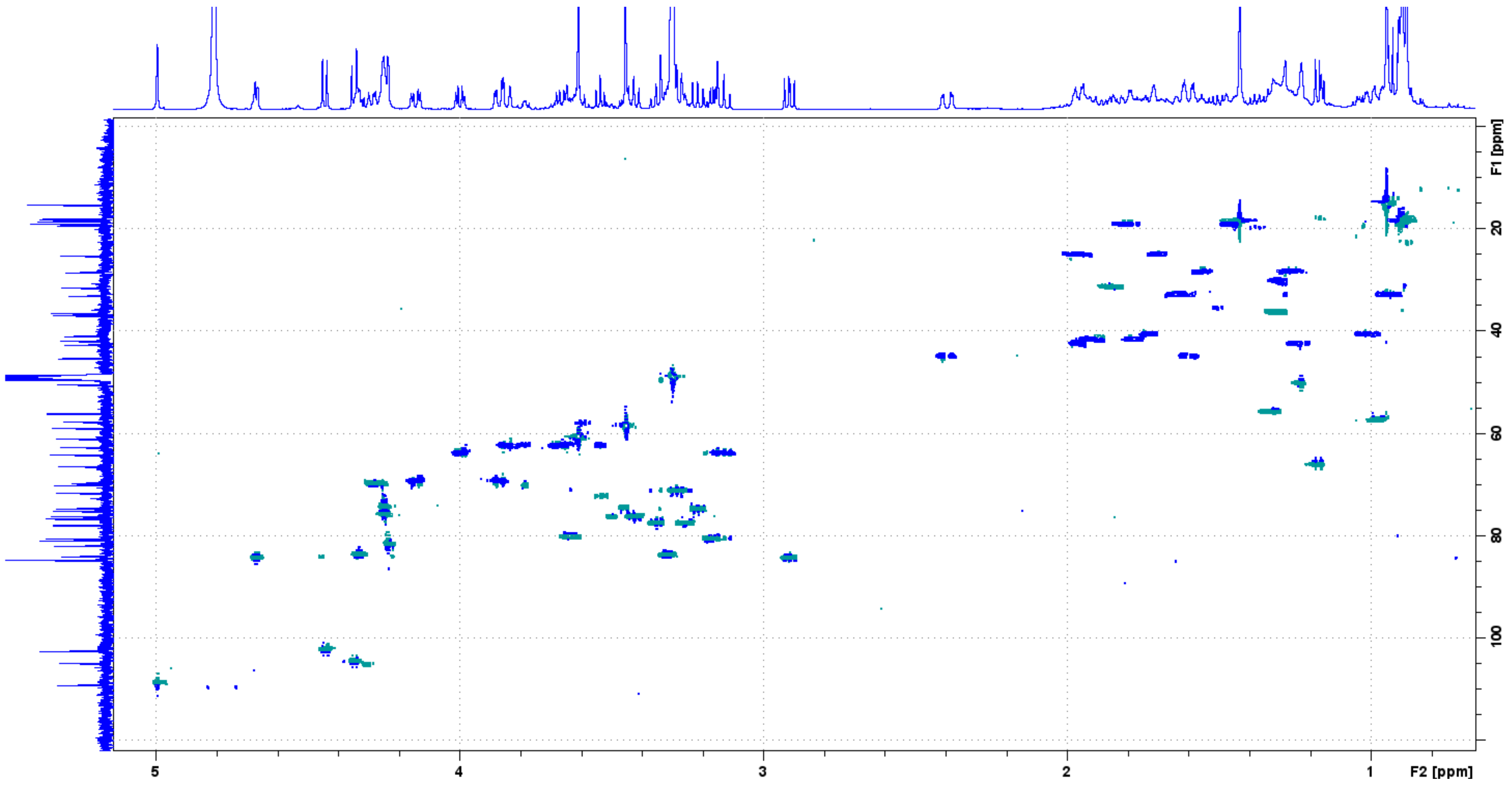

Figure S7. ROESY (500.13 MHz, CD_3OD) spectrum of compound **1**.

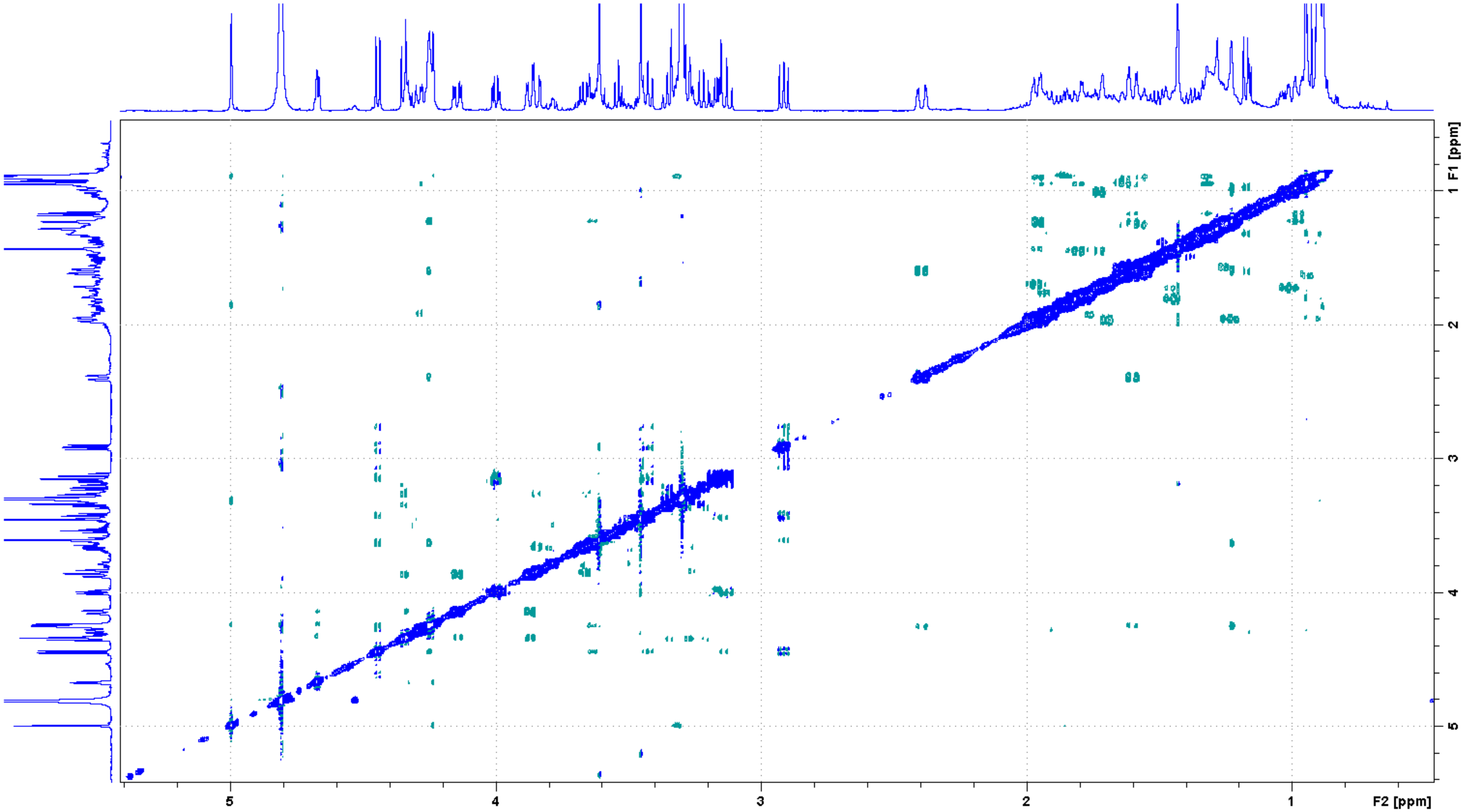

Figure S8. HMBC (500.13 MHz, CD_3OD) spectrum of compound **1**.

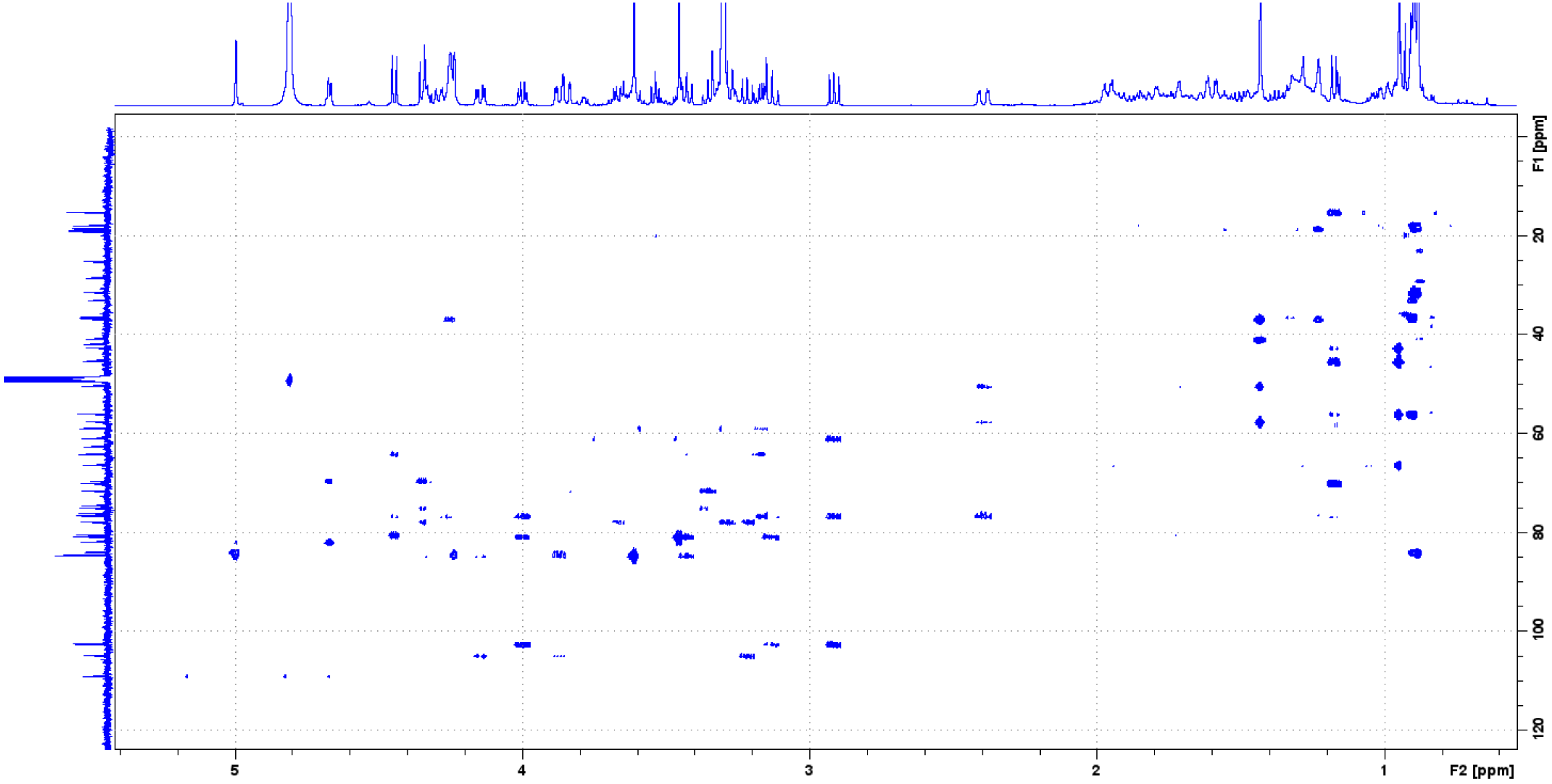

Figure S9. HRESIMS and HRESIMS/MS spectra of compound **2**.

(–)HRESIMS: [M – Na]$^-$ ion

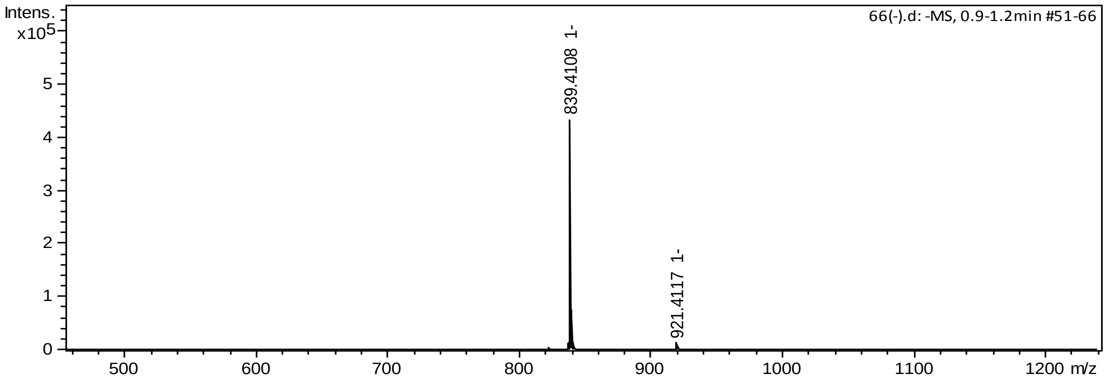

(+)HRESIMS: [M + Na]$^+$ ion

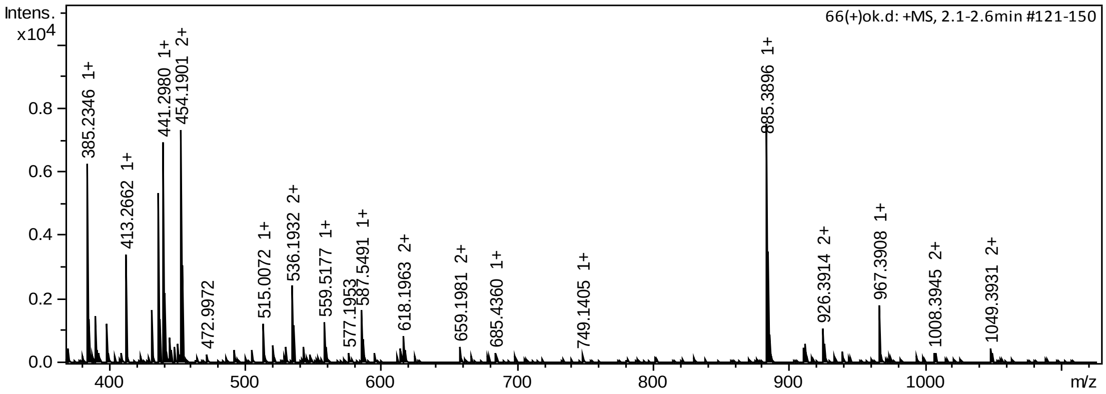

(−)HRESIMS/MS of the [M – Na]$^-$ ion at *m/z* 839

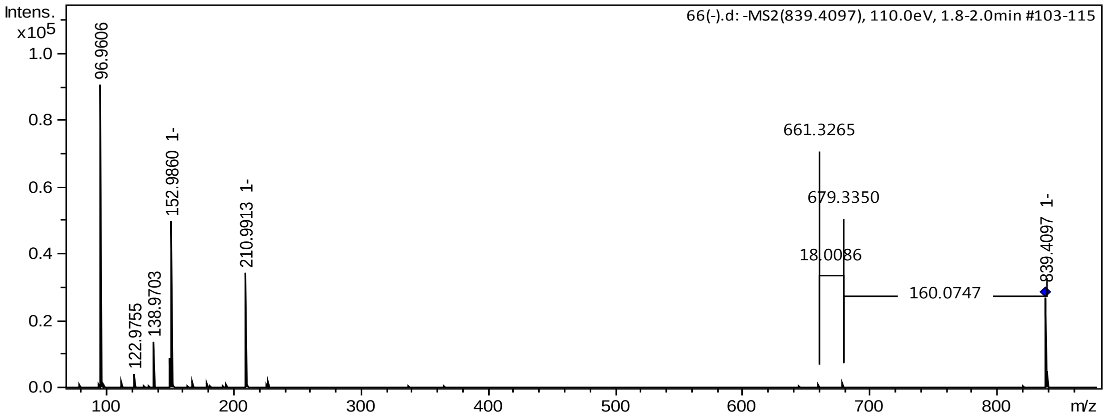

(+)HRESIMS/MS of the [M + Na]$^+$ ion at *m/z* 885

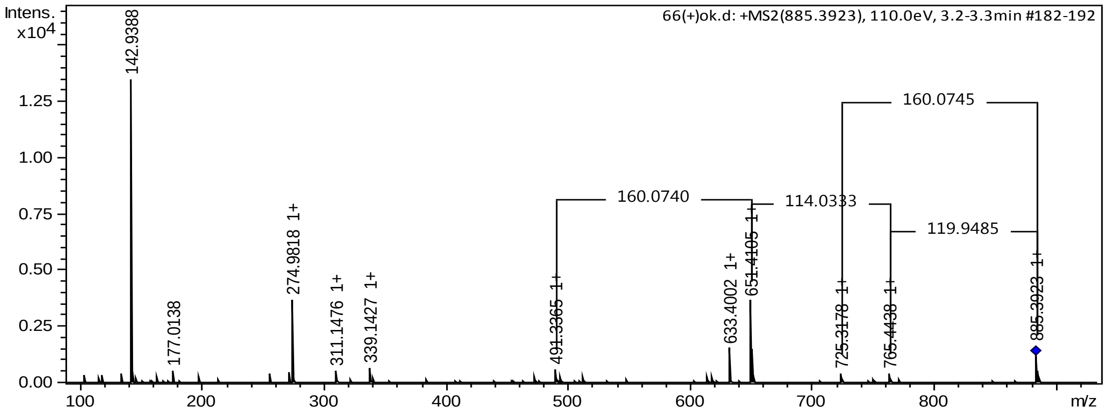

Figure S10. ^{1}H-NMR (500.13 MHz, CD_3OD) spectrum of compound **2**.

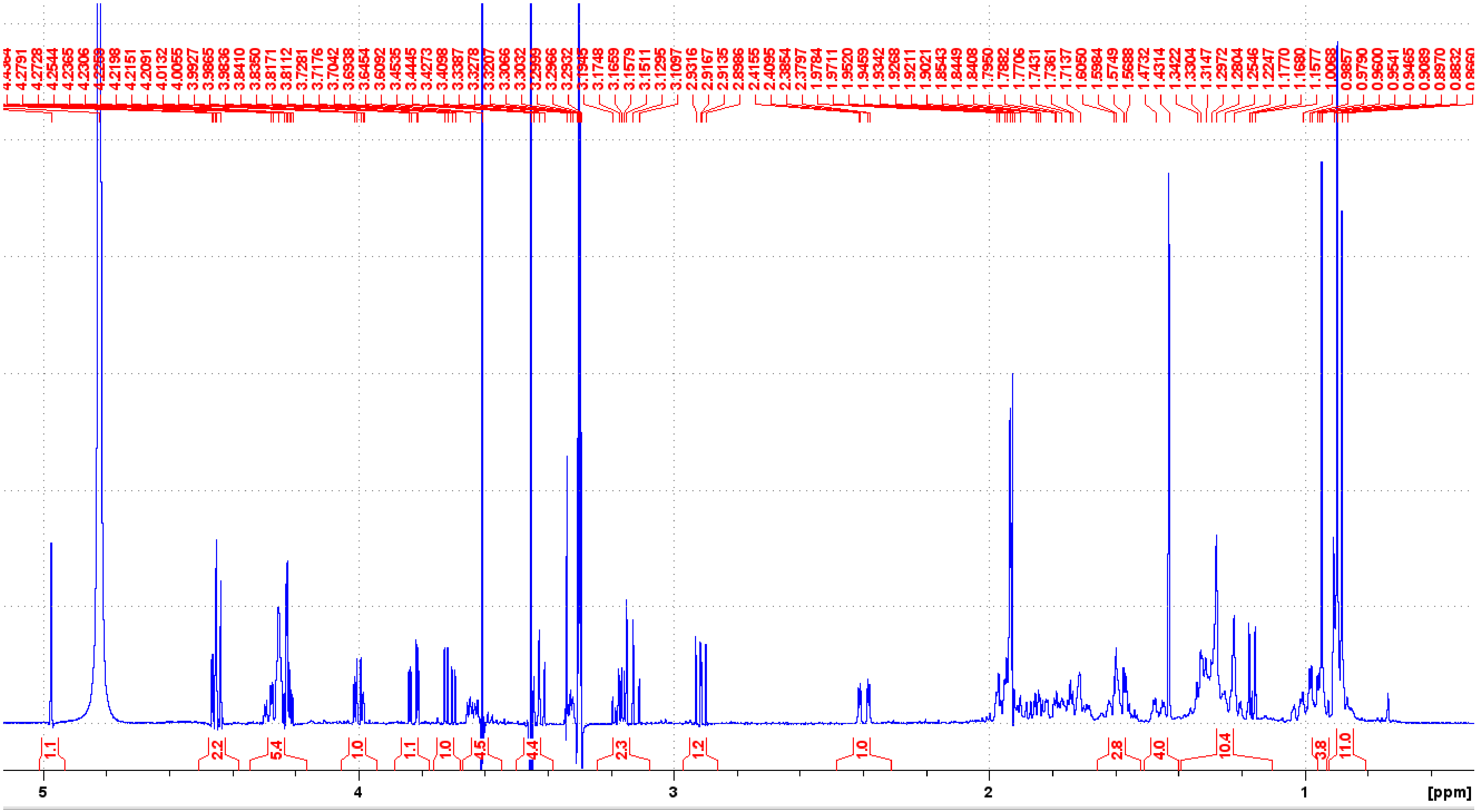

Figure S11. ^{13}C-NMR (125.76 MHz, CD_3OD) spectrum of compound **2**.

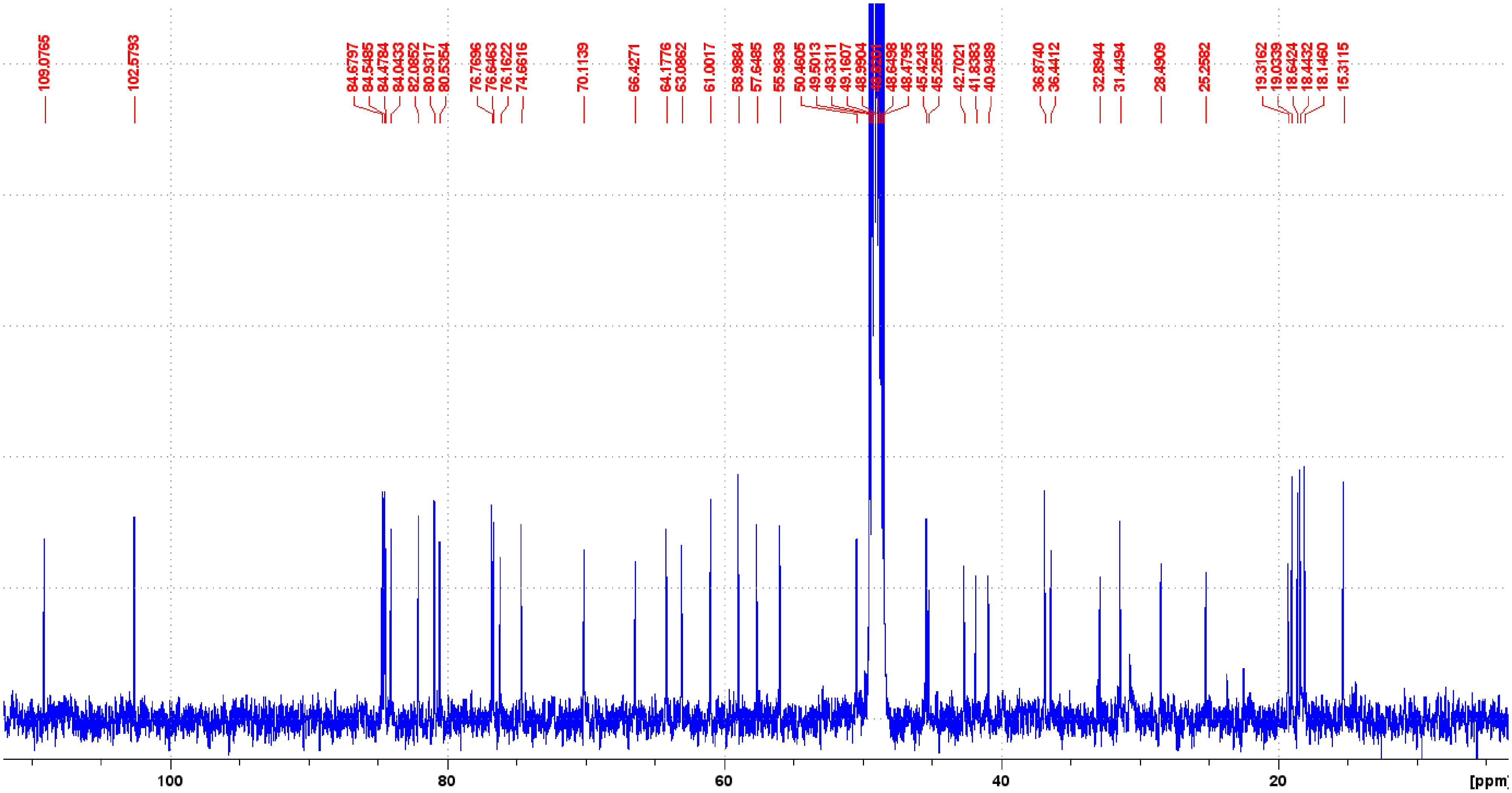

Figure S12. DEPT (125.76 MHz, CD_3OD) spectrum of compound **2**.

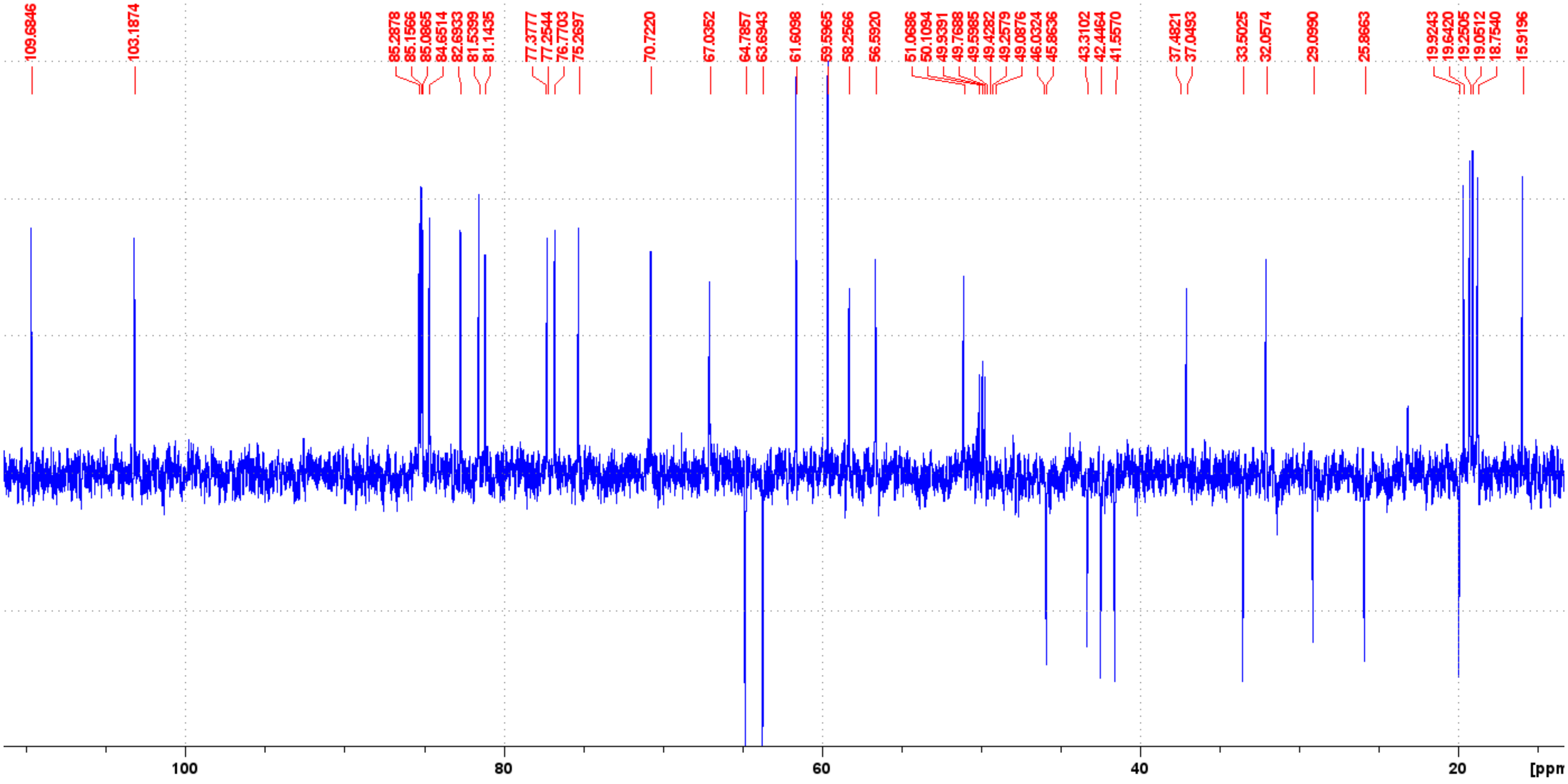

Figure S13. ^{1}H-^{1}H COSY (500.13 MHz, CD_3OD) spectrum of compound **2**.

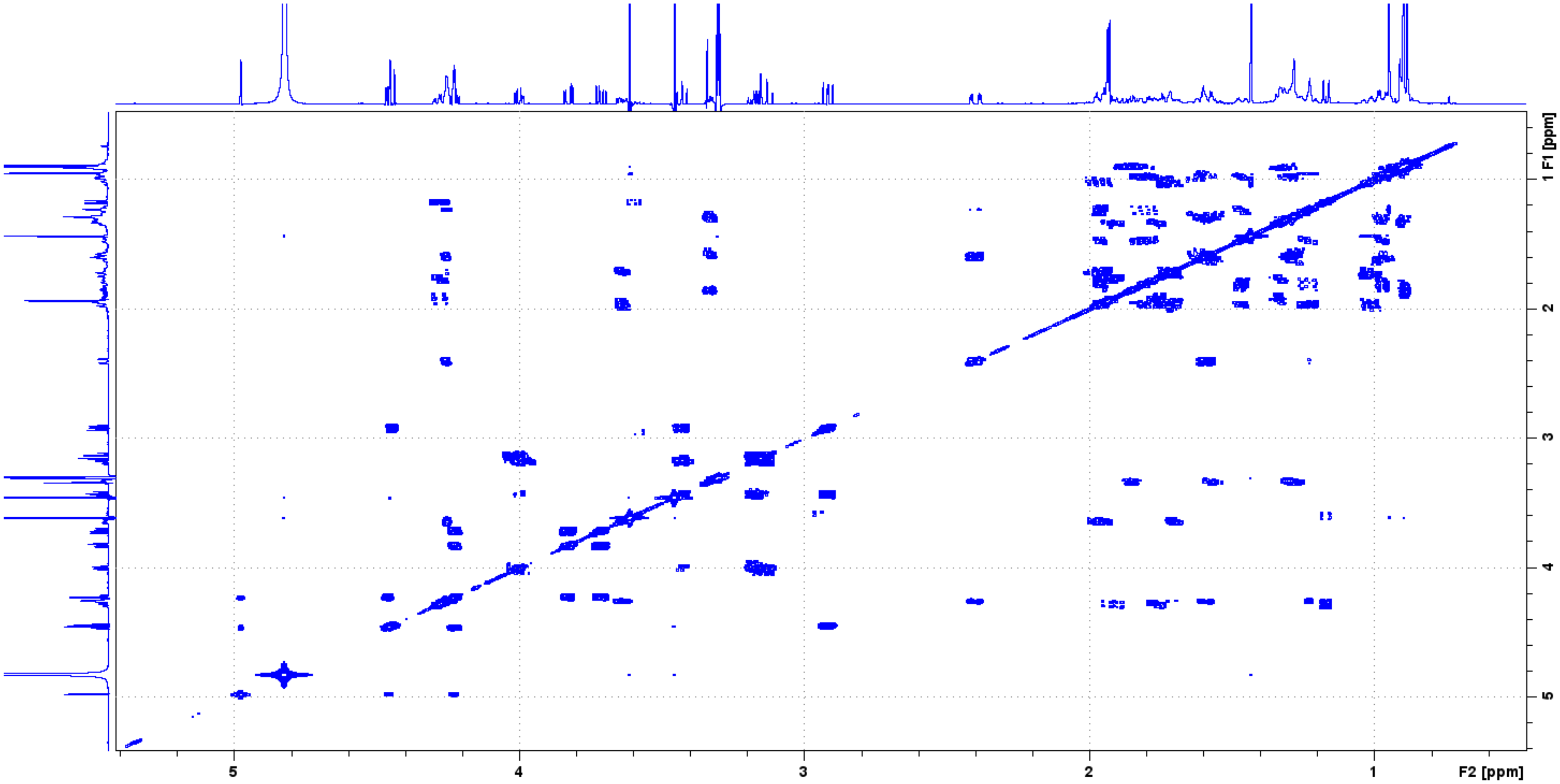

Figure S14. HSQC (500.13 MHz, CD_3OD) spectrum of compound **2**.

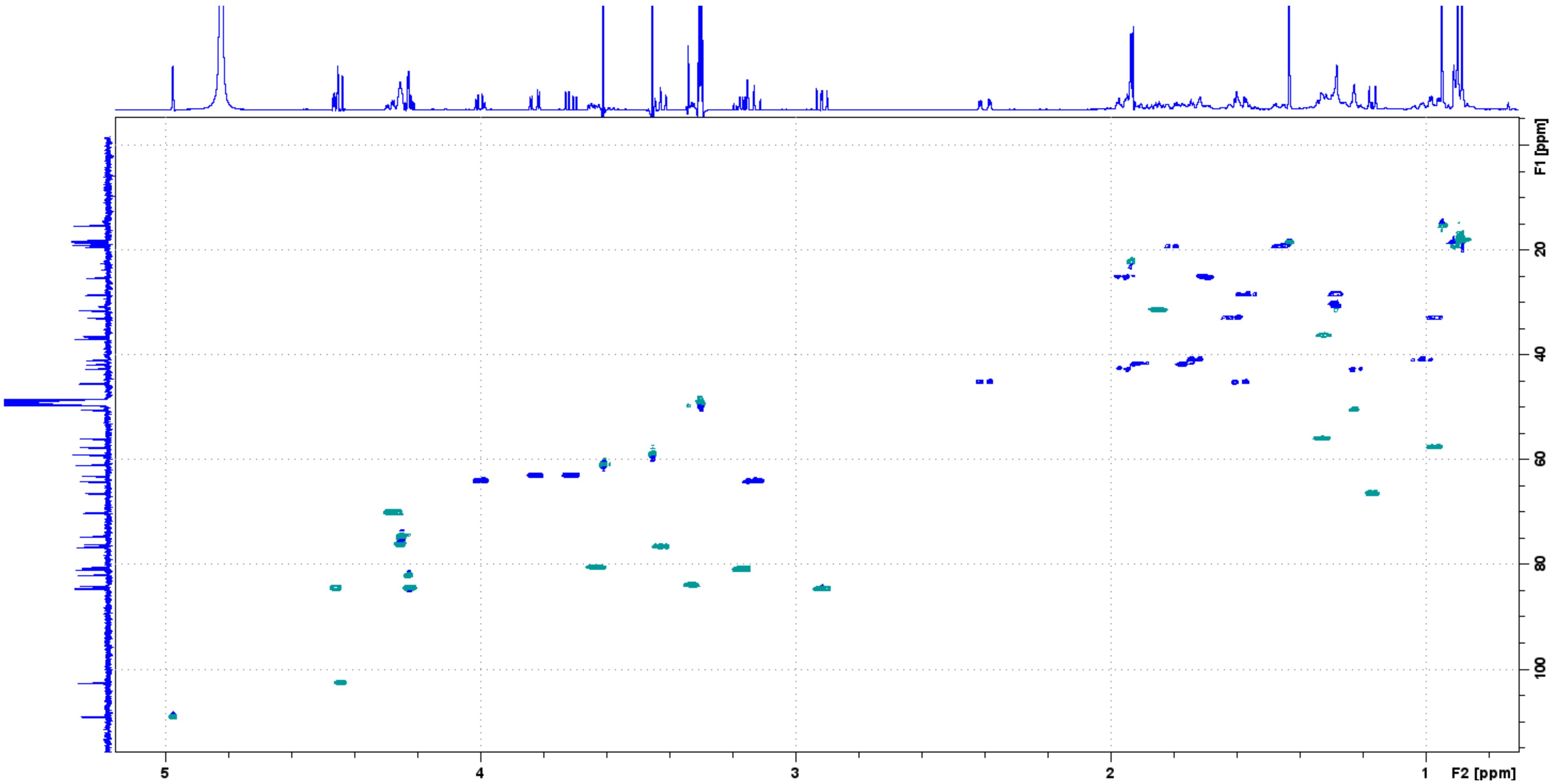

Figure S15. HMBC (500.13 MHz, CD_3OD) spectrum of compound **2**.

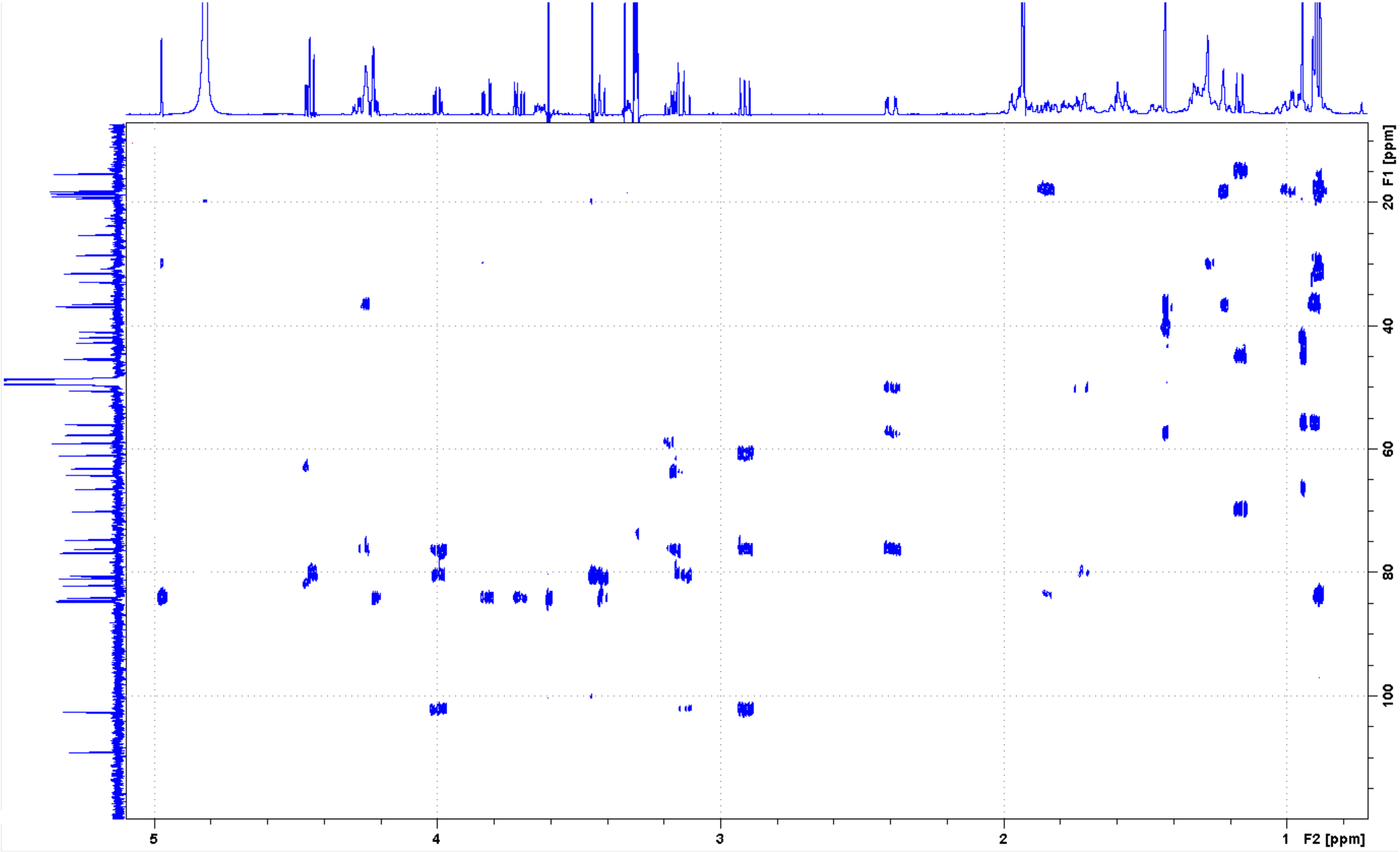

Figure S16. ROESY (500.13 MHz, CD_3OD) spectrum of compound **2**.

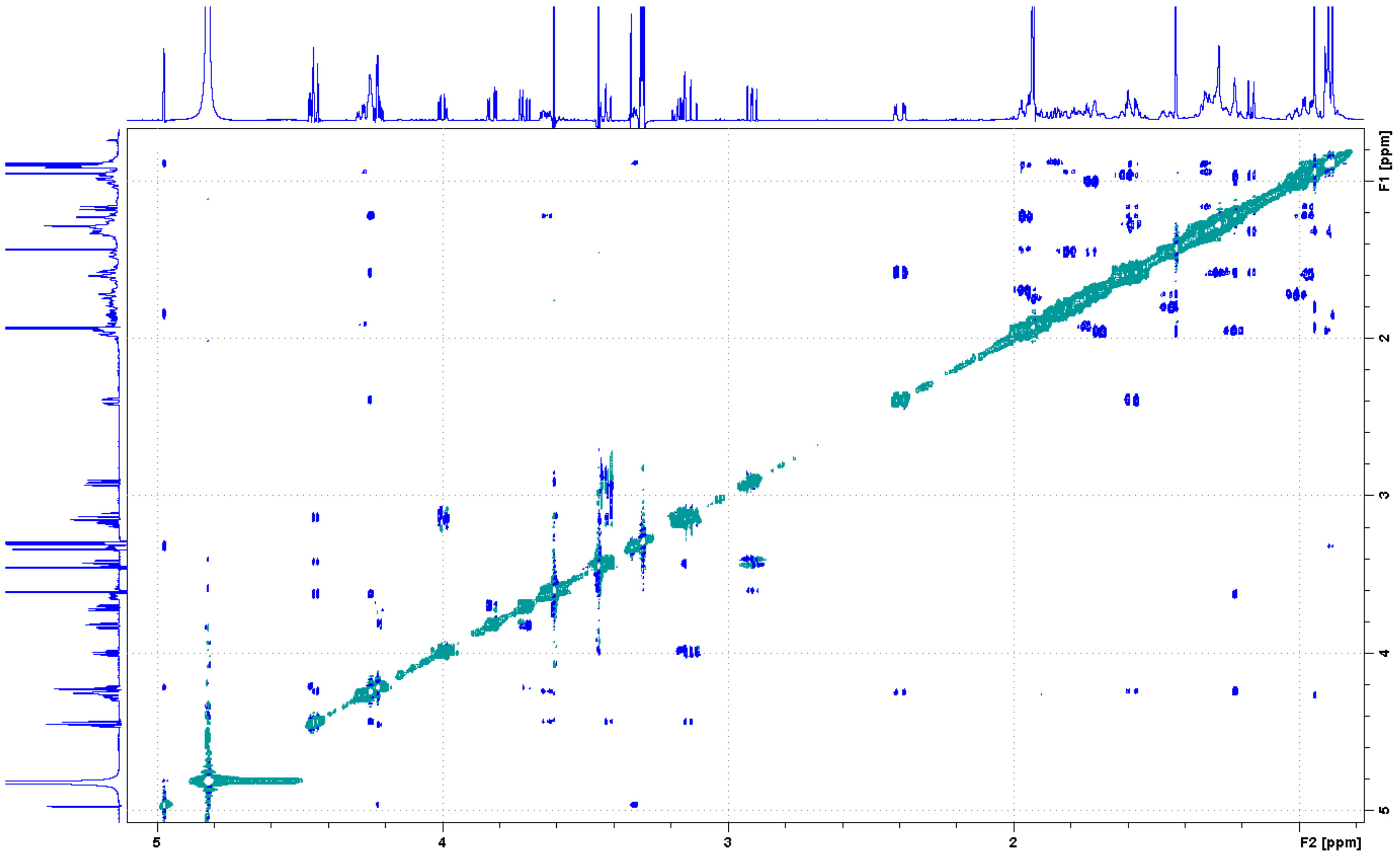

Figure S17. HRESIMS and HRESIMS/MS spectra of compound **2a**.

(–)HRESIMS: [M – H]$^-$, [M +AcH]$^-$ ions

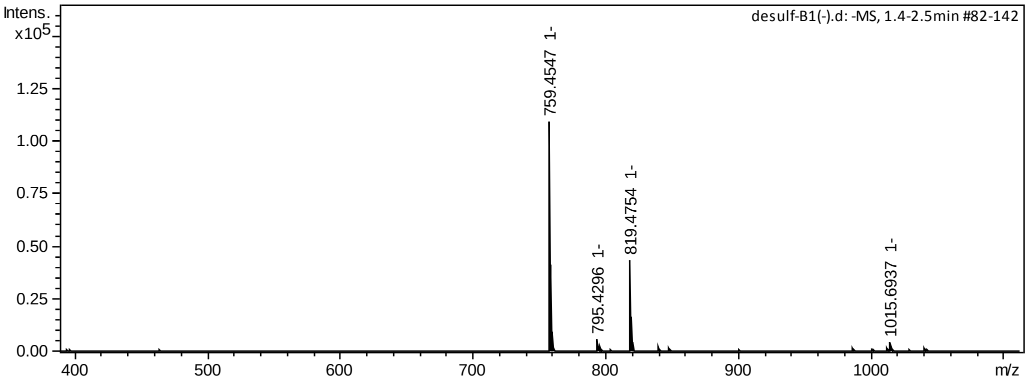

(+)HRESIMS: [M + Na]$^+$ ion

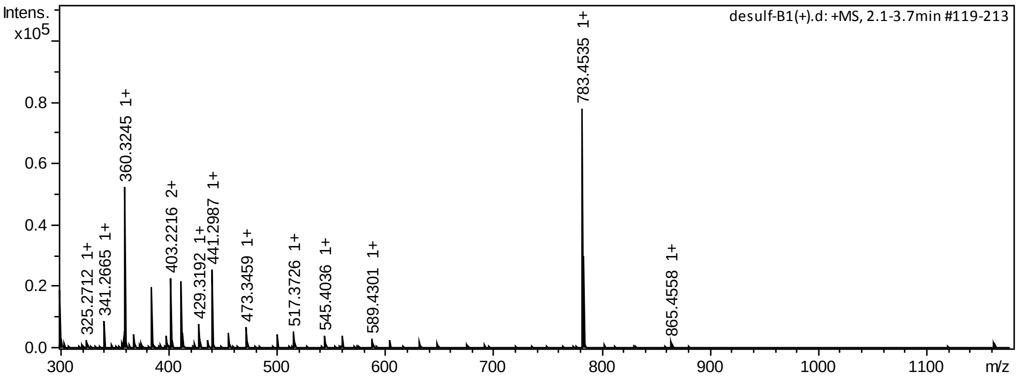

(–)HRESIMS/MS of the [M – Na]$^-$ ion at *m/z* 759

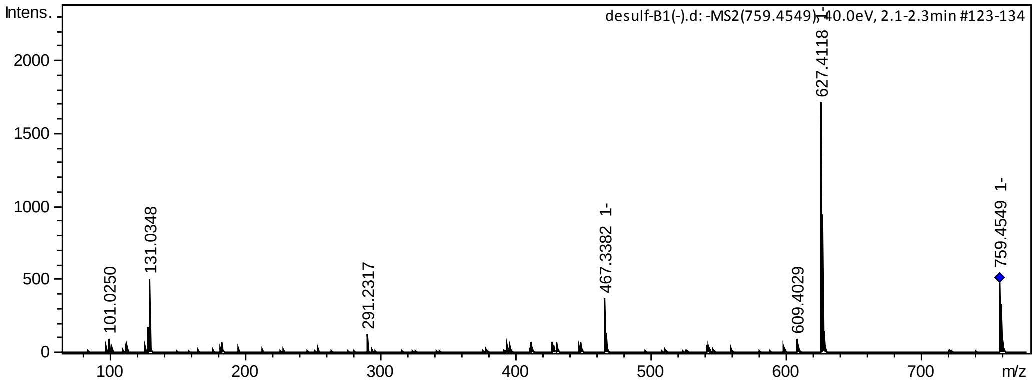

(+)HRESIMS/MS of the [M + Na]$^+$ ion at *m/z* 783

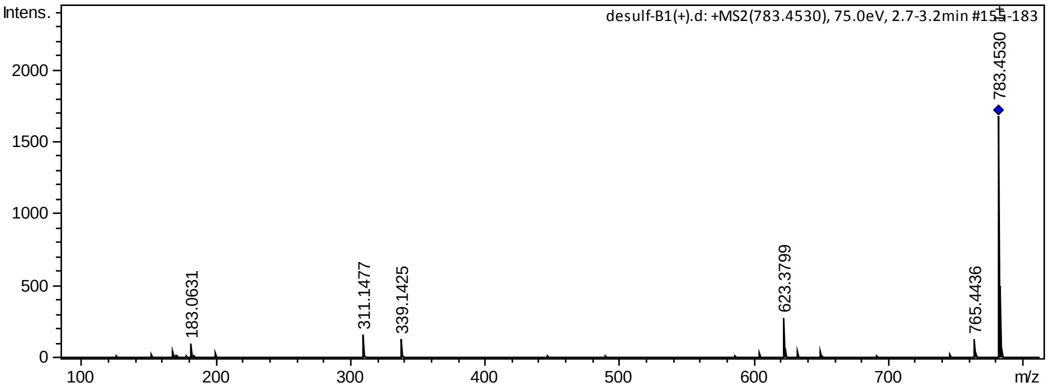

Figure S18. ^{1}H-NMR (700.13 MHz, CD_3OD) spectrum of compound **2a**.

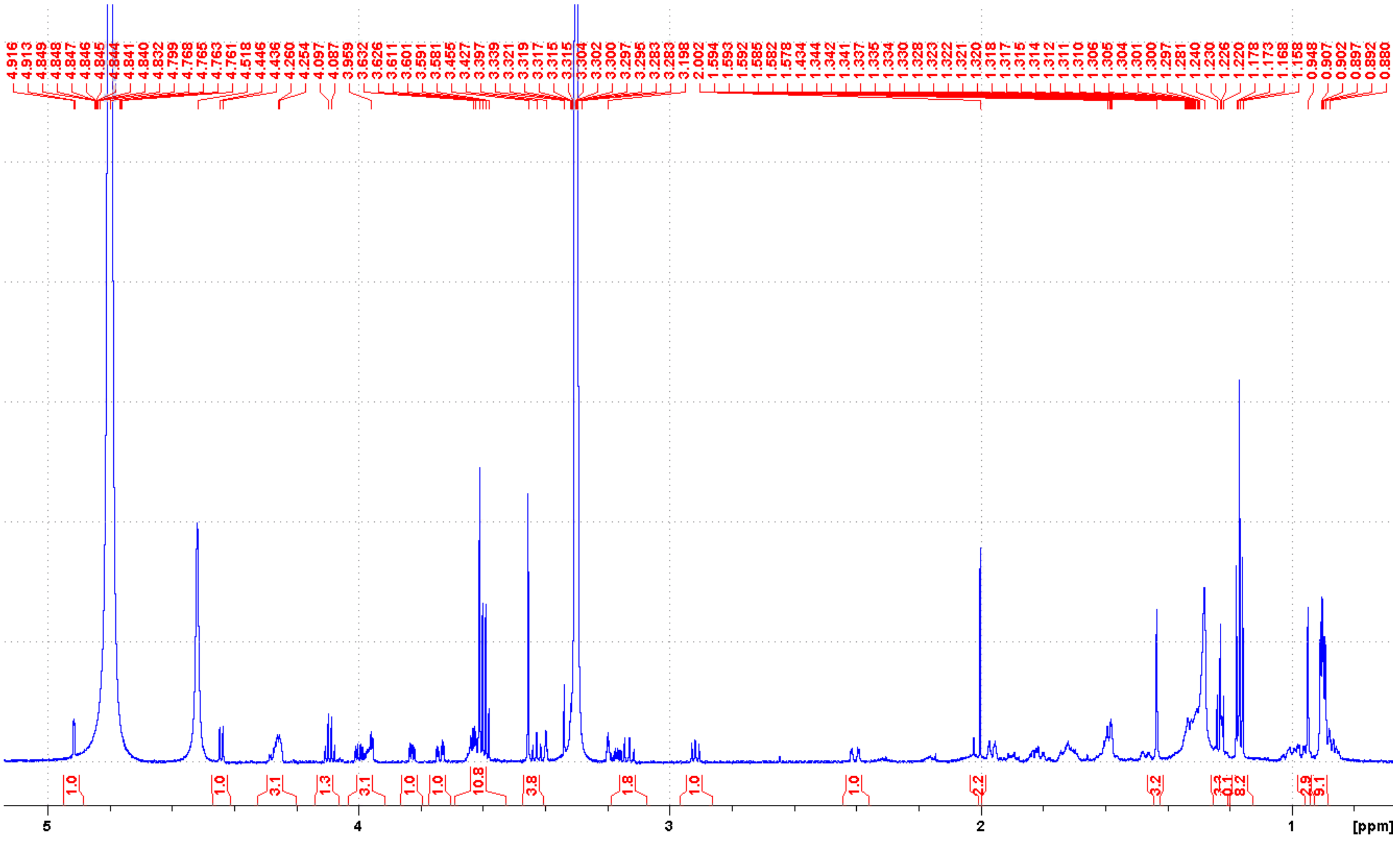

Figure S19. ^{13}C-NMR (176.04 MHz, CD_3OD) spectrum of compound **2a**.

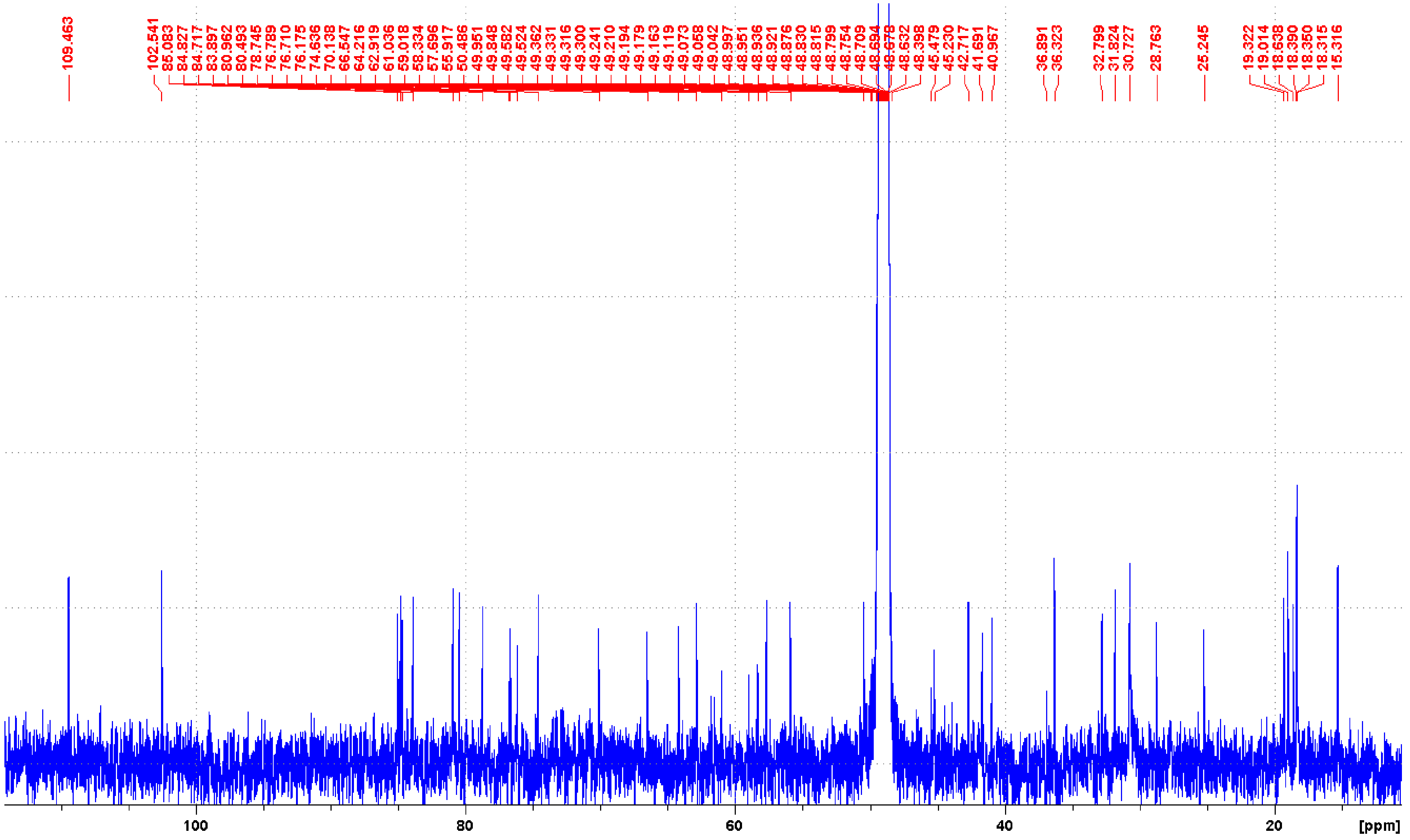

Figure S20. HSQC (700.13 MHz, CD_3OD) spectrum of compound **2a**.

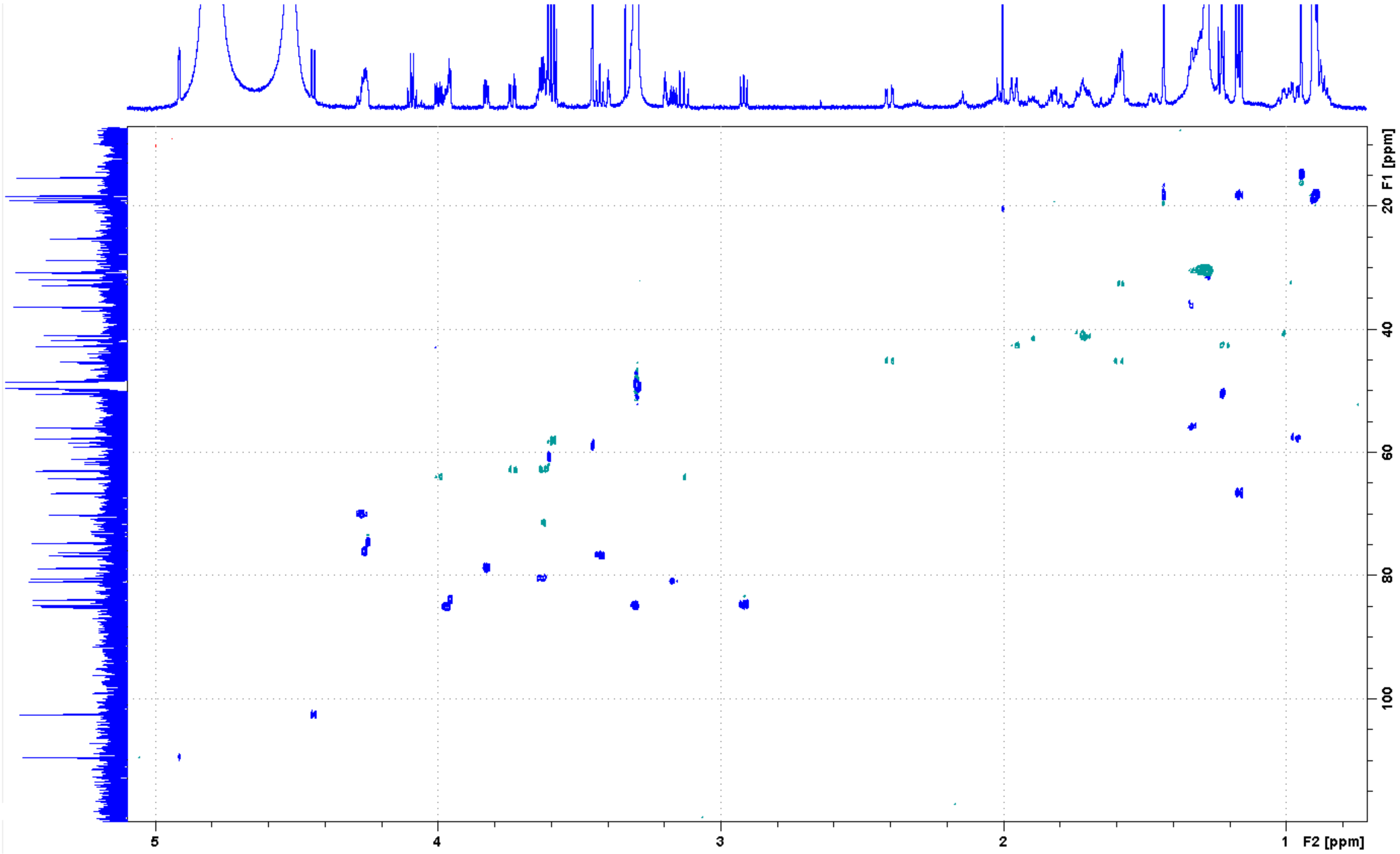

Figure S21. HRESIMS and HRESIMS/MS spectra of compound **3**.

(–)HRESIMS: [M – Na]$^{-}$ ion

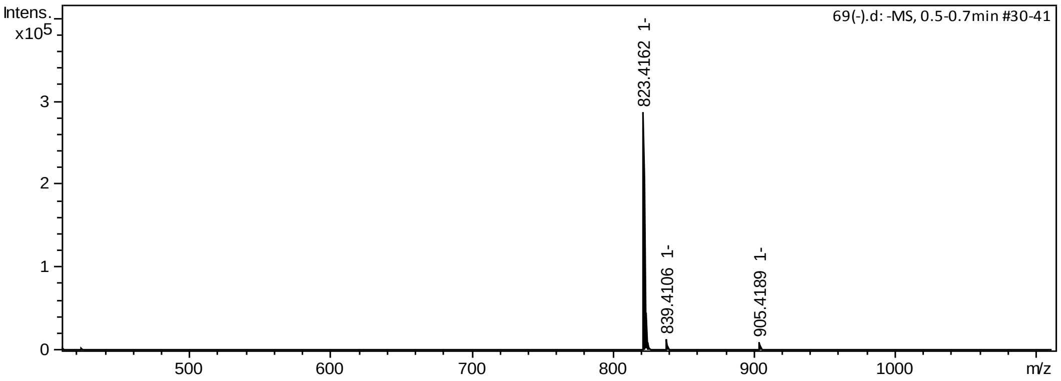

(+)HRESIMS: [M + Na]$^{+}$ ion

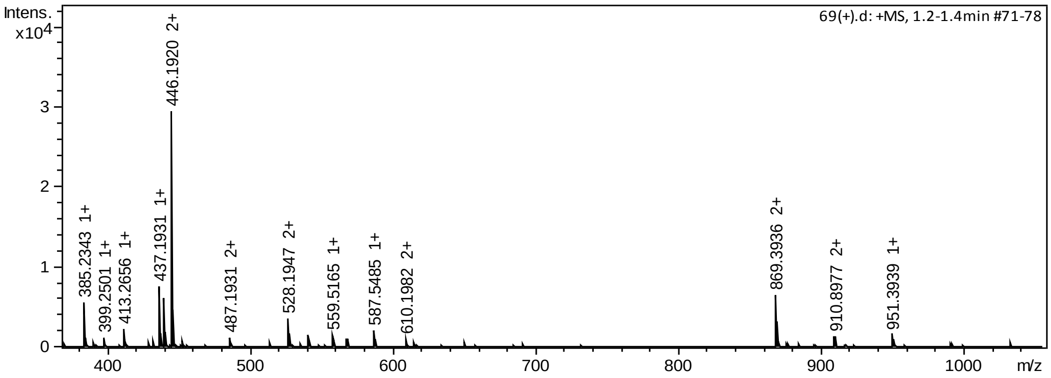

(–)HRESIMS/MS of the [M – Na]$^{-}$ ion at *m/z* 823

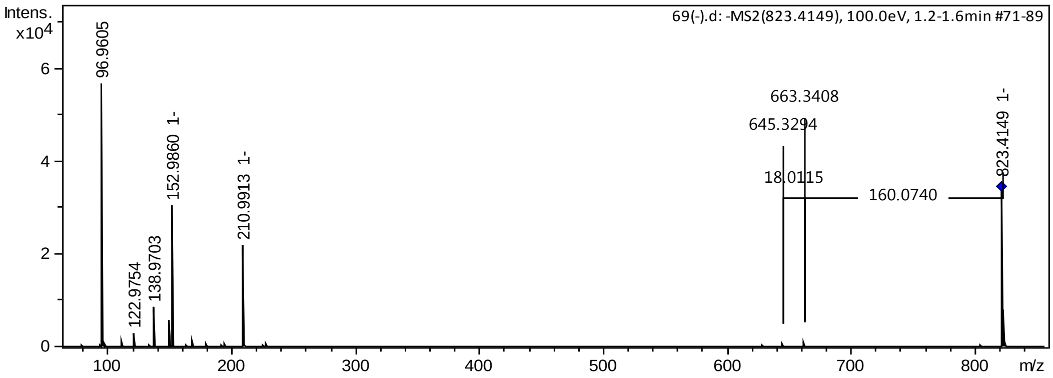

(+)HRESIMS/MS of the [M + Na]$^{+}$ ion at *m/z* 869

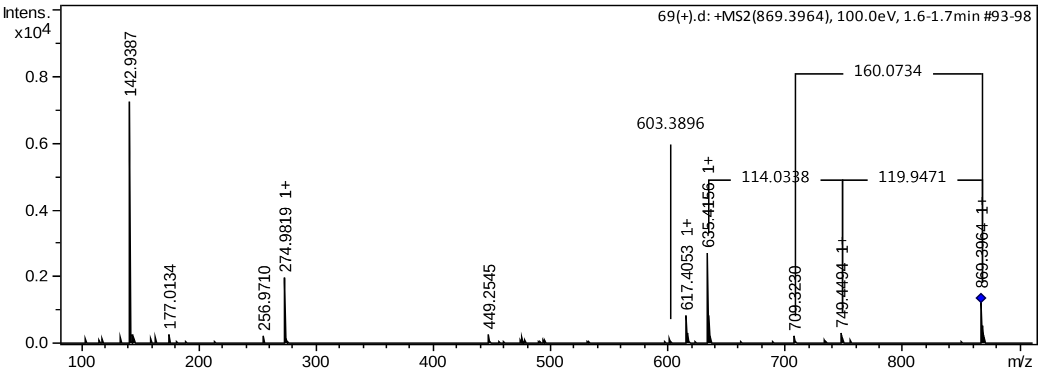

Figure S22. ^{1}H-NMR (500.13 MHz, CD_3OD) spectrum of compound **3**.

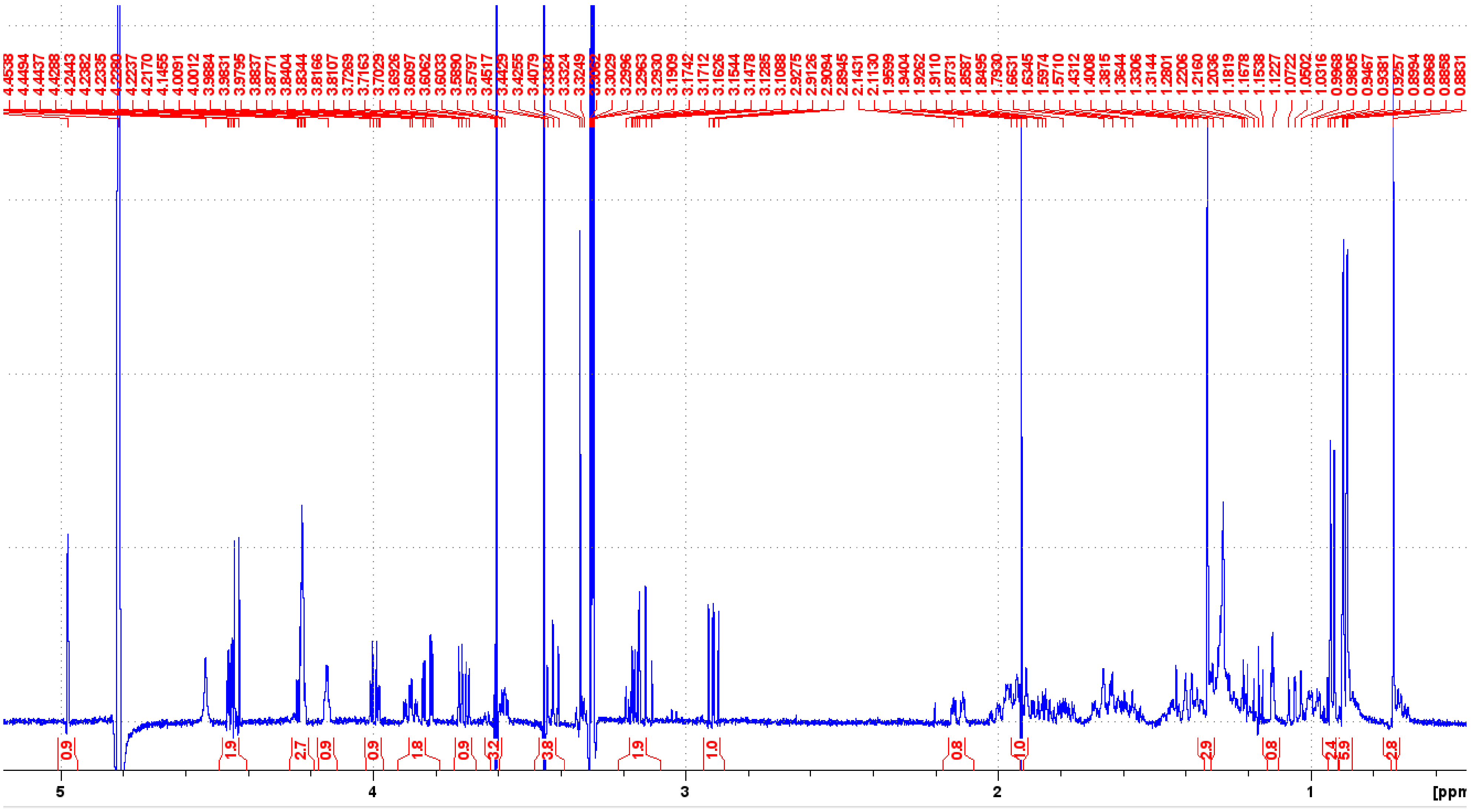

Figure S23. ^{13}C-NMR (125.76 MHz, CD_3OD) spectrum of compound **3**.

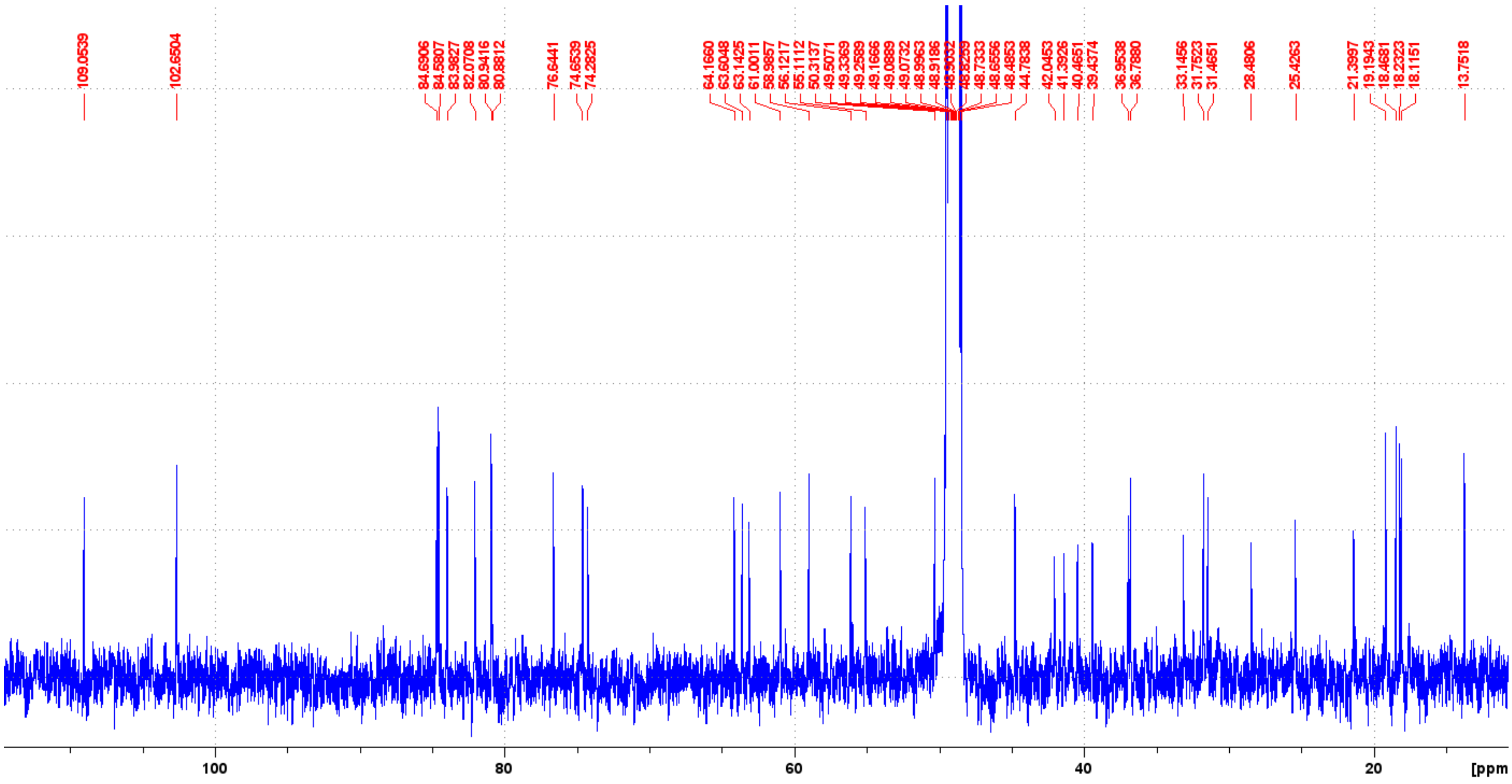

Figure S24. DEPT (125.76 MHz, CD_3OD) spectrum of compound **3**.

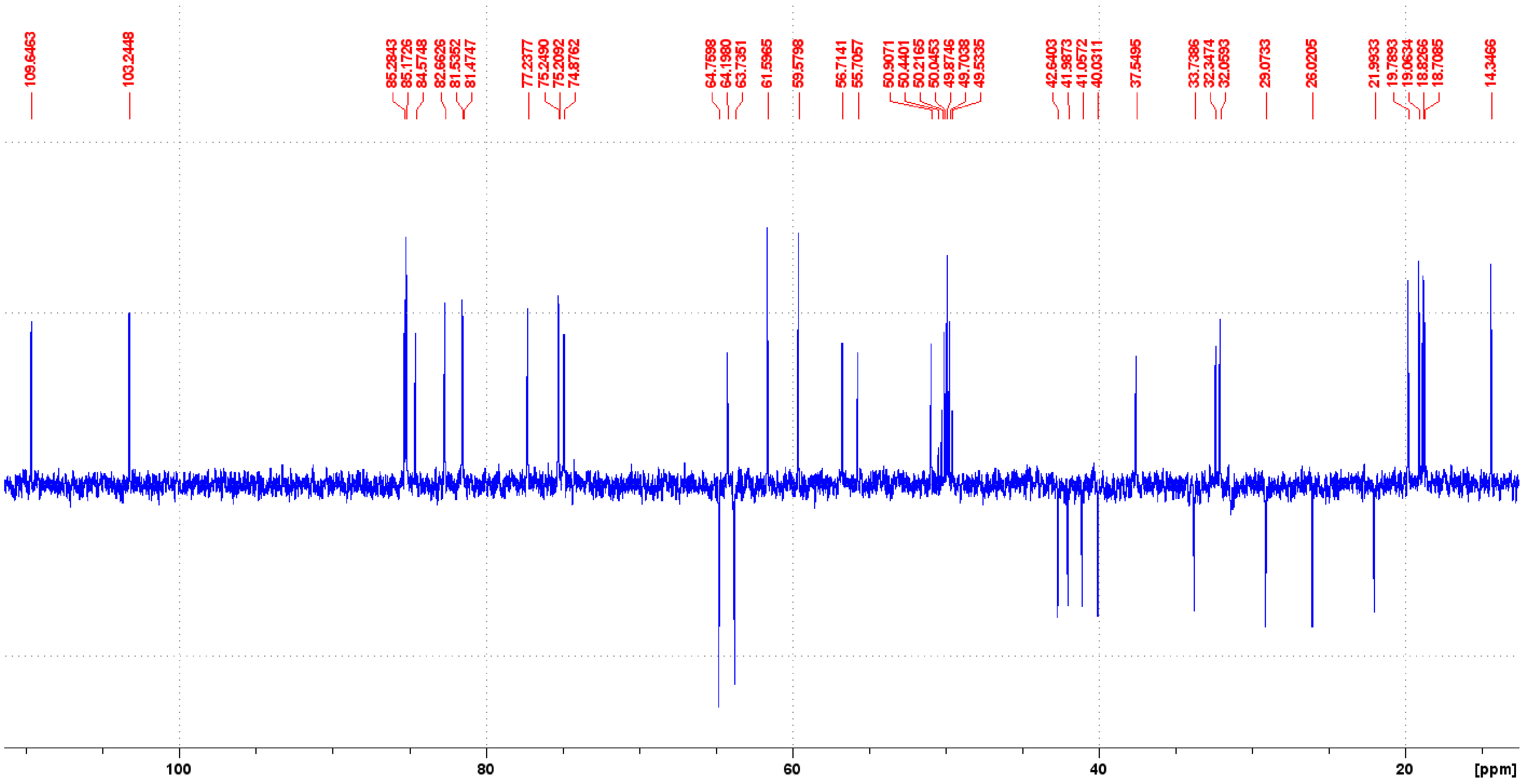

Figure S25. ^{1}H-^{1}H COSY (500.13 MHz, CD_3OD) spectrum of compound **3**.

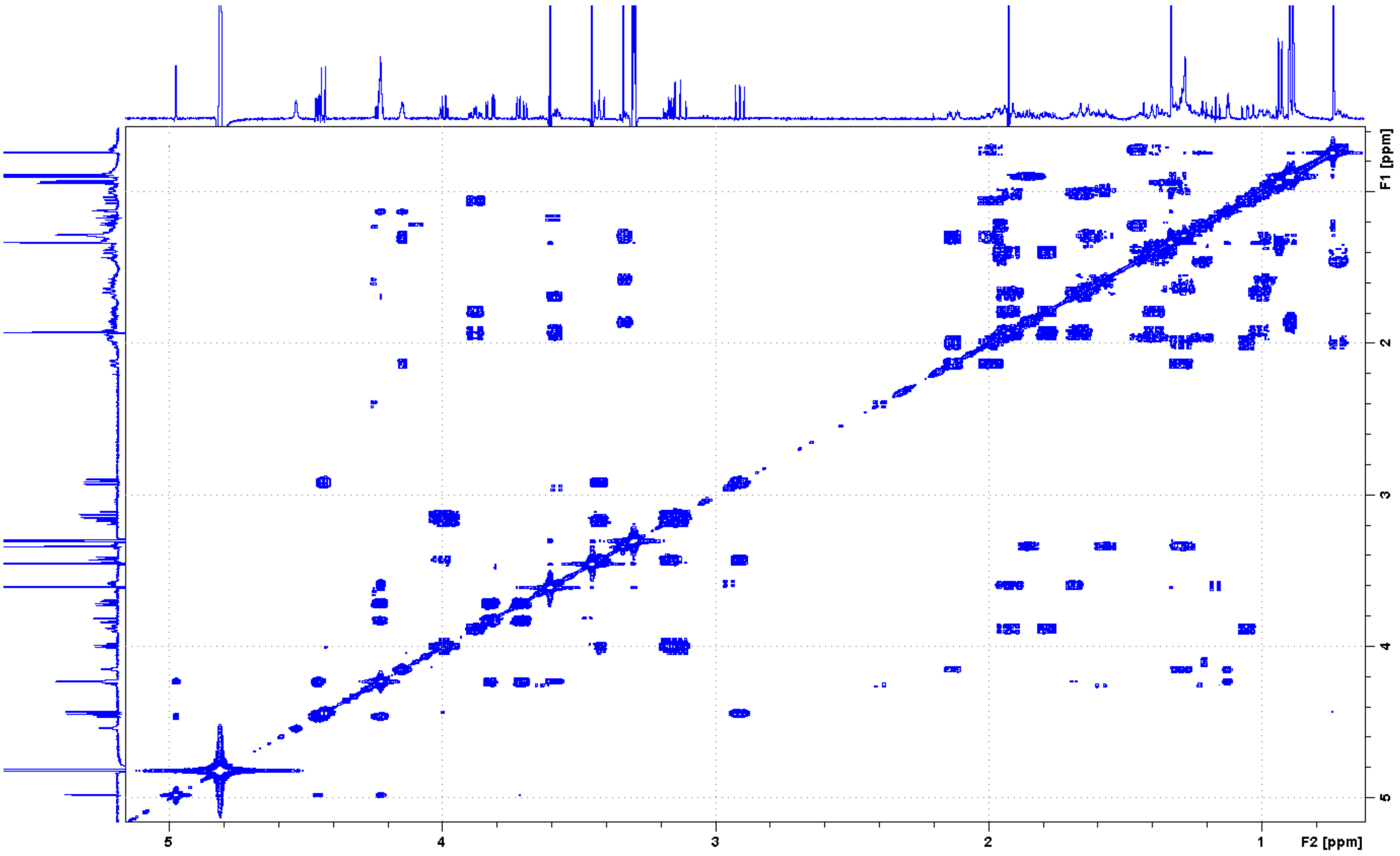

Figure S26. HSQC (500.13 MHz, CD_3OD) spectrum of compound **3**.

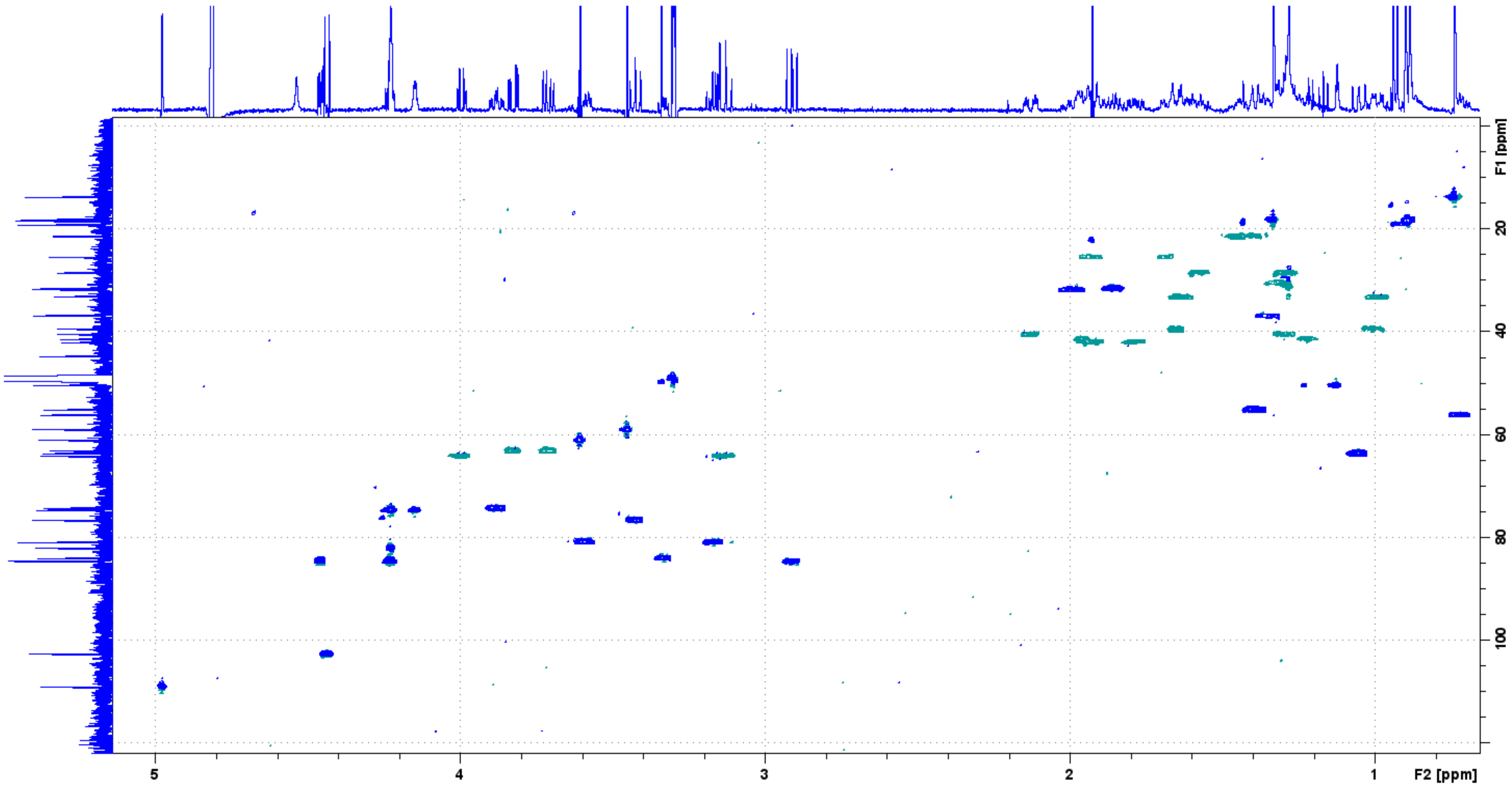

Figure S27. HMBC (500.13 MHz, CD_3OD) spectrum of compound **3**.

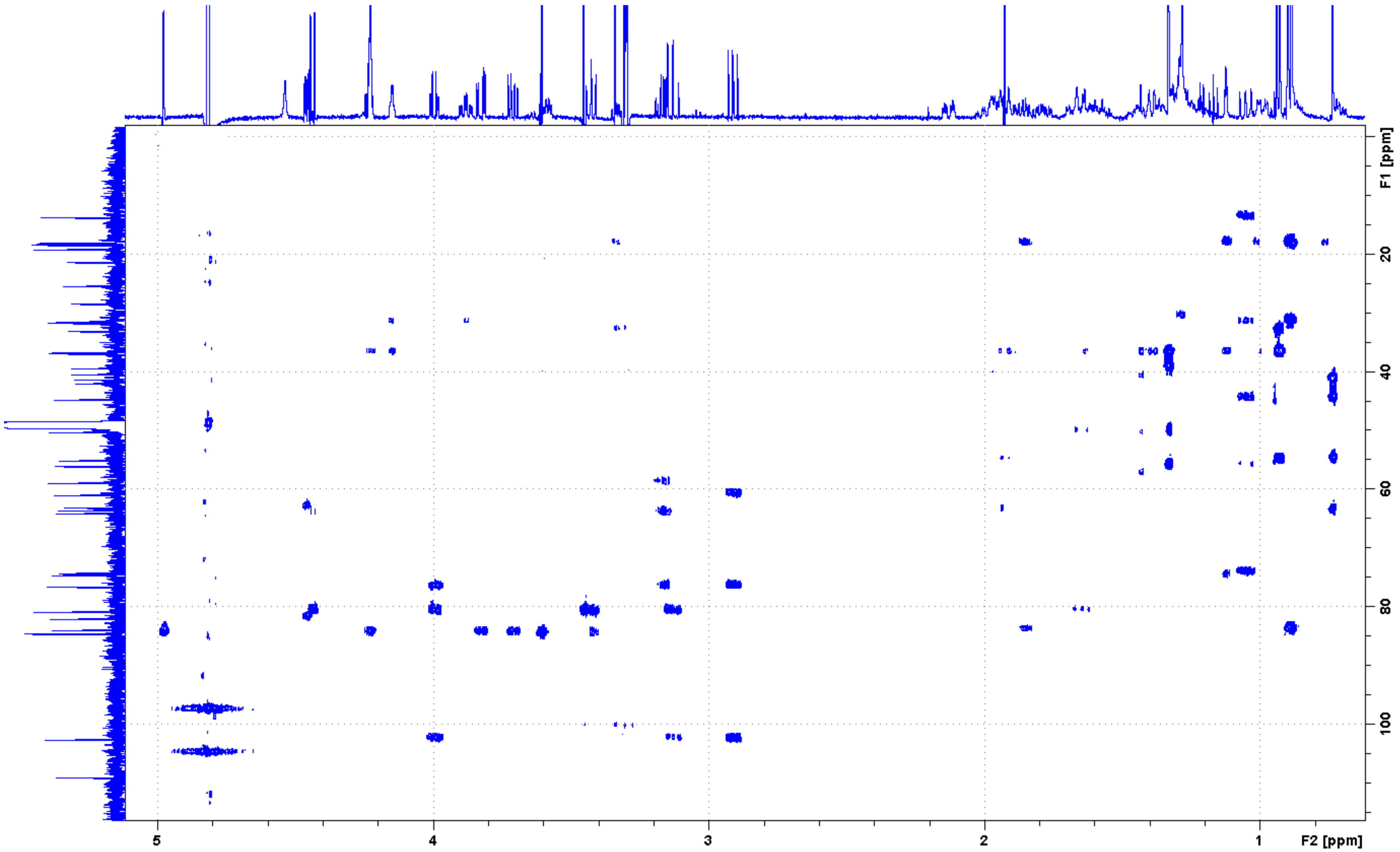

Figure S28. ROESY (500.13 MHz, CD_3OD) spectrum of compound **3**.

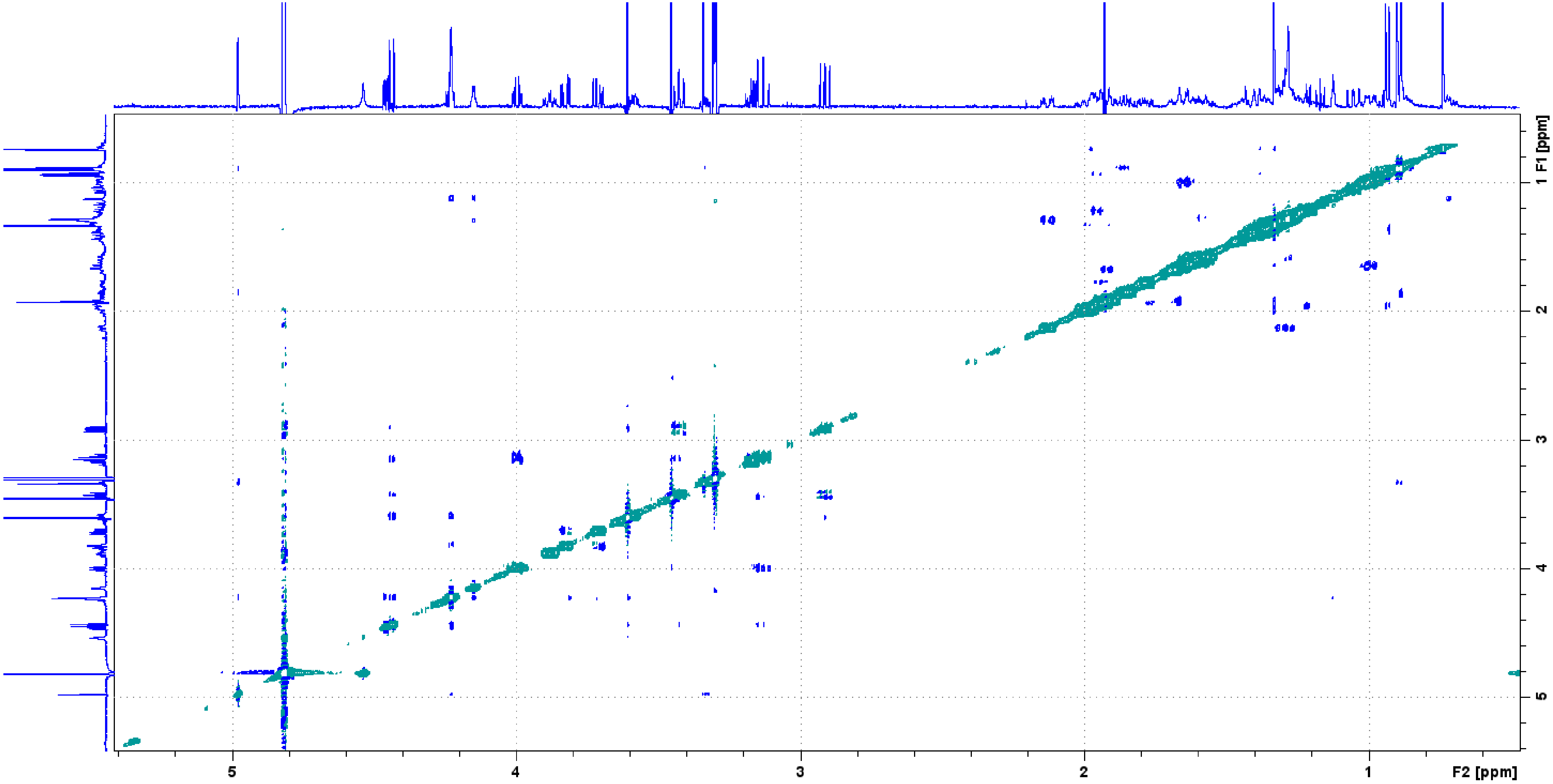

Figure S29. HRESIMS and HRESIMS/MS spectra of compound **4**.

(–)HRESIMS: [M – H]$^{-}$, [M + Cl]$^{-}$, [M + CHO_2]$^{-}$ ions

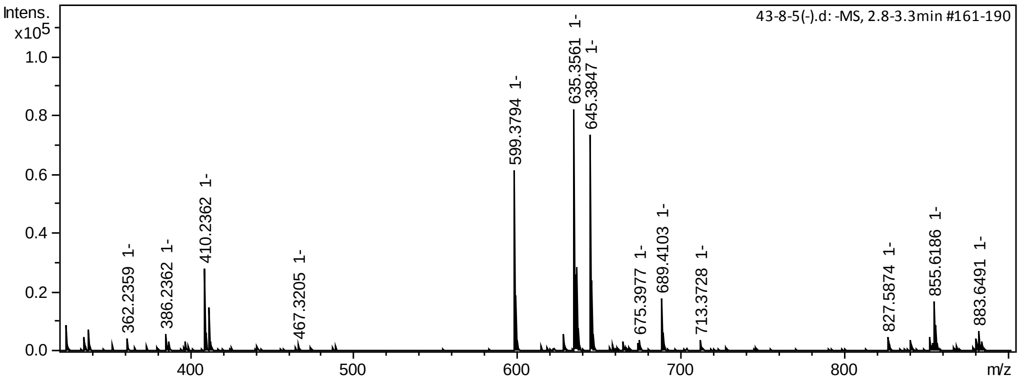

(+)HRESIMS: [M + Na]$^{+}$ ion

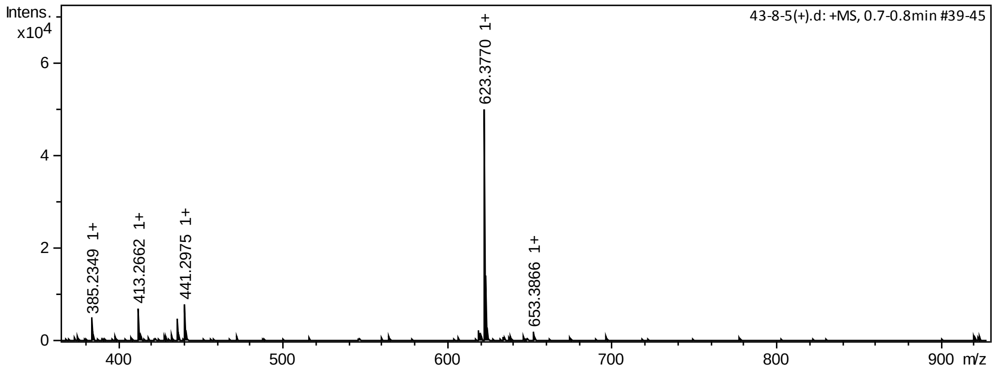

(–)HRESIMS/MS of the [M – H]$^{-}$ ion at *m/z* 599

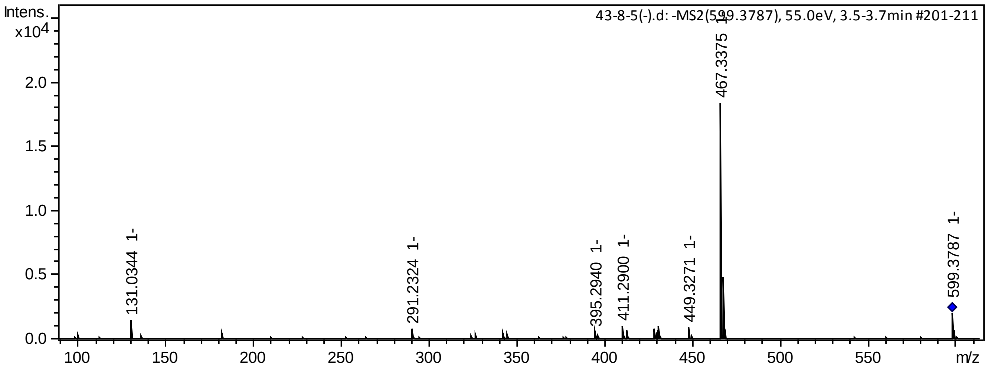

(+)HRESIMS/MS of the [M + Na]$^{+}$ ion at *m/z* 623

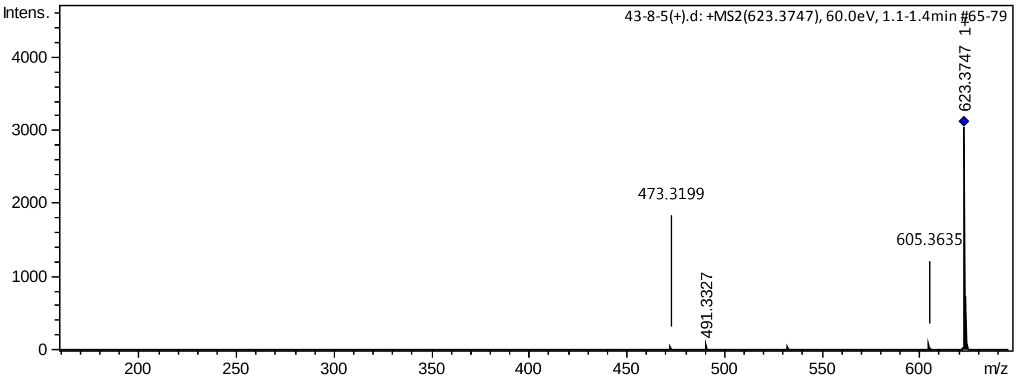

Figure S30. ^{1}H-NMR (700.13 MHz, CD_3OD) spectrum of compound **4**.

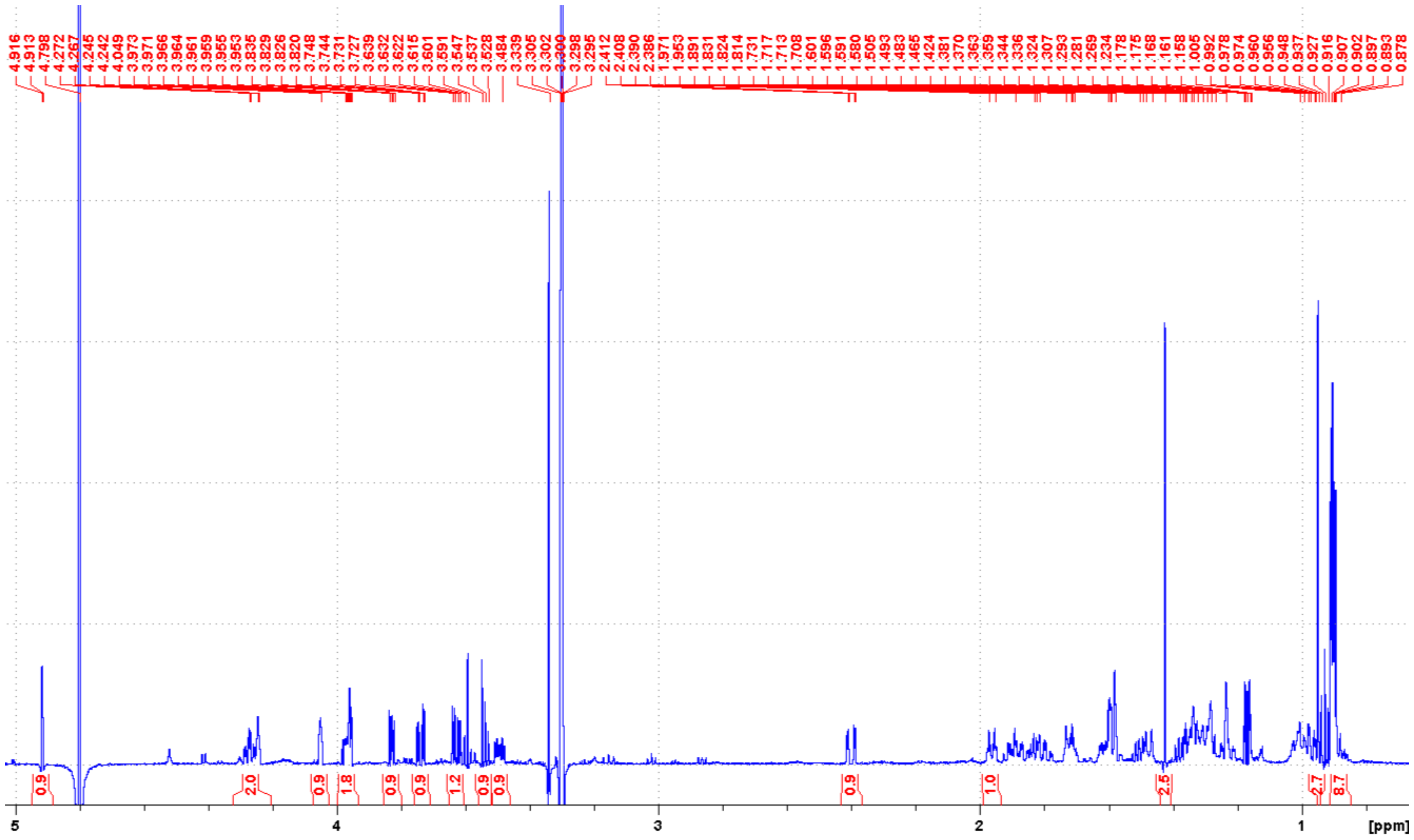

Figure S31. ^{13}C-NMR (176.04 MHz, CD_3OD) spectrum of compound **4**.

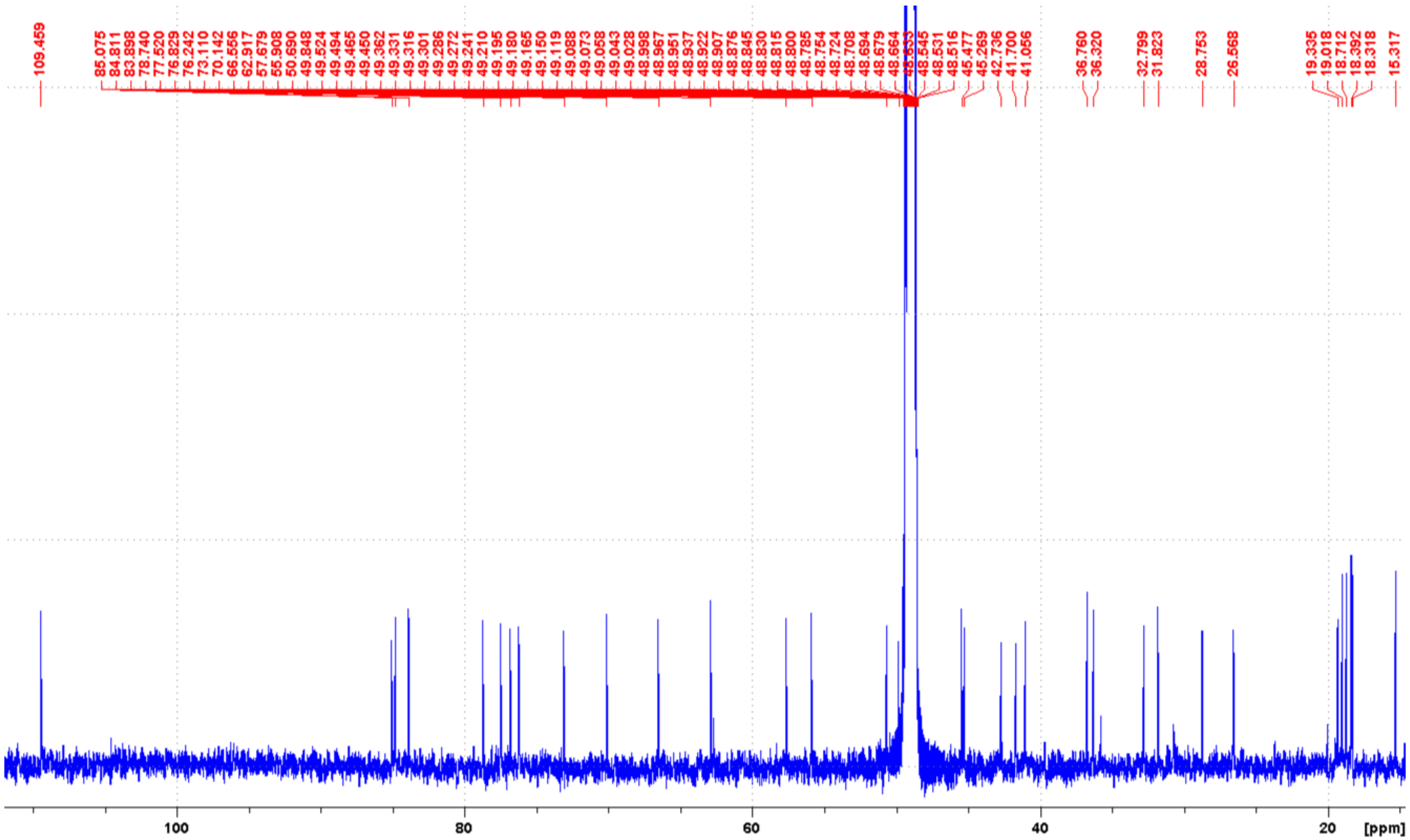

Figure S32. DEPT (176.04 MHz, CD_3OD) spectrum of compound **4**.

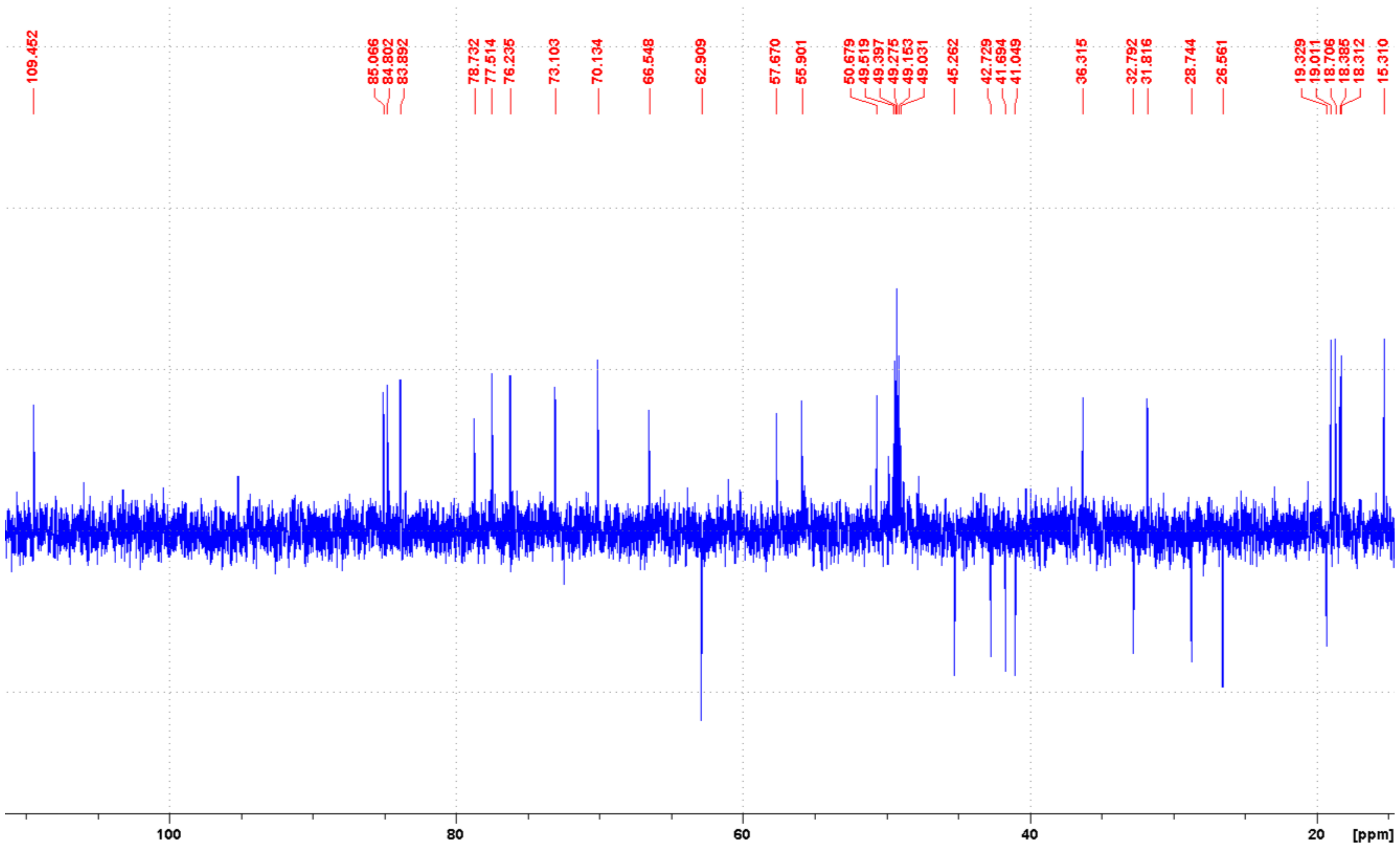

Figure S33. ^{1}H-^{1}H COSY (700.13 MHz, CD_3OD) spectrum of compound **4**.

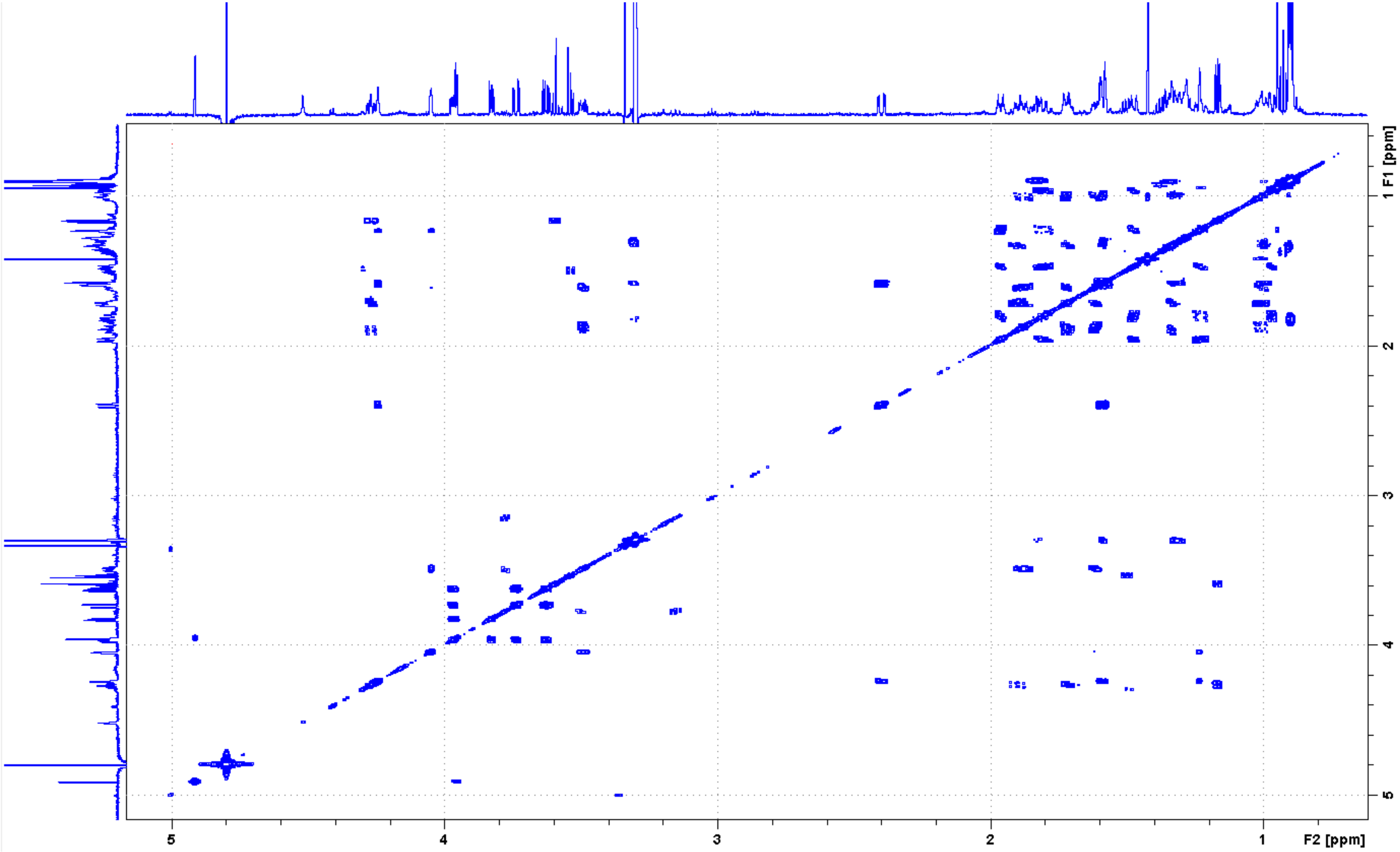

Figure S34. HSQC (700.13 MHz, CD_3OD) spectrum of compound **4**.

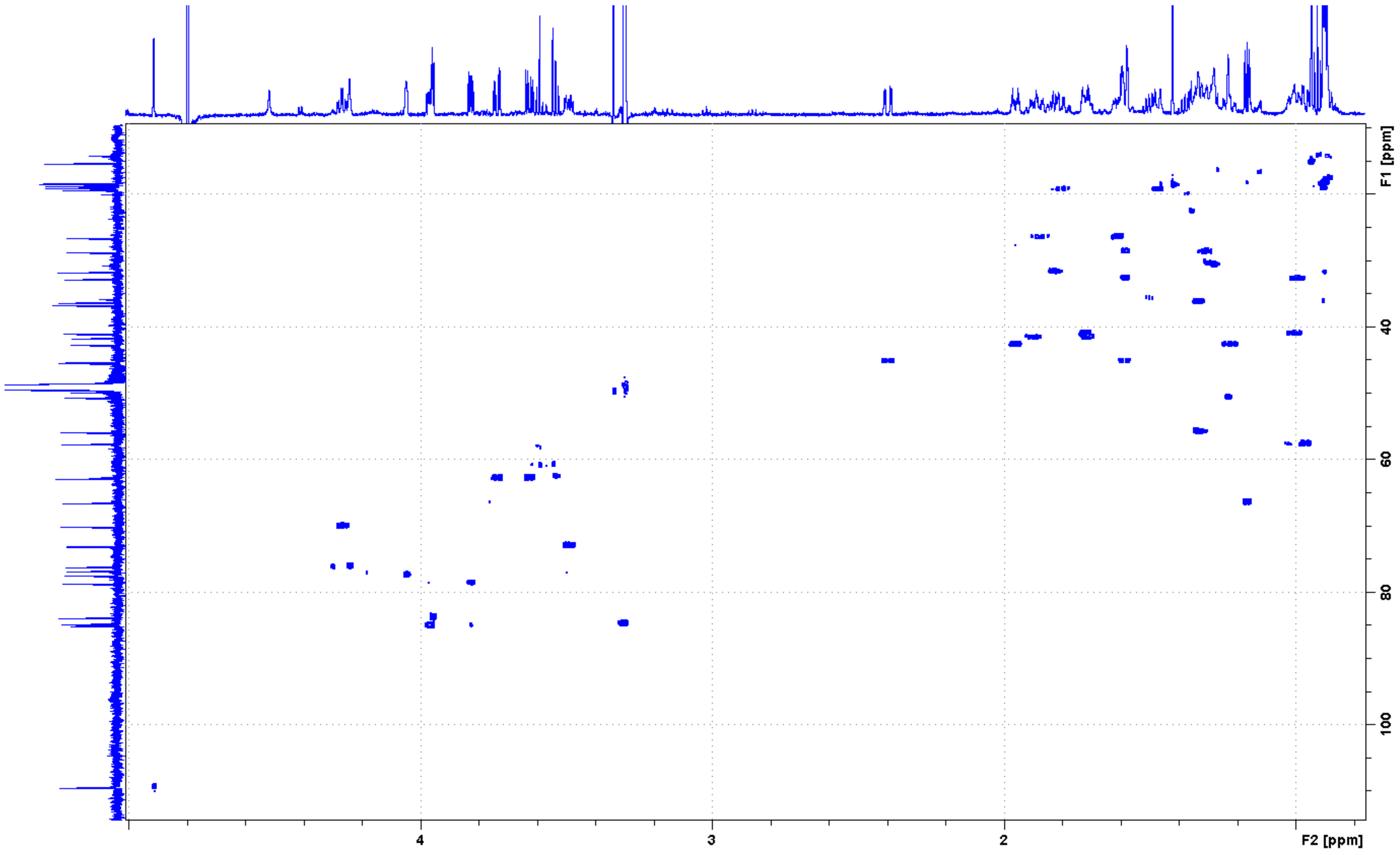

Figure S35. HMBC (700.13 MHz, CD_3OD) spectrum of compound **4**.

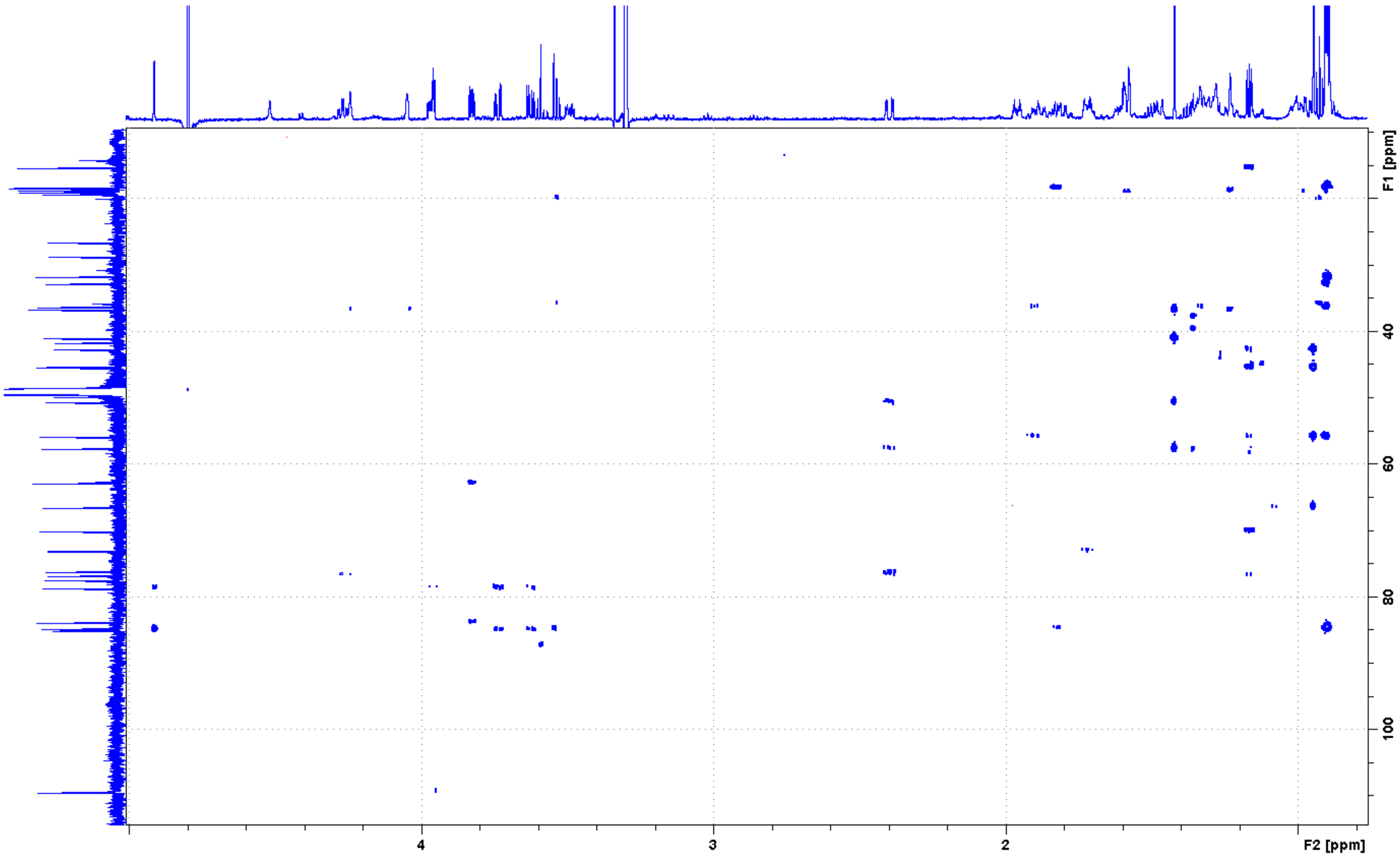

Figure S36. ROESY (700.13 MHz, CD_3OD) spectrum of compound **4**.

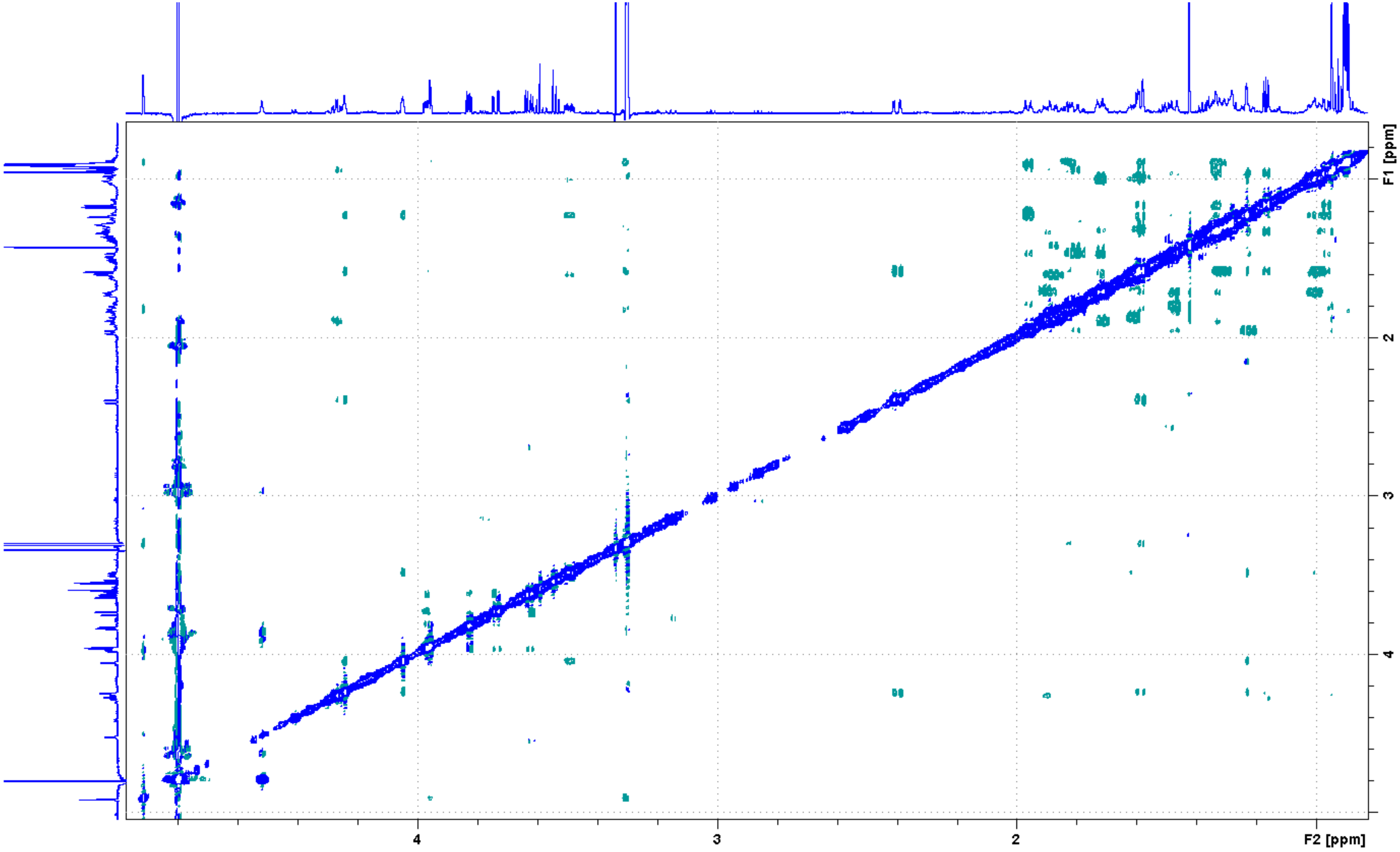

Supplement: Supplementary file 1 [file marinedrugs-22-00294-s001.zip › marinedrugs-3075378-supplementary.pdf]
